# An Integrative Revision of the Genus *Rhamphus* (Curculionidae) from the Western Palearctic: Morphological and Molecular Data Reveal the Radiation of Multiple Species †

**DOI:** 10.3390/insects16111123

**Published:** 2025-11-03

**Authors:** Ivo Toševski, Roberto Caldara, Jelena Jović, Cosimo Baviera, Iñigo Ugarte San Vicente, Oliver Krstić

**Affiliations:** 1 CABI Switzerland, Rue des Grillons 1, 2800 Delémont, Switzerland; 2 Institute for Plant Protection and Environment, Banatska 33, 11080 Zemun, Serbia; jovic_biolab@yahoo.com (J.J.); oliverk13@yahoo.com (O.K.); 3 Center of Alpine Entomology, University of Milan, 20122 Milan, Italy; roberto.caldara@gmail.com; 4 Department of Chemical, Biological, Pharmaceutical and Environmental Sciences, University of Messina, 98122 Messina, Italy; baviera.cosimo@gmail.com; 5 Museo de Ciencias Naturales de Álava, 01001 Vitoria-Gasteiz, País Vasco, Spain; orobitis@gmail.com

**Keywords:** Curculionidae, *Rhamphus*, systematics, taxonomy, phylogeny, morphology, species limit, new species, dichotomous key

## Abstract

**Simple Summary:**

Weevils from the genus *Rhamphus* [Clairville], 1798 (Rhamphini, Curculionidae) are a poorly studied group in the western Palearctic fauna. On the basis of the integrative taxonomy approach, we identified subtle morphological differences and significant molecular divergence by analyzing three genetic markers, one mitochondrial *COI*, and two nuclear genes, n*EF-1α* and n*CAD*. We defined eight morphotypic groups and 14 species within the genus *Rhamphus* in the western Palearctic, of which 6 are newly described in this paper. Our study reveals the complexity of the taxonomy and species evolution within the genus *Rhamphus* in the western Palearctic, where some of the species groups exhibit cryptic species radiation; consequently, more undescribed species should be expected in further study.

**Abstract:**

Here, we report on the complexity of the taxonomy and species evolution within the monophyletic genus *Rhamphus*, which includes some of the smallest members of the Curculionidae family and whose species are morphologically almost indistinguishable from each other. Despite their similar appearance, we found high divergence and varying evolutionary rates among observed species groups living both in sympatry and allopatry in the western Palearctic. On the basis of subtle morphological differences and molecular evidence, we defined eight morphotypic groups and 14 species, of which 6 are newly described in this paper: *R. diottii* **sp. nov**. and *R. ibericus* **sp. nov.** (*monzinii*-group), *R. cypricus* **sp. nov.** and *R. macedonicus* **sp. nov.** (*cypricus*-group), *R. betulae* **sp. nov.** and *R. crypticus* **sp. nov.** (*pulicarius* group). *Rhamphus* morphotypic groups showed intense species radiation and cryptic speciation, with an estimated genetic divergence of 4.2–18.8% (uncorrected) in the barcoding region of the mitochondrial *COI* gene. The estimated divergence of the two nuclear markers, n*EF-1α* and n*CAD*, ranged from 1 to 11.9% and 0.5 to 15%, respectively. Phylogenetic analyses using both single and partitioned multigene adequately resolved the relationships between *Rhamphus* species and identified all groups and the species with high nodal support. According to our study, *Rhamphus* species cluster into monophyletic groups that are partly defined by their host plant associations and by subtle differences in penis shape. No substantial differences in female genitalia were found. Most of the species exhibit relatively rapid species radiation, which is cryptic by nature.

## 1. Introduction

The leaf-miner weevil genus *Rhamphus* [Clairville], 1798—type species: *R. oxyacanthae* (Marsham, 1802)—is characterized mainly by a rostrum that is usually retracted between forecoxae in thanatosis, approximately as long as the prosternum; antennae inserted between forehead and base of rostrum, not geniculate; and large metafemora modified for jumping. The genus belongs to the tribe Rhamphini (Curculionidae, Curculioninae) which, according to Alonso-Zarazaga [1], comprises 13 genera with 153 species in the Palearctic region. The genus *Rhamphus* comprises approximately 45 species worldwide except for the Americas [1,2,3,4,5,6,7]. Fifteen of them live in the Palearctic Region, of which eight are known from Europe and North Africa [8,9,10,11].

The taxonomic classification of *Rhamphus* species has proven difficult because of their similarity, given that there are only a few morphologically distinguishable characters for the males based on the shape of the penis and the absence of substantial differences in female genitalia (spermatheca and spiculum ventrale). If we consider that *Rhamphus* species are some of the smallest weevils in the family, with a usual length range of 1–2 mm, problems in species identification are permanently present because a revisional study of this genus has never been performed. In sparse faunistic records, only three species are commonly reported, *Rhamphus oxyacanthae* associated with diverse Rosaceae plants, *R. pulicarius* (Herbst, 1975) associated with Salicaceae and Betulaceae and *R. subaeneus* Illiger, 1808 associated with *Crataegus* spp. (Rosaceae), where the latter is easily recognizable by metallic bronze–green shine on elytra. Following typical morphological characters, the genus *Rhamphus* is considered as a well-defined monophyletic group with particular difficulties in distinguishing species within this genus; thus, the definition of cryptic species [12] seems particularly appropriate in this case.

In recent decades, few studies have provided interesting insights into *Rhamphus* fauna in the western Palearctic. This resulted in the description of three new species in a relatively short time [8,9,10]. More recently, Diotti [11] described two additional species from the Italian isles of Sicily and Sardinia with very subtle morphological differences from the known taxon *R. oxyacanthae*. The description was based on an integrated taxonomic approach, combining the morphological and molecular data of the newly described species and related taxa in the genus. Molecular data strongly supported subtle morphological differences, revealing cryptic speciation events among the studied populations. In addition, the evidenced existence of cryptic species within the genus *Rhamphus* generated a need for stabilization of the taxonomic and nomenclature issues of *Rhamphus* spp., especially after numerous errors were recorded in the literature, including published molecular data in the BOLD and NCBI GenBank databases [13]. Thus, fixation of the taxonomic status of the most widely distributed western Palearctic taxa established by Caldara [14] was a precondition for the revision of the species from this genus in the western Palearctic. The recent collection of a large number of specimens of *Rhamphus* that we made across the European part of western Palearctic revealed that some specimens showed very subtle morphological differences. More in-depth systematic, morphological, and molecular investigations of this material allowed us to separate six new cryptic species from closely related known species. The main aims of the present study are to describe the species limits and phylogenetic relationships among *Rhamphus* species in the western Palearctic.

## 2. Materials and Methods

### 2.1. Acronyms

The collections housing the material studied in this revision are abbreviated as follows (with their curators in parentheses):

BMNH—British Museum of Natural History, London, UK (M. Barclay)
CBCC—Cesare Bellò collection, Castelfranco Veneto, Italy
CBCM—Cosimo Baviera collection, Messina, Italy
DLCQ—David Lessieur collection, Queyrac, France
GACC—Gabriel Alziar collection, Cassagnes-Bégonhès, France
HMCL—Howard Mendel collection, c/o British Museum of Natural History, London, UK
ITCB—Ivo Toševski collection, Belgrade, Serbia
IUCA—Iñigo Ugarte San Vicente, Agurain/Salvatierra, Spain
JKCH—Jiri Kràtký collection, Hradec Králové, Czech Republic
LFCP—Leonardo Forbicioni collection, Portoferraio, Italy
MHNN—Muséum d’Histoire Naturelle de Nice, Nice, France (G. Lambert)
MNHN—Muséum National d’Histoire Naturelle, Paris, France (H. Perrin)
MSNM—Museo civico di Storia Naturale, Milano, Italy (F. Rigato)
MTCM—Michele Tedeschi collection, Milano, Italy
PSCC—Peter E. Stüben collection, Curculio Institute, Mönchengladbach, Germany.
RCCM—Roberto Caldara collection, Milano, Italy
SMCM—Sergio Monzini collection, Milano, Italy
RGCL—Rafał Gosik collection, Lublin, Poland

### 2.2. Insect Sampling

**Figure 1:**
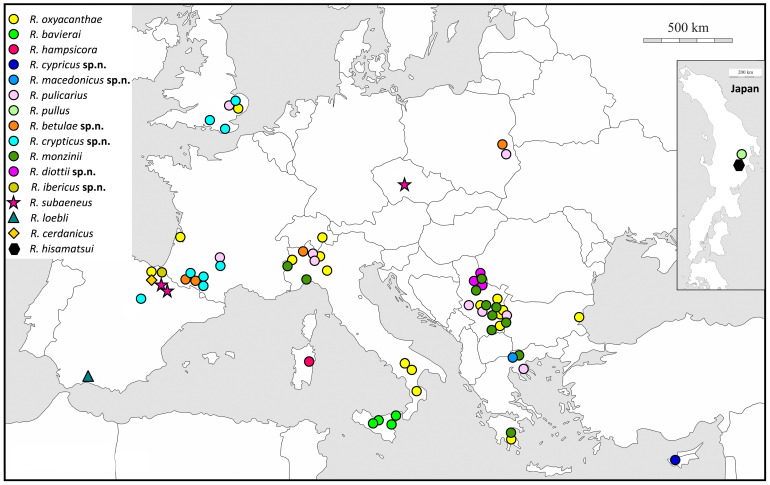
Sampling sites for *Rhamphus* species used for genetic analysis. Map from d-maps.com (https://d-maps.com/carte.php?num_car=2232&lang=en, accessed on 10 December 2024).

We sampled more than 400 individuals belonging to the genus *Rhamphus* across a broad geographic and host plant range of the western Palearctic (Figure 1) from 21 different host plants belonging to the families Rosaceae, Salicaceae, Betulaceae, and Cistaceae (Table S1). In addition, several hundred specimens were examined to estimate the geographical distributions of the studied taxa. This study was based on the examination of type specimens when available, specimens from private and public collections, and mainly specimens from newly collected material (Table S1).

The weevils were collected using either the sweep-net method or by beating branches on a white umbrella-shaped surface. On some occasions, leaves with mines were collected in late autumn and placed on the soil surface in plastic containers for overwintering. Emerging adults were collected in early spring from the rearing cylinders. In addition, two species with eastern Palearctic distributions (Japan) were subjected to analysis, *Rhamphus pullus* Hustache, 1920 and *Rhamphus hisamatsui* Chûjô & Morimoto, 1960, which were collected by sweeping from *Betula* sp. and *Acer pictum* ssp. *mono* (Sapindaceae), respectively. All the collected specimens were labeled, placed individually in 96% ethanol, and stored at −20 °C until DNA extraction. After extraction, the sampled material was prepared on cardboard plates as dry voucher specimens. Type specimens of newly described species are deposited in the corresponding national collections or in the collection of the Natural History Museum, London (Great Britain). The remainder of the material sampled for this study was deposited in the private collection of the collectors and the first and second authors.

### 2.3. Morphological Analysis

In the diagnostic description, we summarized the characters that allow more or less easy identification of all the taxa, on the basis of morphology and the shape of the penis in the dorsal and lateral views. The characteristics common to all the species of the genus were avoided. No substantial differences were observed in female genitalia (spermatheca and spiculum ventrale) for each species treated herein, thus confirming their cryptic nature. For terminology, we followed the online glossary of weevil characters proposed on the International Weevil Community Website (http://weevil.info/glossary-weevil-characters, accessed 20 March 2025), edited by C.H.C. Lyal (The Natural History Museum, London, UK). Photographs were taken with a Leica MC170 HD digital camera on a Leica M165C stereomicroscope (Wetzlar, Germany). SEM images were taken from the uncoated specimens using a backscattered electron signal with a JEOL SM5610 LV (JEOL, Tokyo, Japan) scanning electron microscope (SEM) at the Natural History Museum of Milan (Italy).

The same stereoscopic microscope (Leica M165C, Wetzlar, Germany) was used to measure the weevils. Body length was considered the distance from the anterior margin of the pronotum along the midline to the apex of the elytra. We measured the length (Pl) of the pronotum along the midline from the apex to the base and its width (Pw) across the widest point, whereas the width of the pronotum was expressed as the ratio Pw/Pl. We measured the length of the elytra (El) along the midline from the transverse line joining the most anterior point of the humeri to the apex and its width (Ew) across the widest point. We also expressed the proportions of the elytra as the ratios El/Ew and Ew/Pw. The range of variability of the ratios was given only when the low or high value exceeded the reported average by more than 5%.

In the “Type Series” part of Section 3.3, labels are reported verbatim. All the specimens have a further red label with the indication “Holotype (or Paratype) *Rhamphus* (species name) sp. nov. Toševski & Caldara”. A key to the groups of species is reported, whereas a synopsis for the single taxa was preferred, considering it to be more informative in the case of cryptic species. 

### 2.4. Molecular Analysis

For the molecular analysis, we sampled 326 individuals belonging to the genus *Rhamphus* that were collected over a broad range in the western Palearctic (Tables S1–S3). Individual weevils were punctured between the 2nd and 3rd thoracic sternites, and total DNA was extracted from the whole specimen using the QIAGEN Dneasy^®^ Blood & Tissue Kit (Qiagen, Hilden, Germany) according to the manufacturer’s instructions. We amplified and sequenced the barcoding region of the mitochondrial gene cytochrome oxidase 1 (mt*COI*) and the two nuclear protein-encoding genes, i.e., elongation factor-1*α* (n*EF-1α*) and carbamoyl-phosphate synthase domain of the n*CAD* gene (carbamoyl-phosphate synthase 2–aspartate transcarbamylase–dihydroorotase). A cycling protocol was performed using a Mastercycler ep gradient S (Eppendorf, Hamburg, Germany) with the thermal steps and primers [15,16,17,18,19,20] described in Table S4. Details of the primers used for the PCR amplification of the three genetic markers are presented in the Tables S5–S7, Figures S1 and S2. Additionally, for the archival specimens and the specimens with presumably fragmented DNA due to degradation, we designed primers for short fragments for both n*EF-1α* (Table S6, Figure S1) and n*CAD* (Table S7, Figure S2). Sequencing was performed using the commercial services of Eurofins Genomics, Ebersberg, Germany. The sequences were edited with FINCH-TV v.1.4.0 (https://finchtv.software.informer.com, accessed on 27 August 2022) and aligned with CLUSTALW in MEGA7 software [21] according to gene affiliation. The sequences are deposited in the NCBI GenBank database under the accession numbers PV910519–PV910621, PX149871 for the mt*COI* gene, PV943771-PV943836 for the n*EF-1α* gene, and PV930483-PV930532 for the n*CAD* gene. Pairwise analysis (p-distance method) between all recorded haplotypes grouped according to species entities was also conducted using MEGA7 (Table S8). All the details related to the mt*COI*, n*EF-1α*, and n*CAD* genes of the sequenced *Rhamphus* specimens are shown in Tables S1, S2, and S3, respectively.

### 2.5. Evolutionary Tree Construction and Haplotype Networks

Bayesian analyses were performed separately for three genes with the program MrBayes version 3.2.7 [22,23]. For the mt*COI* gene we conducted two simultaneous runs for 10,000,000 generations, sampling every 100 generations, with a heating parameter value of 0.20 and a burn-in frequency of 25%, using the HKY+I+G model determined by jMODELTEST version 2.1.7 and applying the default values and Bayesian Information Criterion [24]. For the n*EF-1α* and n*CAD* genes, we conducted two simultaneous runs for 3,000,000 and 4,000,000 generations, respectively, using substitution model HKY+G determined by jMODELTEST. Additionally, to avoid ambiguities arising from single-marker analyses because of incomplete lineage sorting [25] and introgression [26], we constructed a phylogeny based on partitioned multigene data, where each gene, i.e., partition, was run on a different model of molecular evolution as determined by jMODELTEST. Partitioned data analysis was conducted on two simultaneous runs for 2,000,000 generations, sampling every 1000 generations, with a heating parameter value of 0.20 and a burn-in frequency of 25%, using the selected model of molecular evolution for each partition, i.e., HKY+I+G, K80+I+G, and K80+I for the mt*COI*, n*EF*, and n*CAD* partitions, respectively. Data partitions were unlinked. Posterior probabilities were assessed with TRACER 1.5.0 (http://beast.bio.ed.ac.uk, accessed on 7 June 2023) to ensure that sampling had reached stationarity. The tree samples were summarized after the elimination of 25% of the tree samples. For the individual analyses, the trees are rooted using the following outgroups: *Ceutorhynchus erysimi* (Fabricius, 1787) (MG954909) and *Baetis tricaudatus* Dodds, 1923 (GU713864) for the mt*COI* gene, *Rhinusa pilosa* (Gyllenhal, 1838) (KJ620002) and *Delias rosenbergii* (van Vollenhoven, 1865) (AB899863) for the n*EF-1α* gene, and *Zacladus geranii* (Paykull, 1800) (HQ883772) and *Symbrenthia hippoclus* Cramer, 1782 (OK743939) for the n*CAD* gene. For the partitioned analysis, the tree was rooted with *Anteos maerula* (Fabricius, 1775) (mt*COI*, GU164131; n*EF-1α*, OK736658; n*CAD*, OK743412) and *Tribolium castaneum* (Herbst, 1797) (mt*COI*, ON482348; n*EF-1α*, NM_001114363; n*CAD*, EU677538).

The evolutionary relatedness and genealogy of the recorded mt*COI* genotypes were assessed by constructing a haplotype network for some of the selected taxonomic entities and groups. Population genetic specificity, intraspecific variations, and relationships resulting from underlying population dynamics, such as the persistence of ancestral haplotypes, are better visualized in reticulograms or networks than in evolutionary gene trees [27]. The networks were constructed using the software PopART version 1.7 [28] on the basis of inferred gene genealogies by performing median-joining (MJ) calculation while keeping the parameter e = 0 [29]. To study the correlations among the genotypes from their geographic origin, the networks were constructed for species with complex genealogies, such as *R. oxyacanthae* and *R. bavierai* Diotti, Caldara & Toševski, 2021, as well as, for two closely related taxonomic entities forming the *R. pulicarius* and *R. monzinii* Pesarini & Diotti, 2012 species groups.

## 3. Results

### 3.1. Morphological Study

**Figure 2:**
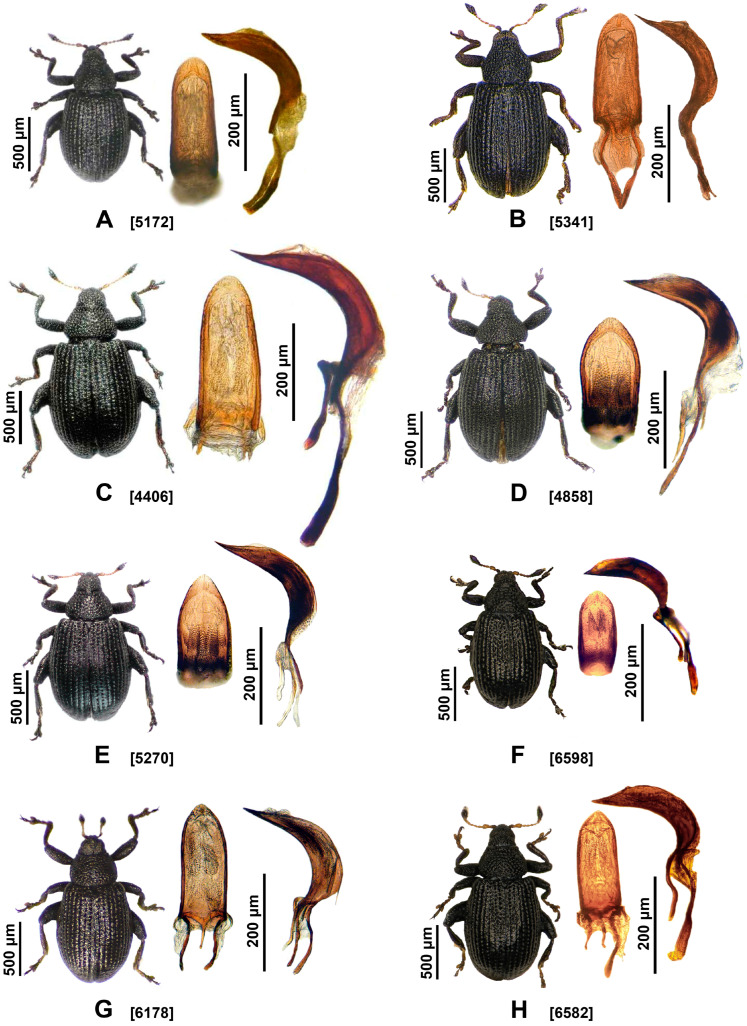
Species of the genus *Rhamphus* with penis morphology in dorsal and lateral views. The numbers in square brackets indicate the voucher codes of the specimens shown in Table S1: (**A**) *Rhamphus oxyacanthae* [5172]; (**B**) *R. hampsicora* [5341]; (**C**) *R. bavierai* [4406]; (**D**) *R. monzinii* [4858]; (**E**) *R. diottii* sp. nov., holotype [5270]; (**F**) *R. ibericus* sp. nov., holotype [6598]; (**G**) *R. cypricus* sp. nov., paratype [6178]; (**H**) *R. macedonicus* sp. nov., holotype [6582].

**Figure 3:**
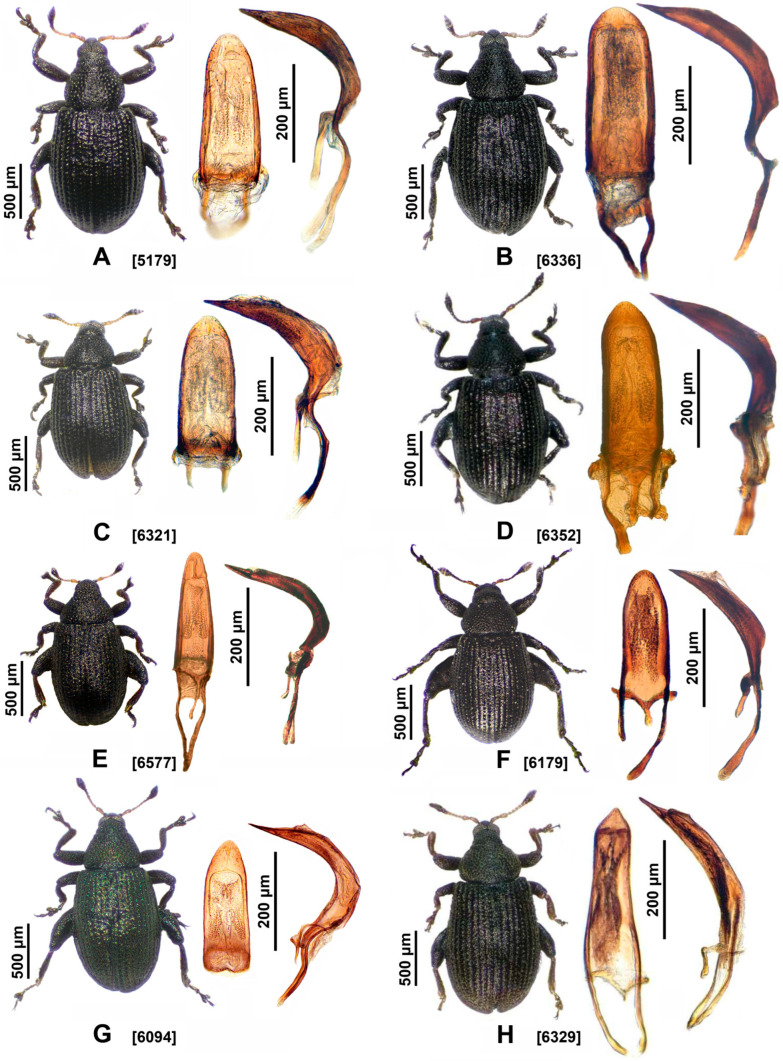
Species of the genus *Rhamphus* with penis morphology in dorsal and lateral views. The numbers in square brackets indicate the voucher codes of the specimens shown in Table S1: (**A**) *Rhamphus pulicarius* [5179]; (**B**) *R. pullus* [6336]; (**C**) *R. betulae* sp. nov., paratype [6321]; (**D**) *R. crypticus* sp. nov., paratype [6352]; (**E**) *R. cerdanicus* [6577]; (**F**) *R. loebli* [6178]; (**G**) *R. subaeneus* [6094]; (**H**) *R. hisamatsui* [6329].

In contrast to substantial genetic lineage differentiation, the specimens collected from different host plants are morphologically very similar, making it very difficult to differentiate them exclusively on the basis of their phenotypic characteristics, which strongly confirm the cryptic nature of these weevils (Figure 2 and Figure 3). The average body length, shape of the pronotum and elytra, length of the antennal segments and color, shape of the tibial uncus, and morphology of the penis are subtle morphological characteristics that rarely allow separation of the species. Additionally, because the genitalia of females do not differ from one another, accurate species-level identification requires molecular methods, and sequencing is the method of choice. Thus, we identified morphotypes on the basis of subtle morphological characteristics and their associations with different host plants. This allowed us to separate the collected *Rhamphus* specimens into eight morphotypic groups, the characteristics of which are reported in a dichotomous key with a synoptic key for the species when more than one is in a group (see Section 3.3).

### 3.2. Molecular Analysis

In total, molecular analysis was conducted on 326 *Rhamphus* specimens representing 16 entities. The final dataset comprises 326 mt*COI*, 229 n*EF-1α*, and 234 n*CAD* sequences.

**Mt*COI* gene**: The final alignment of the mt*COI* sequences contained 621 bp, with a total of 252 polymorphic nucleotides of which 224 were parsimony informative. The maximum in-group genetic distance was 18.8%, and the minimum in-group distance was 4.2% (uncorrected). Across the 326 sequenced specimens, a total of 140 different haplotypes were identified within the *Rhamphus* populations, with an average pairwise divergence across all the haplotypes of 11.8%, whereas the divergence across sequence pairs within different entities ranged from 0 to 1.8% (Table S8a).

**Figure 4:**
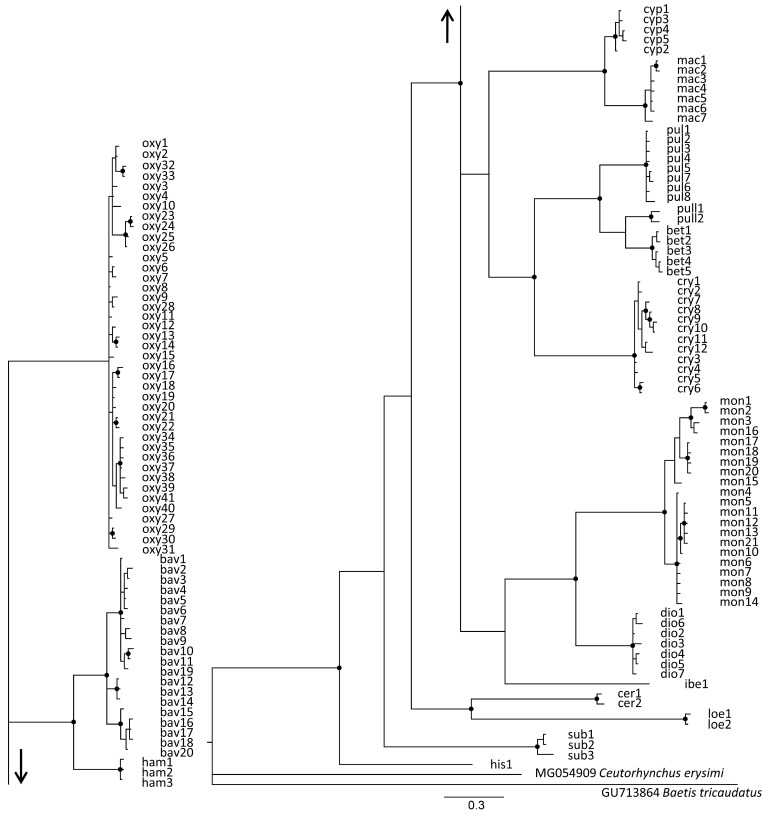
Bayesian phylogenetic tree inferred from 621 bp of the barcoding mitochondrial cytochrome oxidase subunit I (mt*COI*) gene sampled from different *Rhamphus* populations originating from the west Palearctic. Nodes with bootstrap support ≥ 90 are indicated by black circles. The arrows indicate connections between two parts of the unique phylogenetic tree. The haplotypes corresponding to different taxonomic entities are designated according to the following abbreviations: oxy—*Rhamphus oxyacanthae*; bav—*R*. *bavierai*; ham—*R. hampsicora*; cyp -*R. cypricus* sp. nov.; mac—*R. macedonicus* sp. nov.; pul—*R. pulicarius*; pull—*R. pullus*; bet—*R. betulae* sp. nov.; cry—*R. crypticus* sp. nov.; mon—*R. monzinii*; dio—*R. diottii* sp. nov.; ibe—*R. ibericus* sp. nov.; sub—*R. subaeneus*; cer—*R. cerdanicus*; loe—*R. loebli*; his—*R. hisamatsui*.

Bayesian phylogenetic analyses revealed substantial genetic divergence between 16 entities at the species level, all of which clustered as monophyletic groups with strong bootstrap support (94%) (Figure 4). All western Palearctic entities, including *R. pullus* from Japan, clustered together with 85% bootstrap support. In general, the phylogenetic tree topology was in accordance with the morphological findings, with the exception of species forming the *R. oxyacanthae* group, where *R. oxyacanthae* displays weak connections with *R. bavierai* and *R. hampsicora* Diotti, Caldara & Toševski, 2021, whereas the last two species show 100% bootstrap support. Interestingly, according to the mt*COI* tree topology, *R. cerdanicus* Tempère, 1982 and *R. loebli* Germann & Colonnelli, 2018 clustered together with 91% support, although this finding conflicts with the distinctive morphological separation between these two species and may represent the effect of long branch attraction [30].

**Figure 5:**
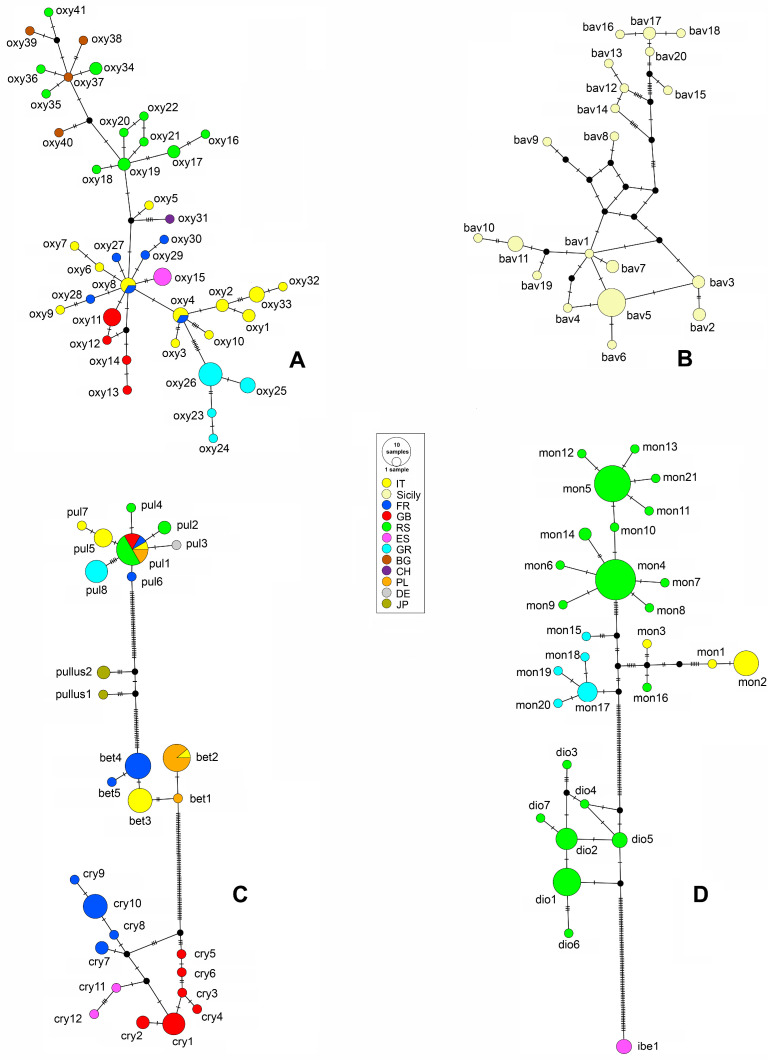
Median-joining network obtained from 621 bp of the barcoding mitochondrial cytochrome oxidase subunit I (mt*COI*) gene sequences of the *Rhamphus oxyacanthae* (**A**), *R. bavierai* (**B**), *Rhamphus pulicarius*-group (**C**), and *Rhamphus monzinii*-group (**D**). The circle sizes are proportional to the haplotype frequency. Each SNP mutation is represented by a hatch mark, whereas black vertices represent median vectors. The colors correspond to the geographical affiliation of the recorded haplotypes (rectangle box with two-letter codes for the country of origin). oxy—*Rhamphus oxyacanthae*; bav—*R. bavierai*; pul—*R. pulicarius*; pullus—*R. pullus*; bet—*R. betulae* sp. nov.; cry—*R. crypticus* sp. nov.; mon—*R. monzinii*; dio—*R. diottii* sp. nov.; ibe—*R. ibericus* sp. nov.

Across the studied area, a total of 41 mt*COI* haplotypes of *R. oxyacanthae* were recorded with an in-group genetic divergence of 1% (Table S8a). The median-joining networks contained only a single ambiguous connection among the haplotypes (Figure 5A). In contrast, the gene genealogy constructed for 20 haplotypes of *R. bavierai* exhibited complex mutual connections and a median-joining network containing multiple ambiguous connections among the haplotypes (Figure 5B). The average in-group mt*COI* divergence was 1.8% among the haplotypes limited exclusively to the isle of Sicily (Table S8a).

The median-joining network constructed for the *R. pulicarius* group showed strict separation of four morphologically closely related cryptic species, namely, *R. pulicarious*, *R. pullus*, *R. betulae* sp. nov., and *R. crypticus* sp. nov., with average intraspecific divergence of 0.4, 1.1, 0.4, and 0.7%, respectively (Table S8a). The network constructed with 27 haplotypes recorded within groups contained a single ambiguous connection within geographically distant haplotypes of *R. crypticus* sp. nov. (Figure 5C). Two haplotypes of *R. pullus* from Japan were positioned between *R. pulicarius* and *R. betulae* sp. nov. from Europe.

As shown in Figure 5D, the haplotype network of the *R. monzinii* group clearly separated the species *R. monzinii*, *R. diottii* sp. nov., and *R. ibericus* sp. nov., with average intraspecific divergence values of 1.8, 0.6, and 0%, respectively (Table S8a). Three ambiguous connections were recorded among eight haplotypes associated with *R. diottii* sp. nov.

**n*EF-1α* gene**: The final alignment of the n*EF-1α* sequences contained 489 bp, with a total of 124 polymorphic nucleotides, 83 of which were parsimony informative. The maximum in-group genetic distance was 12.2%, whereas the minimum in-group genetic distance was 0.6% (uncorrected). Across the 229 sequenced specimens, a total of 66 different haplotypes were identified within the *Rhamphus* populations, with an average pairwise divergence across all haplotypes of 4.6%, whereas the divergence across sequence pairs within different entities ranged from 0 to 0.7% (Table S8b).

**Figure 6:**
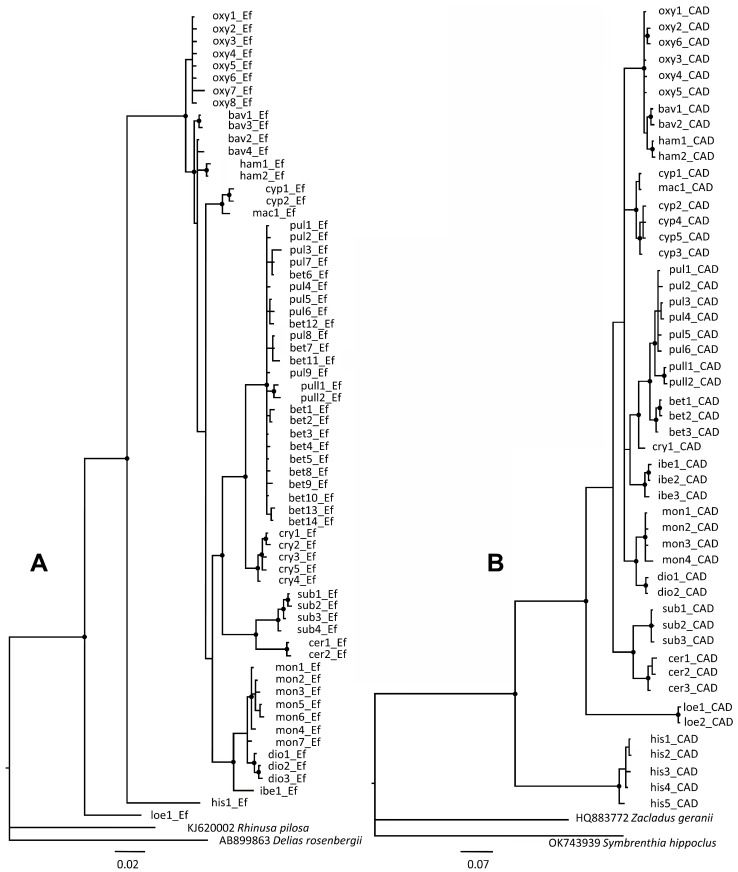
Bayesian phylogenetic trees inferred from 489 bp of the nuclear *EF-1α* gene (**A**) and 459 bp of the *CAD* gene (**B**) sampled from different *Rhamphus* populations originating from the western Palearctic. Nodes with bootstrap support ≥90 are indicated by black circles. The haplotypes corresponding to different taxonomic entities are designated according to the abbreviations presented below: oxy—*Rhamphus oxyacanthae*; bav—R. *bavierai*; ham—*R. hampsicora*; cyp—*R. cypricus* sp. nov.; mac—*R. macedonicus* sp. nov.; pul—*R. pulicarius*; pull—*R. pullus*; bet—*R. betulae* sp. nov.; cry—*R. crypticus* sp. nov.; mon—*R. monzinii*; dio—*R. diottii* sp. nov.; ibe—*R. ibericus* sp. nov.; sub—*R. subaeneus*; cer—*R. cerdanicus*; loe—*R. loebli*; his—*R. hisamatsui*.

Bayesian phylogenetic analyses according to n*EF-1α* supported the monophyly of 16 taxonomic entities with bootstrap support of 100% (Figure 6A). The phylogenetic tree topology was in accord with the morphologically defined groups, with few conflicts with respect to the tree topology obtained with the mt*COI* gene. Species from the *R. oxyacanthae* group were closely related in terms of their morphological similarity and clustered together with 100% bootstrap support. However, the haplotypes of *R. pulicarius* and *R. betulae* sp. nov. were not clearly separated according to the corresponding species defined by mt*COI*, exhibiting low bootstrap support for the n*EF-1α* gene, with the exception of *R. crypticus* and *R. pullus* from Japan. Four haplotypes, representing *R. betulae* sp. nov. (bet6_Ef, bet7_Ef, bet8_Ef and bet12_Ef), clustered together with *R. pulicarius* haplotypes (Figure 6A), potentially suggesting introgression events. In addition, two species even share identical haplotypes (pul8_Ef≡bet7_Ef, pul7_Ef≡bet6_Ef, and pul1_Ef≡bet5_Ef). However, within the *R. pulicarius* group, evidence of introgression within the n*CAD* gene was not recorded in 48 sequenced specimens of *R. pulicarius* and *R. betulae* sp. nov. originating from England, France, Italy, and Serbia, including specimens from Poland collected in sympatry. Three species from the *R. monzinii* group clustered together with 100% bootstrap support (Figure 6A).

**n*CAD* gene**: The final alignment of the n*CAD* sequences contained 459 bp, with a total of 141 polymorphic nucleotides, 131 of which were parsimony informative. The maximum in-group genetic distance was 19.8%, whereas the minimum in-group distance was 0.5% (uncorrected). Across 234 sequenced specimens, a total of 50 different haplotypes were identified with an average pairwise divergence across all the haplotypes of 8.3%, whereas the divergence across sequence pairs within different entities ranged from 0 to 0.9% (Table S8c).

Bayesian phylogenetic analyses supported the monophyly of all 16 taxonomic entities, with bootstrap support of 100% (Figure 6B). In general, the phylogenetic tree topology follows morphologically defined groups, with a few inconsistencies with respect to the tree topology obtained with mt*COI* and n*EF-1α*. The monophyly of the *R. oxyacanthae* group was confirmed with 100% bootstrap support, and this finding was consistent with their morphological similarity. The four species from the *R. pulicarius* group clustered together with 100% bootstrap support and showed no signs of introgression. However, the same haplotypes cyp1 and mac1 were recorded in two closely related species, *R. cypricus* sp. nov. and *R. macedonicus* sp. nov. *Rhamphus subaeneus* and *R. cerdanicus* clustered together with 100% bootstrap. This is consistent with findings recorded for the n*EF-1α* gene, confirming their close phylogenetic relationship. The recorded divergence of the n*CAD* gene among *R. loebli* and other *Rhamphus* species from the western Palearctic was extraordinarily high, ranging from 13.2 to 15%. Strong monophyly between species from the *R. monzinii* group was not confirmed based on n*CAD* topology, showing weak support between *R. ibericus* sp. nov. and the other two species from the *R. monzinii* group.

**Figure 7:**
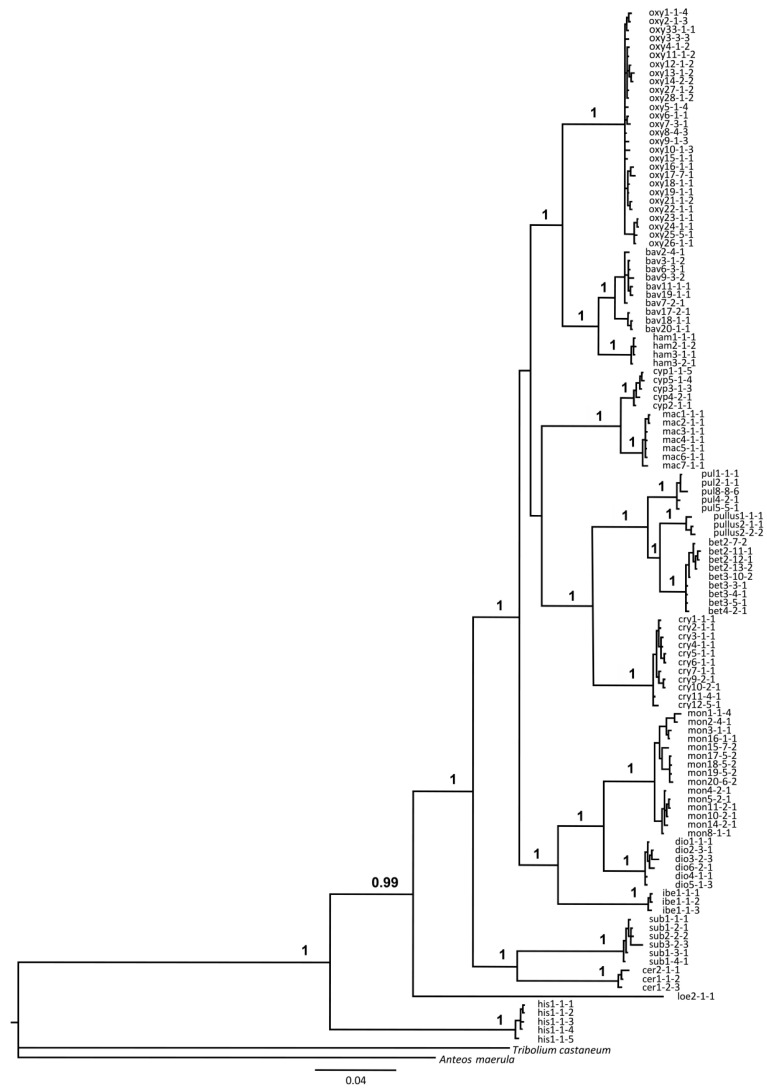
Partition-based multigene estimation in MrBayes based on the mt*COI*, n*EF-1α*, and n*CAD* genes. Posterior probabilities with values ≥ 0.95 are marked above the branches. Different taxonomic entities are designated according to their abbreviations: oxy—*Rhamphus oxyacanthae*; bav—R. *bavierai*; ham—*R. hampsicora*; cyp -*R. cypricus* sp. nov.; mac—*R. macedonicus* sp. nov.; pul—*R. pulicarius*; pull—*R. pullus*; bet—*R. betulae* sp. nov.; cry—*R. crypticus* sp. nov.; mon—*R. monzinii*; dio—*R. diottii* sp. nov.; ibe—*R. ibericus* sp. nov.; sub—*R. subaeneus*; cer—*R. cerdanicus*; loe—*R. loebli*; his—*R. hisamatsui*. Numbers after the abbreviations correspond to haplotype numbers on mt*COI*, n*EF-1α*, and n*CAD* genes, respectively.

***Partitioned multigene analysis***: Bayesian phylogenetic analysis based on the partitioned dataset adequately resolved relationships between groups and the species within, which is in agreement with phylogenetic analyses based on individual gene markers. Most species groups were recovered as monophyletic, while *R. oxyacanthe* and *R. pulicarius* groups recovered as sister to the remaining groups. However, their node and the node delimiting the *R. cypricus* and *R. pulicarius* groups are the only ones with weaker support in the otherwise well-supported phylogeny (posterior probabilities above 0.99; Figure 7).

### 3.3. Treatment of the Species

***Rhamphus* [Clairville]**
*Rhamphus* [Clairville], 1798: pl. xii type species *Rhamphus flavicornis* [Clairville], 1798 nomen oblitum (= *Curculio oxyacanthae* Marsham, 1802 nomen protectum) [31], Morimoto, 1984: 20 [32]. Kojima & Morimoto, 1996: 112, 114 [33]. Caldara et al., 2022: 375 [14]. Alonso-Zarazaga et al., 2023: 213 [1]. Caldara & Tedeschi, 2025: 3 [7].
*Rhamphonyx* Voss, 1964: 592 type species *Rhamphonyx tarsalis* Voss, 1964 [4]. Colonnelli, 2009: 230 [34].
*Rhamphus* subgen. *Nanorhamphus* Korotyaev, 1984: 352 type species *Rhamphus emeljanovi* Korotyaev, 1984 [35]. Kojima & Morimoto, 1996: 114 [33]. Colonnelli, 2009: 230 [34].
*Rhamphus* subgen. *Trichorhamphus* Korotyaev, 1984: 351 type species *Rhamphus hisamatsui* Chûjô & Morimoto, 1960 [35]. Kojima & Morimoto, 1996: 114 [33]. Colonnelli, 2009: 230 [34].

***Diagnosis***. Head strongly bent between eyes and thence forming flat surface to apex of rostrum. Eyes oblong–oval, approximate dorsally. Antennae inserted in forehead, not geniculate, with oval scape as long as or slightly longer than first segment of funicle. Prosternum with coxae separated from each other but usually concealed by retracted rostrum. Mesosternal process and metasternum flat, former about as wide as abdominal process between hind coxae. Venter with ventrites 2–4 straight in posterior margin. Femora unarmed, hind femora swollen. Pro- and mesotibiae are each armed with hook-shaped uncus little behind apex dorsally. Metatibiae simple, unarmed at apex.

***Biological notes***. The species belonging to the genus *Rhamphus* are leaf-miners as well as the other Rhamphini. The adults appear in early spring and feed, usually on the inner side of leaves, causing a large number of tiny holes. Copulation occurs from spring to middle summer. The female oviposits one egg between the upper and lower epidermis. Larvae are flattened in shape, adapted for leaf-miner behavior. Mines are irregularly round, pear-shaped, and contain only one larva. The last instar larvae stay inside the mine and overwinter in the leaves fallen on the soil surface. In the early spring, larvae pupate inside the mine. The host plants of *Rhamphus* species belong to several families: in the Palearctic region, Rosaceae (mainly *Prunus*, *Pyrus*, and *Crataegus*), Salicaceae (*Salix*, *Populus*), Betulaceae (*Betula*), and Cistaceae (*Halimium*), and in the Afrotropical, Oriental, and Australian regions, Fabaceae (Caesalpinioideae). The species are usually monophagous and do not feed on plants belonging to different families.

***Distribution***. This genus is known to occur in the Palearctic, Afrotropical, Oriental, and Australian regions.

***Remarks and comparative notes***. The name of the author of the genus Clairville between brackets is necessary because the paper was originally published anonymously. This genus is easily distinguishable from the other genera of the tribe Rhamphini mainly by the forehead strongly bent between eyes and prominent anteriorly; therefore, rostrum always retracted between forecoxae, antennae inserted in lateral surfaces of prominence between eyes, not geniculate, scrobes absent.

**(1)**  ***Rhamphus oxyacanthae* group**

**Figure 8:**
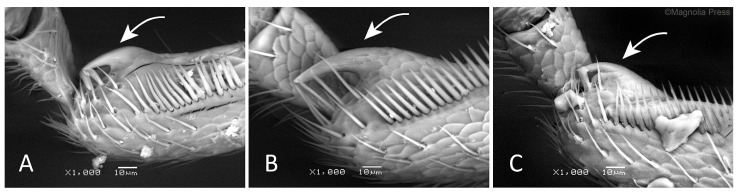
Distal end of mesotibiae with uncus (arrow): (**A**) *Rhamphus oxyacanthae*. (**B**) *R. pulicarius*. (**C**) *R. monzinii* [11]. Reproduced with permission from the copyright holder.

**a. *Rhamphus oxyacanthae* (Marsham, 1802)** (Figure 2A and Figure 8A)
*Curculio oxyacanthae* Marsham, 1802: 263 (nomen protectum). Caldara et al., 2022: 373 [14].
*Rhamphus oxyacanthae* (Marsham). Hering, 1921: 126 [36].
*Rhamphus flavicornis* [Clairville], 1798: 104 (nomen oblitum). Caldara et al. 2022: 375 [14].

***Type locality***. Lakenheath Station (England, Suffolk).

***Type series***. A neotype was designated by Caldara [14], due to the missing of type specimens, and deposited at the BMNH. Its online acc. number is MZ404333 (NCBI database).

***Synonyms***. Due to the missing of type specimens, Caldara [14] also designated a neotype of *Rhamphus flavicornis* (deposited at the NHMB), which was established as nomen oblitum, and confirmed its synonymy with *R. oxyacanthae*. Its online acc. number is MZ404334 (NCBI database).

***Non-type specimens***. About 200 specimens, in addition to 65 sequenced specimens, from the following countries: Bulgaria, England, France, Greece, Italy, Serbia, and Switzerland (Table S1).

***Diagnostic redescription***. Length 1.3–1.7 mm. Integument black, tarsi dark brown, antennae yellow except for darker scape and blackish club. Vertex of head moderately convex. Tubercles of rostrum at base of antennae very distinct and well separated from each other. Sculpture of rostrum in dorsal view with one lateral row of punctures. Antennal scape is darker than segments of funicle, 2.4–2.6× longer than wide, and 1.2–1.3× longer than first segment of funicle. Funicle with first segment asymmetrical and irregularly clavate at basal half, 2.0–2.2× longer than wide and 1.3–1.4× longer than second, which is 1.9–2.1× longer than wide; third and fourth segments 1.9–2.0× longer than wide; fifth-seventh segments transverse. Pronotum moderately conical, moderately transverse (Pw/Pl 1.60–1.70), widest between basal and middle third, with slightly curved sides, punctures moderately dense, irregularly arranged, with intervals between punctures partly wider than puncture diameter. Elytral shape suboval, usually slightly elongated (El/Ew 1.30–1.40), wider at base than pronotum, widest at apical third (Ew/Pw 1.18–1.23), and slightly convex. Elytral interstriae slightly convex on disc. Uncus of pro- and metatibiae large, measuring 3.0× longer than wide. First tarsomere 2.2–2.5× longer than wide, second tarsomere 1.2–1.4× longer than wide; onychium 3.8–4.0× longer than wide. Metafemora moderately globose (l/w 3.5). Body of penis in dorsal view nearly similar in width from base to apex, with rectilinear subparallel sides.

***Host plants***. This species is oligophagous in the Rosaceae. We sequenced specimens collected on *Crataegus monogyna*, *C. nigra*, *Cydonia oblonga*, *Malus domestica*, *Prunus cerasifera*, and *Pyrus spinosa*. It was also collected on *Mespilus germanica*, *Prunus avium*, *P. cerasus*, *P. domestica*, and *Pyrus communis*. The larvae are confirmed by sequencing in the mines collected from *Crataegus monogyna*, *C. nigra*, and *Pyrus spinosa*.

***Distribution***. This species was previously cited in almost all of Europe [1]. However, as for *R. pulicarius*, after our discovery of a new cryptic species, its distribution could be reviewed, although probably not at a surprising level. Currently, the presence of this species, based on our molecular study, is confirmed in countries of Central, Western, and Eastern Europe. It is apparently lacking in isles like Sicily, Sardinia, and Cyprus, where it seems to be replaced by vicariant cryptic species.

***Remarks and comparative notes***. It is very difficult to distinguish *R. oxyacanthae* from the other species of its group. Currently, each of these cryptic species appears to have a different distribution, aside from very few distinctive subtle morphological characters (see remarks of every species).

**b. *Rhamphus bavierai* Diotti, Caldara & Toševski, 2021** (Figure 2C)
*Rhamphus bavierai* Diotti, Caldara & Toševski, 2021: 115 [11].

***Type locality***. Piano Zucchi (Palermo province, Sicily).

***Type series***. About 100 specimens from several localities in all of Sicily, 35 of which were sequenced [11].

***Non-type specimens***. None.

***Diagnostic redescription***. Length 1.4–1.8 mm. As *R. oxyacanthae*, except vertex of head more convex, tubercles of rostrum at base of antennae less distinct and poorly separated from each other, metafemora more globose, first tarsomere of metatarsi longer (l/w 3.0–3.3), body of penis slightly longer.

***Host plants***. This species was collected on *Crataegus* sp. at the type locality and on *Crataegus monogyna* at Bosco di Ficuzza by L. Diotti, on *Crataegus laciniata*, a Sicilian endemism, by C. Baviera in the Nebrodi Mountains. In the type locality, C. Baviera also collected *R. bavierai* on *Pyrus* sp. located very near plants of *Crataegus* sp.

***Distribution***. Distributed throughout Sicily.

***Remarks and comparative notes***. This taxon is very closely related to *R. oxyacanthae* and *R. hampsicora*. It differs from both species by the longer first tarsomere of the metatarsi and the body of the penis. Moreover, it differs from *R. oxyacanthae* in terms of the characters reported in the diagnosis. On the contrary, it shares all these characters with *R. hampsicora*, from which, apart from the length of tarsi, it can be separated by shape of pronotum, which is less conical and less transverse, widest more forward (between middle and basal third), and with less curvilinear sides; body of penis with rectilinear sides.

**c. *Rhamphus hampsicora* Diotti, Caldara & Toševski, 2021** (Figure 2B)
*Rhamphus hampsicora* Diotti, Caldara & Toševski, 2021: 117 [11].

***Type locality***. Siniscola, Monte Albo (Nuoro province, Sardinia).

***Type series***. Described on about 50 specimens from central and northern Sardinia [11], four of which were sequenced.

***Non-type specimens***. None.

***Diagnostic redescription***. Length 1.5–1.8 mm. As *R. oxyacanthae*, except vertex of head more convex, tubercles of rostrum at base of antennae less distinct and poorly separated from each other, and metafemora more globose.

***Host plants***. This species was consistently collected in Sardinia by L. Diotti by beating *Prunus* sp. at the type locality, Monte Albo, and also at Burcei.

***Distribution***. Sardinia.

***Remarks and comparative notes***. This taxon has many characters in common with *R. bavierai* that allow it to be distinguished from *R. oxyacanthae*, such as the vertex of head being more convex, tubercles of rostrum at base of antennae being less distinct and poorly separated from each other, and metafemora being more globose. *Rhamphus hampiscora* differs from *R. bavierai* only by shorter tarsi, especially first tarsomere of metatarsi; shape of pronotum, which is more conical and more transverse, widest at its base, and with more curvilinear sides; shape of penis with slightly curved sides.

**(2)**  ***Rhamphus cypricus* group**

**a. *Rhamphus cypricus* Toševski & Caldara sp. nov.** (Figure 2G)
LSID urn:lsid:zoobank.org:act:54F1C08D-2B97-4108-90FA-236008A59D8C

***Type locality***. Lefkara (Larnaca, Cyprus).

***Type series***. Holotype, male: CY - Cipro Parsata 07.IV.2024 L. Forbicioni *leg*/Beating *Crataegus* sp. 34.8281745 N 33.2648008 E; (sequenced, BMNH). Paratypes: same data as holotype (7 sequenced: 4 ITCB, 3, RCCM; 52 LFCP); “CY - Cipro, Lefkara (or Leukara), 12.IV.2024, L. Forbicioni *leg.*/Beating *Crataegus* sp. 34.8692177 N 33.2839350 E” (2 sequenced: 1 ITCB, 1 RCCM); “CY - Cipro Sarama, 05.IV.2024, L. Forbicioni *leg*/Beating *Crataegus* sp. 34.9564348 N 32.5213484 E” (36, LFCP); “Droushia-Ineia (Paphos, Chypre) ± 600 m, 12.04.2005, G. et H. Alziar lgt. sur *Crataegus* fleuri. Collection G. Alziar (2 sequenced, ITCB; 4, GACC); “Droushia (Paphos, Chypre), 500–600 m, 13.IV.2005/G. Alziar leg, sur *Crataegus*, Collection G. Alziar” (1, GACC); “Droushia (Paphos, Chypre), du village en descendant vers le nord/500–600 m, 17.IV.2010/sur *Crataegus aronia*, G. Alziar leg./*Rhamphus oxyacanthae*, G. Alziar det. 2010” (4, MHNN); “Polemi (Paphos, Chypre), 31.III.2007; 470 m, sur *Crataegus aronia*/G. Alziar leg, *Rhamphus oxyacanthae*, Collection G. Alziar (2, MHNN); piste vers “Plevra tou Liondakiou” ±500 m; sur *Crataegus azarolus*/Inia (Paphos, Chypre), 25-VI-95, G. Alziar leg./Collection G. Alziar” (1, MHNN); “de Vassilia au mont Kornos (Kerynia, Chypre), 8-IV-2005/sur *Crataegus* fleuri, G. Alziar leg./Collection G. Alziar” (1, MHNN); “Cyprus, Dhiarizos river: Skidas, 34°48′15.60” N, 32°42′37.05” E, 264 m, 13.3.2024, *Crataegus azarolus*, l. Stüben (28)” (16, PSCC); “Cyprus, 2 km SW Agia Varvara, 34°58′58.31” N 33°21′14.12” E, 342 m, 20.3.2024, *Crataegus*, leg. Stüben (37)” (9, PSCC); “Cyprus, Paphos p., Drouseia env., 541 m. 23.03.2019, lgt. F. Pavel (1 sequenced, JKCH); “Cyprus: surroundings of Drouseia, m 500–600, 18.IV.2010, C. Giusto” (4, RCCM).

***Non-type specimens***. None.

***Diagnostic description***. Length 1.4–1.7 mm. As *R. oxyacanthae*, except vertex of head more convex, tubercles of rostrum at base of antennae less distinct and poorly separated from each other, antennal scape shorter than first segment of funicle, which is smaller, only as large as scape, pronotum less transverse (Pw/Pl 1.50–1.60) with sides less curved, with denser and more regular punctures, with intervals between punctures usually narrower than puncture diameter; body of penis shorter, with parallel sides.

***Etymology***. The name of this species refers to the island where it was collected.

***Host plants***. This species was usually collected on *Crataegus azarolus*, although several specimens were collected on *Crataegus* sp. It is noteworthy that only two species of *Crataegus* are reported from Cyprus: *C. azarolus* with its var. *aronia* and *C. monogyna*.

***Distribution***. Currently known only from the isle of Cyprus.

***Remarks and comparative notes***. Very closely related to *R. macedonicus*, from which it differs by smaller first funicular segment, which is as wide as the scape, and body shape, which is often slightly larger (1.4–1.7 mm vs. 1.2–1.5 mm).

**b. *Rhamphus macedonicus* Toševski & Caldara sp. nov.** (Figure 2H)
LSID urn:lsid:zoobank.org:act:8A053162-AE6E-435F-A325-980CDD7EDB87

***Type locality***. Arethousa (Volvi, Central Macedonia, Greece).

***Type series***. Holotype, male: “Greece, Macedonia, Arethousa, 23.06.2023, N40 44.134 E23 36.193, 374 m. lgt. Toševski” (sequenced, BMNH). Paratypes: same data as holotype (2 sequenced, ITCB, RCCM); same locality as holotype except 13.06.2024, lgt. Jović (12 sequenced: 9 ITCB, 3 RCCM).

***Non-type specimens***. None.

***Diagnostic description***. Length 1.2–1.5 mm. As *R. oxyacanthae*, except tubercles of rostrum at base of antennae more distinct, pronotum with punctures denser, more regularly arranged, with intervals between punctures usually narrower than puncture diameter.

***Etymology***. The name of this species refers to the country where it was collected.

***Host plants***. All specimens were collected by beating branches of *Crataegus* sp. closely related to *C. monogyna*.

***Distribution***. Greece (Central Macedonia). We expect that further study will result in more cryptic species from this group in the Middle and Central Asia region.

***Remarks and comparative notes***. See comparative notes of *R. cypricus*.

**(3)**  ***Rhamphus monzinii* group**

**a. *Rhamphus monzinii* Pesarini & Diotti, 2012** (Figure 2D and Figure 8C)
*Rhamphus monzinii* Pesarini & Diotti, 2012: 112 [9]. Diotti et al., 2021: 121 [11].

***Type locality***. Giaglione (Piedmont, Northern Italy).

***Type series***. This species was described on seven specimens (all collected on the same date at the type locality), which we examined by sequencing one of them.

***Non-type specimens***. (all sequenced)**.** “Italy, Liguria, (GE), Piani di Creto, 23.06.2018, lgt. L. Diotti” (10, ITCB); “Serbia, Krnjevo, Radovanovic, 13.10.2022, N44 25.985 E21 1.927, ex larva L3, lgt. Toševski” (4, ITCB); “Serbia, Mt. Zlatibor, 9.07.2017, N43 47.310 E19 43.721, 662 m, lgt. Toševski” (1, ITCB); “Serbia, Pirot, Staničenje, 20.05.2018, N43 13.020 E22 30.556, 403 m, lgt. Toševski” (1, ITCB); “Serbia, Brusnik, Negotin, N44 6.489 E22 24.115, 322 m, 21.05.2018, lgt. Toševski” (3, ITCB); “Serbia, Vlasina, Božićki Kanal, N42 40.997 E22 21.888, 1289 m, 21.06.2018, lgt. Toševski” (1, ITCB); “Serbia, Velika Vrbica, Kladovo, 17.11.2023 N44 36.072 E22 41.557, 59 m., lgt. Toševski” (1, ITCB); “Greece, Mt. Taygetos 10.07.2018, N37 04.155 E22 15.882, lgt. Toševski” (1, ITCB); “Greece, Arethousa, 23.06.2023, Macedonia, N40 44.299 E23 34.854, lgt. Toševski” (8, ITCB).

***Diagnostic description***. Length 1.2–1.5 mm. Vertex of head moderately convex. Tubercles of rostrum at base of antennae very distinct and well separated from each other. Rostrum in dorsal view with two to three irregular rows of punctures. Antennal scape as reddish as segments of the funicle, 2.1–2.3× longer than wide, nearly as long as first segment of funicle; this segment symmetrical and regularly clavate, 1.7–1.8× longer than wide and 1.3–1.4× longer than the second, which is 1.8–2.0× longer than wide; third segment 1.7–1.9× longer than wide; fourth segment 1.5–1.6× longer than wide; fifth-seventh segments transverse. Pronotum moderately conical, moderately transverse (Pw/Pl 1.60–1.70), widest between basal and middle third, with slightly curved sides. Elytral shape elliptical (El/Ew 1.25–1.30), at base only slightly wider than pronotum, widest at middle (Ew/Pw 1.15–1.20), distinctly convex. Elytral interstriae moderately convex on disc. Uncus of pro- and metatibiae small, only 1.5× longer than wide. Metatarsi with first tarsomere 1.5–1.8× longer than wide, second tarsomere 1.2–1.4× longer than wide; onychium 3.0–3.3× longer than wide. Metafemora distinctly globose (l/w 2.5). Body of penis in dorsal view short, slightly narrowing from base to apex, with slightly concave sides in basal two-thirds.

***Host plants***. Diotti [11] found a large series of specimens of this species at Montoggio (Piani di Creto, Liguria, Northern Italy) only on *Prunus spinosa*, growing near plants of *Crataegus monogyna*, on which, on the contrary, only specimens of *R. oxyacanthae* and *R. subaeneus* were collected.

***Distribution***. This species, which was previously known only from Northern Italy, actually also lives in the Balkans (Serbia, Greece), where it is the most common *Rhamphus* species associated mainly with *Prunus cerasifera*.

***Remarks and comparative notes***. This species seems closely related to *R. diottii* by the size of the uncus of the pro- and metatibiae, which is smaller, and the size of the penis, whose body is distinctly shorter than in the other species. Reddish color of scape, first funicular segment being more globose; shape of the elytra being elliptical with less prominent humeri and only slightly wider than pronotum, which is widest more towards base and more globose, allow these two species to be separated from *R. oxyacanthae* and related species. All these characters (except reddish color of scape) allow us to distinguish *R. monzinii* and *R. diottii* from *R. pulicarius* and related species.

**b. *Rhamphus diottii* Toševski & Caldara sp. nov.** (Figure 2E)
LSID urn:lsid:zoobank.org:act:AFF3D3BD-6809-4991-AE34-7C22B806A2E4

***Type locality***. Slankamen Vinogradi (Vojvodina, Serbia).

***Type series***. Holotype, male: “Serbia, Slankamen Vinogradi, 29.05.2009, N45 9.715 E20 11.750, 224 m., lgt. Toševski” (sequenced, BMNH). Paratypes (all sequenced): same data as the holotype (4, ITCB; 3, RCCM); “Serbia, Sl. Vinogradi, N45 9.610 E20 11.903, 15.06.2021, lgt. Toševski” (5, ITCB; 2, RCCM); “N. Serbia, Slankamen Vinogradi, 10.06.2017, N45 9.715 E20 11.750, 224 m., lgt. Toševski” (5, ITCB); “Serbia, Slankamen Vinogradi, 29.09.2022, (L3 larva) N45 9.715 E20 11.750, 224 m, lgt. Toševski” (1, ITCB); “Serbia, Backa, Ada, 24.10.2022 N45 47.786 E20 05.647 (L3 larva), lgt. Toševski” (2, ITCB); “Serbia, Dobanovci, 4.11.2022, N44 51.034 E20 11.132, lgt. Toševski (2, L3 larve, ITCB).

***Non-type specimens***. None.

***Diagnostic description***. As *R. monzinii*, except slightly larger in size (length 1.4–1.7 mm), second segment of funicle longer (l/w 2.0–2.2), uncus slightly larger, and body of penis with sides slightly convergent from base and, in lateral view, with longer apex.

***Etymology***. This species was named in honor of our friend and colleague, Luciano Diotti, who helped us in this study and recently passed away.

***Host plants***. The host plant is *Prunus spinosa*, confirmed at three locations in Serbia as well as by molecular analysis of larvae collected from mines of this plant. At two locations, *R. diottii* sp. nov. was recorded in syntopy with *R. monzinii*, which mainly develops on *Prunus cerasifera* and occasionally on *Prunus spinosa*.

***Distribution***. Serbia.

***Remarks and comparative notes***. See the above description and the remarks of *R. monzinii*.

**c. *Rhamphus ibericus* Toševski & Caldara sp. nov.** (Figure 2F)
LSID urn:lsid:zoobank.org:act:C32079EE-7E69-42EB-AB30-847265D913F3

***Type locality***. Elburgo-Burgelu (País Vasco, Araba/Álava, Spain).

***Type specimens***. Holotype, male: “Spain, País Vasco, Araba/Álava, Elburgo-Burgelu, 556 m, N42.812 W2.633, 15.06.2023, lgt. Iñigo Ugarte San Vicente/on *Prunus spinosa* in oak forest (*Q. robur* L.), thinned with gall oaks (*Q. faginea* Lam.), *Crataegus monogyna* Jacq., etc.” (sequenced, MCNM). Paratypes: as the holotype (2 sequenced: 1 ITCB, 1 RCCM; 3, IUCA).

***Non-type specimens***. None.

***Diagnostic description***. Length 1.4–1.6 mm. As *R. monzinii*, except the second segment of the funicle is slightly longer (l/w 1.9–2.1); elytra narrower (El/Ew 1.30–1.35), with sides less rounded; body of penis with parallel sides is not enlarged in apical third.

***Etymology***. The name of this species refers to the geographic term for where it was collected.

***Host plants***. The specimens of the type series were collected on *Prunus spinosa* L. (det. I. Ugarte San Vicente).

***Distribution***. Northern Spain.

***Remarks and comparative notes***. See the above description for the differences from *R. monzinii*. It differs from the very closely related *R. diottii* by shorter second segment of antennal funicle, which is only slightly longer than wide, slightly longer elytra, and parallel-sided body of penis.

**(4)**  ***Rhamphus subaeneus* group**

**a. *Rhamphus subaeneus* Illiger, 1808** (Figure 3G)
*Rhamphus subaeneus* Illiger, 1808: 324. Hustache, 1931: 1118 [37]. Caldara et al. 2022: 373 [14].
*Rhamphus aeneus* Boheman, 1833: 310 [38]. Alonso-Zarazaga et al., 2023: 213 [1].
*Rhamphus aeneus* var. *pannonicus* Csiki, 1905: 582 [39]. Alonso-Zarazaga et al., 2023: 213 [1].

***Type locality***. Portugal, without more detailed indications.

***Type series***. Recently, Caldara [14] designated a lectotype based on a syntype found at the ZMNB.

***Synonyms***. *Rhamphus aeneus* was described on specimens from southern France, and *R. aeneus* var. *pannonicus* was described on specimens collected in Hungary. After their descriptions, both taxa were always appropriately considered synonyms for *R. subaeneus*.

***Non-type specimens***. “Spain, San Vicente de Arana, La Dehesa Álava, 7-8.07.2018” (4 sequenced: 2 ITCB, 2 IUCA); Spain, País Vasco, provincia de Araba/Álava, Subijana de Álava, 518 m, 25-VI-2019, lgt. Iñigo Ugarte San Vicente” (1 sequenced, ITCB); “Czechia, Bohemia Centr., Chramosty, Brdce hill, N49°40′12”, E14°19′57”, 415 m., 21.05.2020, Lgt J. Kratky” (1 sequenced, JKCH); “Czechia, Bohemia Centr., Chramosty (6352), 24.6.2001, lgt A. Trmal” (1, JKCH); “CZ – Bohemia centr., Sedlčany (6352), 16.6.2002, lgt A. Trmal” (1, JKCH); “F-H. Pyrénées, Gavarnie, Vallée d’Ossoue, 42°44′34.8” N 00°02′19” W, Lgt J. Kratky 18.7.2021” (1. JKCH); “Spain, Madrid prov., SW La Serna d.Monte, 41°01′27.2” N 3°38′00.3” W, Lgt J. Kratky 28.5.2019” (1, JKCH); “Italy, Liguria, (GE), Piani di Creto, 23.06.2018, lgt. L. Diotti” (1, CBCC).

***Diagnostic redescription***. Length: 1.5–2.0 mm. Integument of body with slightly bronze reflection; legs with dark brown tibiae and tarsi; antennae yellowish except for darker scape and club. Vertex of head convex. Rostrum with upper surface with 2–3 confused rows of punctures only at sides, with two small tubercles at antennal insertion. Antennae with clavate scape, 2.3–2.5× longer than wide and slightly shorter than first funicular segment, this segment clavate, 1.9–2.1× longer than wide, as robust as scape, and distinctly more robust and 1.5–1.6× longer than second segment, which is 1.8–1.9× longer than wide; third and fourth segments 1.3–1.4× longer than wide, fifth-seventh segment transverse. Pronotum conical, transverse (Pw/Pl 1.65–1.75), widest at base, with curvilinear sides, with robust and thick punctures, with intervals between punctures narrow and rugulose. Elytra suboval, distinctly longer than wide (El/Ew 1.30–1.40), at base wider than pronotum, widest at apical third (Ew/Pw 1.15–1.18), weakly convex; striae clearly distinct, with thick punctures; interstriae slightly convex and distinctly rugulose. Metafemora is distinctly globose (l/w 2.5). Pro- and mesotibiae with robust uncus (l/w 2.5). First tarsomere is 2.2–2.4× longer than wide, second tarsomere 1.2–1.3× longer than wide; onychium 3.8–4.0× longer than wide. Body of penis with slightly arcuate sides, gradually narrowed from base to apex and slightly emarginate at its base.

***Host plants***. This species was observed feeding on various species of *Crataegus* (*C. azarolus*, *C. laevigata*, *C. monogyna*), *Prunus spinosa*, and *Pyrus communis* [40].

***Distribution***. This species is widely distributed throughout all of Europe and Morocco, although it is uncommon [1]. In addition to the sequenced specimens from Spain and the Czech Republic and the lectotype from Portugal, we know specimens from Italy, France, Germany, Hungary, and Poland.

***Remarks and comparative notes***. Unlike all other species, whose integument of the body is black, in this species the dorsum always shows a more or less distinct bronze or green reflection. The microsculpture of the pronotum and elytral interstriae is distinctly rugulose and thick, only similar to that of *R. cerdanicus*, in which, however, it is more opaque.

**(5)**  ***Rhamphus cerdanicus* group**

**a. *Rhamphus cerdanicus* Tempère, 1892** (Figure 3E)
*Rhamphus cerdanicus* Tempère, 1982: 15 [8].

***Type locality***. Bellver-de-Cerdaña (Lleida, Catalonia, Spain).

***Type series***. This species was described from four specimens: one male and one female collected in Spain (Lleida or Lerida), Bellver-de-Cerdaña, abords de la route qui vient de Puigeerda, à l’Est du village, 21 juin 1968, and two females from Pyrénées-Orientales, Cerdagne française, Targasonne, vers Angoustrine, 8 juillet 1962. We examined the holotype at MNHN in the Tempère collection.

***Non-type specimens***. Spain, Pais Vasco, Araba/Alava Elburgo, N42.812 W2.633, 550 m, 23.06.2007, lgt. Ugarte Salgueira (1 sequenced, RCCM); Spain, País Vasco, Araba/Álava, Elburgo-Burgelu, 556 m, N42.812 W2.633, 15.06.2023, *Prunus spinosa*, lgt. Iñigo Ugarte San Vicente (3 sequenced: 1 ITCB, 2 RCCM; 9, IUCA).

***Diagnostic redescription***. Length 1.2–1.4 mm. Integument of body black, nearly opaque. Legs with tarsi dark brown; antennae yellowish except for darker scape and club. Vertex of head convex. Antennae with clavate scape, twice longer than wide and slightly shorter than first funicular segment; this segment clavate and slightly larger, 1.5–1.6× longer than wide, distinctly more robust, twice as long as second segment; this segment 1.3–1.4× longer than wide; third and fourth segments nearly as long as wide; fifth-seventh segments transverse. Pronotum conical and moderately transverse (Pw/Pl 1.50–1.55), well-rounded in posterior half, with fairly marked transverse furrow behind its anterior edge, width of which is equal to barely more than half that of posterior edge. Its punctuation formed by large but shallow points on disc, which are tight, often contiguous, but not confluent. Elytra short (El/Ew 1.15–1.20), slightly longer than pronotum (Ew/Pw 1.14–1.18), with strongly punctuated striae, punctures close to each other, their diameter being, on average, greater than length of intervals that separate them. Interstriae slightly convex; almost all clearly wider than striae, with rugulose microsculpture. Metafemora distinctly globose. Pro- and metatibiae with small uncus, first tarsomere 3.0–3.2× longer than wide, second tarsomere 1.3–1.4× longer than wide; onychium 2.7–2.9× longer than wide. Body of penis elongated, with sides distinctly and gradually narrowed from base to apex, with subacute tip.

***Host plants***. In this last respect, Tempére [8] did not notice the host plant because he thought he had collected an already described common species. However, he recalled that only Rosaceae were present in the area where he collected, and that Salicaceae and Betulaceae were absent. At Elburgo-Burgelo (Spain), this species was collected on *Prunus spinosa* by Ugarte San Vicente.

***Distribution***. France (Eastern Pyrenees, French Cerdagne), Spain (Cerdanya, Basque Autonomous Community).

***Remarks and comparative notes***. The strong chamfered microreticulation on the dorsum and the shape of the penis easily distinguish this species from the others.

**(6)**  ***Rhamphus pulicarius* group**

**a.*Rhamphus pulicarius*(Herbst, 1795)** (Figure 3A and Figure 8B)
*Curculio pulicarius* Herbst, 1795:429 [41].
*Rhamphus pulicarius* (Herbst). Stephens, 1831: 197 [42]. Hustache, 1931: 398, 399 [37]. Hoffmann, 1958: 1357 [40]. Caldara et al., 2022: 375 [14]. Alonso-Zarazaga et al. 2023: 213 [1].

***Type locality***. Halberstadt (Sachsen-Anhalt, Germany)

***Type series***. Due to missing specimens of the type series, Caldara [14] recently designated the neotype of this species. It is a male already sequenced. Its online acc. number is KU909870 (NCBI database), with a photo taken when it was not yet glued on a card. It is deposited at the ZFMK.

***Non-type specimens***. About 200 specimens in addition to 28 sequenced specimens from England, France, Germany, Italy, Poland, and Serbia (Table S1).

***Diagnostic redescription***. Length 1.6–2.0 mm. Integument of body black, legs brownish, antennae yellow except for blackish club. Vertex of head moderately prominent. Rostrum with upper surface with 2–3 confused rows of punctures only at sides. Antennal scape twice as long as wide and slightly longer or as long as first segment of funicle; this segment 2.0–2.2× longer than wide, slightly thinner than scape, distinctly more robust and 1.3–1.4× longer than second segment; this segment 1.9–2.0× longer than wide; third and fourth segments 1.9–2.0× longer than wide; fifth-seventh segments transverse. Pronotum conical, transverse (Pw/Pl 1.75–1.85), widest in basal half, where sides are curvilinear, with robust and thick punctures. Elytra suboval, distinctly longer than wide (El/Ew 1.30–1.40), at base wider than pronotum (Ew/Pw 1.15–1.18), widest at apical third, weakly convex; striae clearly distinct, with thick punctures; interstriae slightly convex. Metafemora distinctly globose (l/w 2.5). Pro- and mesotibiae with robust uncus (l/w 4). First tarsomere 2.2–2.4× longer than wide, second tarsomere 1.2–1.4× longer than wide; onychium 3.8–4.1× longer than wide. Body of penis with rectilinear sides being gradually narrowed from base to apex.

***Host plants***. This species lives on *Populus* spp. (*P. alba*, *P. nigra*, *P. tremula*) and *Salix* spp. (*S. alba*, *S. aurita*, *S. babylonica*, *S. caprea*, *S. cinerea*, *S. daphnoides*, *S. hastata*, *S. pentandra*, *S. purpurea*, *S. repens*, *S. triandra*, *S. viminalis*). Our specimens that were molecularly studied were collected on *Salix alba*, *S. caprea*, and some undetermined *Salix*. The specimens previously reported on *Betula* spp. belong to *R. betulae* sp. nov. The citation of *R. pulicarius* on *Corylus avellana* L. [40] and on *Myrica gale* L. [43,44] must be confirmed.

***Distribution***. This species was previously cited in almost all the Palearctic region [1]. However, after our discovery of new cryptic species, its distribution could be reviewed, although probably not to a surprising level. In fact, the presence of this species, based on our molecular study, is currently confirmed for countries in Central, Western, and Eastern Europe.

***Remarks and comparative notes***. Due to the reddish first segment of the antennal funicle and the robust uncus of pro- and mesotibiae, this species can be confused with *R. crypticus* sp. nov., *R. betulae* sp. nov., and *R. pullus*. Only the first one of them lives on *Salix* spp., whereas the second and the third ones live on *Betula*; the last one, however, is currently only known from Japan.

**b. *Rhamphus crypticus* Toševski & Caldara sp. nov.** (Figure 3D)
LSID urn:lsid:zoobank.org:act:4C1DA04C-9AAA-4765-91A3-E9AB75D66D28

***Type locality***. Lynford (Mundford, West Norfolk, Great Britain).

***Type series***. Holotype, male: “England, 21/023, Lynford, Mundford, West Norfolk (VC28), TL8294, lgt. H. Mendel” (sequenced, BMNH). Paratypes: same data as holotype (1 sequenced, RCCM); “England, 21/026, Denge Beach, East Kent (VC15), TLR0817, 28.06.2021, lgt. H. Mendel” (7 sequenced: 6 HMCL, 1 ITCB); England, New Forest, S. Hants, SU2404, 13.07.2020, lgt. H. Mendel” (4 sequenced: 2 ITCB, 2, RCCM); “France, F 19, Latronche (19110), La Croix de Layre, 2.2525995969772343/45.29021453648623 16.VII.2020, battage, lgt. Lessieur David (1 sequenced, RCCM; 1, DLCQ); “France 65 - Poueyferré, 43.112498397/-0.093337389 29.06.2023, Tourbiére de Lourdes, lgt. D. Lessieur” (4 sequenced: 2 DLCQ, 2 ITCB; 1, DLCQ); “France - 65 Esparros, 43.0262449/0.280096, 2.07.2023, lgt. D. Lessieur” (1 sequenced, ITCB); “France - 65 Banios, 43.0409039/0.233232, 1.07.2023, lgt. D. Lessieur” (2 sequenced: 1 DLCQ, 1 ITCB); “France - 65 Pouzac, 43.076836910/0.119550038, 28.06.2023, gardeloup, lgt. D. Lessieur” (3 sequenced: 1 DLCQ, 2 ITCB); “Spain, provincia de Soria, Vinuesa, río Revinuesa, 13-VII-2020, lgt. Iñigo Ugarte San Vicente” (2 sequenced, ITCB; 10, IUCA); “Segovia, Real Sitio de San Ildefonso, embalse del Pontón Alto, N40°54′49” W04°01′47”, 1101 m, 2-VII-2023, I. Ugarte y F. Salgueira leg./on *Salix salviifolia* (5, IUCA).

***Non-type specimens***. None.

***Diagnostic description***. As *R. pulicarius*, except antennae with at least scape slightly darker than first funicular segment, sometimes also other segments dark brown, scape 1.8× longer than wide, slightly shorter than first segment of funicle, which is 1.5× longer than wide. Body of penis long, with parallel sides.

***Etymology***. The name of this species highlights its significant morphological similarity to *R. pulicarius*.

***Host plants***. The specimens of the type series were collected by beating several plants of the genus *Salix*, partly identified as *Salix atrocinerea* Brot. by D. Lessieur and *S. salviifolia* Brot. by D. Lessieur and I. Ugarte San Vicente.

***Distribution***. Great Britain, France, and Spain.

***Remarks and comparative notes***. The unusual character of several specimens of this species is represented by the dark color of funicular segments never encountered in other species. It undoubtedly shares a close relationship with *R. pulicarius*, as they both inhabit *Salix* spp.

**c. *Rhamphus pullus* Hustache, 1920 (stat. rev.)** (Figure 3B)
*Rhamphus pullus* Hustache, 1920: 635 [45]. Kôno, 1930: 30 [46]. Kôno, 1935: 62 [47]. Voss, 1958: 108 [48]. Morimoto, 1962: 187 [49]; 1984: 20 [32]. Nakane, 1963: 378 [50].

***Type locality***. Lake Chȗzenji (Nikkô, Tochigi prefecture, Kantō, Japan).

***Type series***. This species was described from nine specimens collected at Chȗzenji and Tokio. None of these specimens were found in Hustache’s collection at MNHN (H. Perrin, pers. comm.). According to Hustache [45], the Japanese species is distinguished from *R. pulicarius* of Europe, with which it shares “les antennes testacees (massue except noir)”, by “sa taille moindre, sa tete un peu plus allongee, les elytres moins fortement elargis en arriére”. Morimoto [32] reported that he cannot find any difference between Japanese and European specimens and considered these two species as synonyms.

***Non-type specimens***. “Japan, Mikuni pass., Yamanakako vlg., Yamanashi pref., 12.06.2021, lgt. Y. Notsu (3 sequenced, RCCM); Shikoku, Namerikawa, Ehime Pref., 29.VI.1975, Y. Notsu leg. (1 sequenced, ITCB). These specimens fit well with Hustache’s original description.

***Diagnostic redescription***. As *R. pulicarius*, except for scape 1.5× longer than wide, slightly longer than or as long as first segment of funicle, which is 1.5× longer than wide, as robust as scape, and distinctly more robust and 1.3–1.4× longer than second segment, which is 1.4–1.5× longer than wide; third and fourth segments 1.2–1.3× longer than wide; fifth-seventh segments transverse. Body of penis longer and with parallel sides.

***Host plants***. The specimens that we studied were collected by beating leaves of *Betula* sp. (Notsu, pers. com.). Morimoto [32] reported that in Japan the adults of this species feed on the leaves of numerous trees belonging to various families (*Malus pumila*, *Prunus tomentosa*, *P. avium*, *Betula platyphylla* var. j*aponica*, *Castanea crenata*, and *Quercus acutissima*), whereas the larvae have been collected from the leaves of *Malus pumila*. However, we very recently had the opportunity to study specimens collected in Japan (Oita Pref., Bungo-Ono C., Mt. Hontani, 1642 m alt., 1.VI 2024, Takashi Satoh leg.) on *Sorbus commixta* Hedl. and found that they are different from *R. pullus* and the other species herein considered both morphologically and molecularly. They need further study, but it seems likely also on the basis of similarities with the other species studied here that at least the specimens collected on Rosaceae in Japan do not belong to *R. pullus*; therefore, Morimoto’s data must be completely reconsidered.

***Distribution***. The current distribution of this species in Japan (Hokkaido, Honshū, Shikoku, Kyūshū, Tsushima) [32] and China (Fujian) [48] must be absolutely revised (see Biology section). The presence of this species was confirmed for the following Japanese regions of the Honshū island: Kantō and Chūbu.

***Remarks and comparative notes***. We consider *R. pullus* to be a distinct species, different from *R. pulicarius*, based on molecular and a few morphological differences, which contrasts Morimoto’s conclusion (see the above reported diagnosis). The former species seems very closely related to *R. betulae*, which is the only other species of *Rhamphus* living on *Betula*. Presently, these species are widely separated in their distribution. However, we currently do not know to which species the specimens collected in Asia and identified as *R. pulicarius* actually belong. It differs from *R. betulae* by longer segments of antennal funicle and by longer and parallel-sided body of penis.

**d. *Rhamphus betulae* Toševski & Caldara sp. nov.** (Figure 3C)
LSID urn:lsid:zoobank.org:act: 945DE07C-78E2-45DE-932E-8E3AC2B43A05

***Type locality***. Monte Tovo (Valsesia, Vercelli, Piedmont, Italy).

***Type series***. Holotype, male: “Val Sesia (VC) Monte Tovo 1200 m. 12. 06. 2021, L. Diotti” (sequenced, MSNM). Paratypes: same data as holotype (7 sequenced: 4 ITCB, 3 RCCM; 6, CBCC); “Val Sesia (VC) Monte Tovo 1200 m. 7.05.2011 L. Diotti” (3, CBCC); “Val Sesia (VC) Mt. Tovo mt. 1100, 7.05.2011 Monzini S.” (1, SMCM); “Val Sesia (VC) Monte Tovo 1200 m. m. 25.05. 2016 L. Diotti” (3, CBCC); “Val Sesia (VC) Monte Tovo 1200 m. 14.05.2022 L. DIOTTI (11, CBCC); “Val Sesia (VC) Mt. Tovo mt. 1100, 14.05.2022 Monzini S.” (5, SMCM); “Val Sesia (VC) Monte Tovo 1200 m. 8.06.2022 L Diotti” (19, CBCC); “Val Sesia (VC) Mt. Tovo mt. 1100, 8.06.2022, Monzini S.” (8, SMCM); “Val Sesia (VC) Monte Tovo 1200 m. 8.06.2022, M. Tedeschi” (12, MTCM); “France, 65 Trébons, 43.0799872049/0.113917399, 30.06.2023 bouleau, lgt. D. Lessieur” (7 sequenced: 6 DLCQ, 1 ITCB); “France - 65 Pouzac, 43.0758181239/0.116744447, 2.07.2023, lgt. D. Lessieur” (2 sequenced, ITCB); “Poland, Rudnik ad Lublin 51°16′58.8” N 22°38′28.8” E, 15.06.2021, lgt. Rafal Gosik” (9 sequenced: 5 ITCB, 2 RCCM, 2 RGCL).

***Non-type specimens***. None.

***Diagnostic description***. As *R. pulicarius*, except for scape, which is 1.5× longer than wide, as long as first segment of funicle, which is 1.3× longer than wide, as robust as scape, and distinctly more robust and 1.3–1.4× longer than second segment, which is 1.1–1.2× longer than wide; third and fourth segments are as long as they are wide; fifth-seventh segments transverse. Penis short and with convergent sides.

***Etymology***. The name of this species, a Latin feminine singular genitive, refers to the plant genus from which it was collected.

***Host plants***. This species was collected by beating leaves of *Betula* spp., like *R. pullus*.

***Distribution***. Italy, France, and Poland. This species probably has a larger distribution, at least in Europe.

***Remarks and comparative notes***. Concerning the differences from *R. pulicarius*, see the diagnosis. It differs from *R. pullus* by shorter segments of antennal funicle and shorter body of penis, with sides convergent from base to apex.

**(7)**  ***Rhamphus loebli* group**

**a. *Rhamphus loebli* Germann & Colonnelli, 2018** (Figure 3F)
*Rhamphus loebli* Germann & Colonnelli, 2018: 192 [10].

***Type locality***. Guarda (Beira Alta, Portugal).

***Type series***. This species was described on many specimens collected in various lowland and montane localities of central (Beira Baxa and Beira Alta, Serra da Estrela) and southern (Algarve) Portugal. We examined two paratypes from Serra da Estrela (Penhas da Saúde, m 1300).

***Non-type specimens***. “Spain, E. Castilia, Srr. Francia, La Alberca, env., 1075 m, 40°31′49” N, 06°08′44” W, 25.05.2019, lgt. Kratky” (1 sequenced, ITCB; 1, JKCH); “Spain, E. Andalucia, 4 km N of Rociana del Condado, N37 20.561 E6 36.165, 10.03.2011, lgt. J. Kratky (2 sequenced, RCCM; 1, JKCH).

***Diagnostic redescription***. Length 1.3–1.9 mm. Body shining black, with scape and first two segments of antennal funicle reddish-brown and others and club darker. Vertex of head prominent. Scape twice as long as wide, as long as first funicular segment, also being twice as long as wide; second and third twice as long as wide; fourth and fifth globular; sixth and seventh distinctly transverse. Pronotum twice as wide as long, widest behind middle, surface microreticulate and irregularly punctate, punctures with very short, indistinct white hairs. Elytra oval (El/Ew 1.20–1.30), widest just behind middle (Ew/Pw 1.30–1.33), with rounded sides. Femora robust, metafemora very thick, three times as wide as pro- or metafemora; pro- and mesotibiae with long uncus at outer angle reaching far beyond apex of tibiae. First tarsomere 2.8–3.0× longer than wide, second tarsomere as long as wide; onychium 3.8–4.1× longer than wide. Body of penis elongated, converging towards apex, with rounded and narrow tip; in lateral view regularly bowed and attenuated towards apex, with complex, elongated spine-like sclerites in endophallus.

***Host plants***. In Portugal, *R. loebli* was collected at two localities, both at montane altitudes above 1000 m, from a yellow flowering *Halimium* species belonging to the family Cistaceae. In the Algarve, the new species was again collected from an unknown *Halimium* species close to sea level.

***Distribution***. Portugal, Spain (new report).

***Remarks and comparative notes***. A species easily distinguishable from the western Palearctic ones by its distinctly prolonged pro- and mesotibial unci, very globose metafemora (more globose than in all other species), and shape of penis.

**(8)**  ****Rhamphus hisamatsui** group**

**a. *Rhamphus hisamatsui* Chûjô & Morimoto, 1960** (Figure 3H)
*Rhamphus hisamatsui* Chûjô & Morimoto, 1960: 4 [51].

***Type locality***. Hachijo Islands (Japan).

***Type series***. This species was described based on a dozen specimens collected in the type locality in various dates during 1957–1958.

***Non-type specimens***. “Japan, Inugoeji forest, Yamakita town, Kanagawa pref., 28.06.2021, lgt. Y. Notsu” (6 sequenced, ITCB); “Japan, Mikuni pass., Yamanakako vlg., Yamanashi pref., 12.06.2021, lgt. Y. Notsu” (1 sequenced, ITCB); Central Japan, Oritate Spa, Yunotani Vill., Niigata Pref., Y. Notsu leg.” (1, RCCM)

***Diagnostic redescription***. Length 1.50–1.75 mm. Body black, clothed with suberecumbent to suberect grayish pubescence on dorsum. Antennae yellowish in color and adorned with piceous club. Vertex of head distinctly prominent. Rostrum almost impunctate and smooth with few punctures at basal area and with distinct punctured stria on each side. Pronotum transverse (Pw/Pl 1.55–1.68), narrower at apical extremity than at basal end, distinctly emarginated at front border, strongly rounded at each side; dorsum gently convex, coarsely and rather reticulately punctured, with punctures with subrecumbent long hair-like scales, with small, elongate, and irregular-shaped impunctate area just behind middle. Elytra elliptical, moderately elongate (El/Ew 1.30–1.35), slightly wider at humeral area than widest part of pronotum (Ew/Pw 1.30–1.35); dorsum convex, striae regularly punctate (each puncture bearing fine pubescence); interstriae slightly costate, roughly structured, with file of pubescent-punctures on each interstria. Metafemora distinctly globose; pro- and mesotibiae with robust uncus; first tarsomere 2.7–2.9× longer than wide; second tarsomere 1.2–1.3× longer than wide; onychium 3.4–3.7× longer than wide; claws strongly appendiculate. Body of penis elongate, wider at the basal half, then narrowed with subparallel sides, with acute tip, endophallus with complex of elongate spine-like sclerites.

***Host plants***. The adults were captured on the leaves of *Alnus firma*, *A. japonica*, *Betula ermanii*, *B. phtyphylla* var. *japonica*, and *Acer mono*. The new adults were obtained by rearing the round blotchy mines of the leaves of *Acer pictum* ssp. *mono*. This species is abundant in the Izu Islands on *Alnus firma* but not found in the lowlands of Honshu, Shikoku, and Kyushu.

***Distribution***. Japan (Hokkaido, Honshu, Shikoku, Kyushu, and Izu Islands), Taiwan, the Russian Far East (Kuril Islands), and South Korea [1].

***Remarks and comparative notes***. This species is easily distinguished from the known Palearctic species of the genus by the vestiture of the dorsal surface composed of suberect hair-like scales and different shape of penis. It might be related to some Australian and Southern African species [7].

### 3.4. Dichotomous Key to the Groups with a Synoptic Key to the Species

1. Dorsal vestiture with distinct hair-like scales, which are recumbent on pronotum and subrecumbent on elytra. Claws appendiculate. On Sapindaceae and Betulaceae ........................................................................................................................................................... ***R. hisamatsui* group** (monobasic)
  (a) ***R. hisamatsui* Chûjô & Morimoto** (Figure 3H). Length 1.4–1.6 mm. Far Eastern Palearctic countries.

− Dorsal vestiture without or at least with recumbent, indistinct, very short hair-like scales. Claws without appendices ...........................................................................…....………………………………………………………….…………………………... **2**

2. Pro- and mesotibiae with long and thin uncus at outer angle reaching far beyond apex of tibiae. Metafemora very big. Corbels of metatibiae at the margin with conspicuous row of short and strong black thorns around apical half, reaching up half corbel length at outer margin, and only with sparse bright long spines. On *Halimium* (Cistaceae) ...………......……………………………….……….……………………..…………….……..……………. ***R. loebli* group** (monobasic)
  (a) ***R. loebli* Germann & Colonnelli** (Figure 3F). Length 1.4–1.8 mm. Portugal, Spain.

− Pro- and mesotibiae with shorter uncus, hardly reaching beyond apex of tibiae. Metafemora smaller. Corbels of metatibiae mainly with long, stiff, and dense erect bright spines; black thorns are quite inconspicuous and only around the apex………................................................................................................................................................................................................. **3**
3. Elytra and pronotum with distinctly rugulose, thick, somewhat opaque microsculpture……….............................................................................................................................................................................. **4**
− At least the elytral surface is rather shiny.…...................................................................................................................................................................................................... **5**
4. Elytra black, without any metallic reflection. Body of penis slender and longer, distinctly gradually narrowing from base to apex in dorsal view, with apex delimited with thin membranous line. On *Prunus spinosa* (Rosaceae) ..............................................………………………………………………………………………...… ***R. cerdanicus* group** (monobasic)
  (a) ***R. cerdanicus* Tempére** (Figure 3E). Length 1.3–1.4 mm. France (Eastern Pyrenees), Northern Spain.

− Elytra black with slight but evident bronze or greenish metallic shine. Body of penis shorter, with subparallel sides in dorsal view. On *Crataegus* (Rosaceae) ........................................................................................................ *** R. subaeneus* group** (monobasic)
  (a) ***R. subaeneus* Illiger** (Figure 3G). Length 1.5–1.8 mm. All of Europe.

5. Antennal scape subcylindrical, nearly as long as first funicular segment, and reddish like first funicular segments. Spaces of pronotal disc among punctures larger and more regular. Pro- and mesotibiae with distinctly more robust uncus (Figure 8B). Penis in lateral view curved only along its basal portion, then almost straight towards apex. On Salicaceae and Betulaceae ........................................................................................................…….....…….....…….....……..... ***R. pulicarius* group** (four species)
  (a) ***R. pulicarius* (Herbst)** (Figure 3A). Scape twice as long as it is wide, slightly longer, or as long as first segment of funicle, which is twice as long as it is wide. Body of penis long, with sides convergent from base to apex, with length of 1.5–2.0 mm. It is likely to be found throughout Europe on *Salix* spp.
  (b) ***R. crypticus* Toševski & Caldara sp. nov.** (Figure 3D). Scape 1.5× longer than wide and slightly shorter than segment of funicle, which is 1.5× longer than wide; antennae sometimes almost completely dark. Body of penis long, with parallel sides, and 1.5–2.0 mm in length. Great Britain, France, and Spain. *Salix* spp.
  (c) ***R. pullus* Hustache** (Figure 3B). Scape 1.5× longer than wide, slightly longer or as long as first segment of funicle, which is 1.5× longer than wide. Body of penis long, with parallel sides. Length 1.5–2.0 mm. Japan. *Betula* spp.
  (d) ***R. betulae*** **Toševski & Caldara sp. nov.** (Figure 3C). Scape 1.5× longer than wide, as long as first segment of funicle, which is 1.3× longer than wide. Body of penis short with sides convergent from base to apex. Length 1.5–2.0 mm. Italy, France, and Poland. *Betula* spp.

− Antennal scape clubbed, much shorter than first funicular segment, and distinctly darker than first funicular segments. Spaces of pronotal disc among punctures narrower, confused, and irregular. Pro- and mesotibiae with distinctly smaller uncus (Figure 8A,C). Body of penis in lateral view strongly curved at least along its entire basal half..................................................................……....................................................................................................................………… **6**
6. Pro- and mesotibiae with thinner uncus (Figure 8C). First segment of antennal funicle 1.7–1.8× longer than wide, subglobose, and widest at middle. Body of penis in dorsal view very stout, with sclerotized portion largely expanded over its dorsal portion; in lateral view, it uniformly tapers towards apex only at distal third. On *Prunus spinosa* and *P. cerasifera* (Rosaceae).................***R. monzinii* group** (three species)
  (a) ***R. monzinii*** **Pesarini & Diotti** (Figure 2D). Second segment of antennal funicle 1.8–2.0× longer than wide, with elliptical elytral shape (El/Ew 1.25–1.30). Body of penis with sides slightly divergent in apical half, in lateral view with shorter apex. Length 1.2–1.5 mm. Northern Italy, Serbia, and Greece.
  (b) ***R. diottii* Toševski & Caldara sp. nov.** (Figure 2E). Second segment of funicle 2.0–2.2× longer than wide, with elliptical elytral shape (El/Ew 1.20–1.25). Body of penis with sides slightly convergent from base, in lateral view with longer apex. Length 1.4–1.7 mm. Serbia.
  (c) ***R. ibericus* Toševski & Caldara sp. nov.** (Figure 2F) Second segment of funicle 1.9–2.1× longer than wide. Elytra narrow (El/Ew 1.30–1.35), with sides only little rounded. Body of penis with parallel sides, not enlarged in apical third. Length 1.4–1.6 mm. Northern Spain.

− Pro- and mesotibiae with more robust uncus (Figure 8A). First segment of antennal funicle 2.0–2.2× longer than wide, subconical, and gradually enlarged from base to apex. Penis in dorsal view moderately stout, its sclerotized portion not or only very slightly extended dorsally, and in lateral view gradually tapers towards apex at more than its distal half. On Rosaceae…...................................................…………………………....................................................................................................... **7**
7. Body of penis in dorsal view with sides slightly narrowing from base to apex (Figure 2A–C). Associated with diverse genera of Rosaceae (*Crataegus*, *Pyrus, Mespilus*, and *Prunus*)........……………...………………………………………………………...…...………… ***R. oxyacanthae* group** (three species)
  (a) ***R. oxyacanthae* (Marsham)** (Figure 2A). Pronotum moderately conical (Pw/Pl 1.60–1.70). First tarsomere 2.2–2.5× longer than wide, second tarsomere 1.2–1.4× longer than wide. Body of penis in dorsal view nearly similar in width from base to apex, with rectilinear sides slightly narrowing from base to apex. Length 1.3–1.7 mm. Probably all of Europe.
  (b) ***R. bavierai* Diotti, Caldara & Toševski** (Figure 2C). Pronotum moderately conical (Pw/Pl 1.60–1.70). First tarsomere 3.0–3.3× longer than wide, second tarsomere 1.6–1.8× longer than wide. Body of penis in dorsal view with rectilinear sides slightly narrowing from base to apex. Length 1.4–1.9 mm. Sicily.
  (c) ***R. hampsicora* Diotti, Caldara & Toševski** (Figure 2B). Pronotum distinctly conical (Pw/Pl 1.75–1.85). First tarsomere 2.2–2.5× longer than wide, second tarsomere 1.2–1.4× longer than wide. Body of penis in dorsal view with slightly curved sides, moderately narrowing from base to apex. Length 1.5–2.0 mm. Sardinia.

− Body of penis in dorsal view with parallel sides (Figure 2G,H). On *Crataegus*................................................................................................................................................ ***R. cypricus* group** (two species)
  (a) ***R. cypricus* Toševski & Caldara sp. nov.** (Figure 2G) Pronotum moderately conical (Pw/Pl 1.50–1.55). First tarsomere 2.7–2.9× longer than wide, second tarsomere 1.1–1.3× longer than wide. Length 1.4–1.7 mm. Cyprus.
  (b) ***R. macedonicus* Toševski & Caldara sp. nov.** (Figure 2H) Pronotum distinctly conical (Pw/Pl 1.45–1.50). First tarsomere is 2.8–3.1× longer than wide, second tarsomere 1.1–1.3× longer than wide. Length 1.2–1.5 mm. Greece.

## 4. Discussion

The rapid development of molecular techniques after the 1990s, along with the DNA barcoding initiative launched by Hebert [52], provided better taxonomic resolution in species recognition efforts [16]. Recently, species recognition has become an important issue if we consider the rapid decline in traditional taxonomic knowledge [53]. Nevertheless, species concept and species limits continue to be central challenges in both basic and applied biological studies, especially when comparing taxonomic entities [54]. There is still much debate about the use of solely mtDNA barcodes for delimiting species boundaries, especially in species with a high range of intraspecific genetic diversity. In many cases, deep mitochondrial genetic divergence can conceal the existence of a cryptic species [12], making barcoding identification only a preliminary step to determine species limits, especially with respect to closely related species. Nevertheless, mtDNA has many favorable properties and is widely used, especially for low-level taxonomy; nevertheless, it should be used with caution and accompanied by nuclear DNA (nDNA) marker analysis [55]. An increasing number of studies that elaborate phylogenetic relationships demonstrate the importance of precisely defined species limits between closely related taxa grouped in lower taxonomic categories such as tribes and genera. However, when there are no visible morphological differences between species and significant genetic differences, it may indicate not only the existence of cryptic species but also complex evolutionary patterns within the group being studied. When specific and significant properties, such as phytogeography, comparative morphology, population genetics, ecology, development, and behavior, are not mutually aligned, intricate discussions about the theoretical aspects of the species concepts can occur [56]. Systematics and taxonomy are precise, and exact biological disciplines in which unambiguous delineation and identification of species represent a central axis for understanding mutual interactions within a studied group of organisms.

Species from the genus *Rhamphus* face several challenges in precisely defining their taxonomical status. First, no morphological characteristics are present to delimit females among different species, excluding comparative studies as an effective method. Thus, delimitation is possible only by using genetic data. With respect to male delimitation in the genus *Rhamphus*, the species are clustered into morphologically well-defined groups mainly on the basis of characters present on the shape of the penis. However, genetic analyses are necessary to determine the taxonomical status of the cryptic species within the groups.

In the western Palearctic, we identified a total of 14 entities at the species level, which were clustered into eight morphotypic groups that mainly followed the characteristics present on male genitalia. These groups do not follow a unique evolutionary pattern and exhibit different genetic peculiarities. Extensive species radiation with characteristics of cryptic speciation was recorded within three groups, in which the full species status was assigned to six entities, including *R. diottii* sp. nov., *R. ibericus* sp. nov. (*monzinii*-group), *R. cypricus* sp. nov., *R. macedonicus* sp. nov. (*cypricus*-group), *R. betulae* sp. nov., and *R. crypticus* sp. nov. (*pulicarius*-group). The species limits of the newly described species were supported by the significant divergence recorded for all three genetic markers—mt*COI*, and the two nuclear markers n*EF-1α* and n*CAD*—used in our study. Genetic divergence between species was the most pronounced for the mt*COI* gene, which confirms the value of this marker for species recognition. However, better phylogenetic resolution was obtained by analyzing more conservative nuclear markers. Both selected nuclear markers are protein-coding single-copy genes and are thus suitable and applicable for species limit analysis [57,58]. For this reason, phylogenetic relationships were assessed using comparative analysis of tree topology obtained from three independent analyses. Although discordance between mtDNA and nDNA phylogeny is expected because of differences in ploidy, mode of inheritance, and the effective population size [59], conflicts in the tree topologies were negligible among the applied genetic markers. However, in some cases, the results need to be discussed in more detail. Compared to that of nDNA, the divergence of mt*COI* is more pronounced because of the higher mutation rates of mitochondrial DNA. Furthermore, mitochondrial DNA does not undergo recombination, at least not in insects, and has a fourfold lower effective population size compared with nuclear genes [59,60]. A threshold value of 3% genetic distance was considered for the commonly used mt*COI* barcode gene to delineate closely related species [52]. These properties make mtDNA genes the markers of choice for resolving lower-level taxonomic issues [55]. Considering this, analysis based on the mt*COI* gene marker revealed an almost entirely well-resolved phylogeny of the species within the genus *Rhamphus*, which is consistent with the obtained morphological data and the newly described species in our study.

Phylogeny based on the nuclear coding gene marker n*EF-1α* has produced a phylogenetic tree in which the positions of species groups and species within are well resolved for the majority of *Rhamphus* species (Figure 6A). Unresolved issues within the *R. pulicarius* group are related to the position of three species within this group. In the western Palearctic, the *pulicarius* group includes *R. pulicarius* (associated with various *Salix* species*), R. betulae* sp. nov. (associated with *Betula* species), and *R. crypticus* sp. nov. (associated with *Salix alba*, *Salix atrocinerea*, and *S. salviifolia*). The species from Japan, *R. pullus*, was synonymized by Morimoto [32] with the European *R. pulicarius*. However, we considered *R. pullus* as a separate species on the basis of the molecular data and a few morphological differences. The results indicate that *R. pullus* shares a close relationship not with *R. pulicarius* but with *R. betulae* sp. nov. In addition, both *R. pullus* and *R. betulae* sp. nov. are associated with *Betula* spp. as host plants. The close genetic and ecological connections between these species suggest that they evolved separately in allopatry. Therefore, it appears that the formation of new species occurred because the *Rhamphus* populations linked to *Betula* spp. experienced dispersal and range expansion eastward from the western Palearctic to the Far East during interglacial or stadial periods. As a consequence, range expansion led to genetic divergence of previously isolated allopatric populations. Such range shifts during the Quaternary period are well-documented [61]. However, it will be interesting to study additional specimens across Siberia and China to confirm this statement and better understand the speciation processes of the *pulicarius* group.

Genetic data based on the mt*COI* and n*CAD* genes are consistent with the observed divergence of the above three species. However, the n*EF-1α* phylogeny shows signs of discordance with aforementioned genes because of shared haplotypes between *R. pulicarius* and *R. betulae* sp. n (bet6_Ef and bet12_Ef; Figure 6A). It is difficult to pinpoint whether demographic, evolutionary, and/or biogeographic reasons lie beneath the observed discordance [62]; however, regardless of the reason discordance exists in this group, it appears that n*EF-1α* is lagging behind mt*COI* and n*CAD* in delimitating species, at least within the *pulicarius* group [63].

Phylogeny based on n*CAD* separated all the species groups within the genus *Rhamphus* but expressed a slightly different topology than that of n*EF-1α*. This can be attributed to variable evolutionary rates of different nuclear protein-coding genes [64]. With the exception of *R. cypricus* sp. nov. (Cyprus) and *R. macedonicus* sp. nov. (Greek Macedonia - mainland) sharing an identical haplotype (cyp1_CAD and mac1-CAD; Figure 6B), the n*CAD* gene produced a nearly identical topology to that of mt*COI*. This finding is in accordance with the proposed utilization of the n*CAD* gene as a nuclear barcode and as a marker that can discriminate species in the same manner as mt*COI* can [58]. However, the fact that *R. cypricus* sp. nov. and *R. macedonicus* sp. nov. share identical haplotypes on n*CAD* but exhibit 4.2% differences in mt*COI* reflects the inconsistency not recorded in the mt*COI* or in the n*EF-1α* phylogeny. This inconsistency can be attributed to either introgression or incomplete lineage sorting at this point, and it seems that, inversely similar to the *pulicarius* group, n*CAD* lags behind mt*COI* and n*EF-1α* in delimitating species [63,65]. Considering this, it seems that nuclear gene polymorphisms have been maintained differently within the genus *Rhamphus*, i.e., showing differences between species groups and/or gene markers analyzed. Furthermore, it is notable that all the inconsistencies, meaning the nodes showing disagreement in the nuclear marker phylogenies, have lower node support (Figure 6). However, the phylogeny based on the partitioned multigene data did not show the aforementioned inconsistencies and adequately identified all the groups and the species within. Furthermore, it seems that the phylogeny based on the partitioned multigene dataset displayed the most adequate resolution of the relationships within the genus *Rhamphus* and with high nodal support (Figure 7). It is symptomatic that the node with weaker support is defining sister relationships of *oxyacanthae* and *pulicarius* groups, which are two groups with high intraspecies and interspecies diversity. It is possible that additional unsampled and/or undescribed species within these groups would contribute to higher nodal support.

Three cryptic species from the *monzinii* group, *R. monzinii*, *R. diottii* sp. nov., and *R.ibericus* sp. nov., are well-distinguished from other *Rhamphus* species by the typical shape of the penis. *Rhamphus monzinii* and *R. diottii* sp. nov. are distributed in the Apennine and Balkan Peninsula, while the third species, *R. ibericus* sp. nov., is distributed in northwestern Spain. The recorded host plant of *R. ibericus* sp. nov. is *Prunus spinosa*. The same host plant was recorded for *R. diottii* sp. nov., while the host plant for *R. monzinii* is *Prunus cerasifera* and rarely *P. spinosa*. *Rhamphus monzinii* is probably the most common species on the Balkan Peninsula, forming dense populations on *Prunus cerasifera*. In contrast, *R. diottii*, which is associated with *P. spinosa*, has a sparse distribution and has, to date, only been found only in lowland habitats along large rivers in Northeast Serbia (South Banat and North Srem). Nevertheless, there is a published sequence [66] in the Boldsystems database of the specimens determined as *Rhamphus oxyacanthae* under accession number KU915925 (NCBI Database) with 99.51% identity with sequences of *R. diottii* sp. nov. from Serbia. The origin of this specimen is Blankenburg, Saxony-Anhalt, Germany, which undoubtedly confirms the wider geographical distribution of *R. diottii* sp. nov. in Europe. According to pairwise distances, we assumed that the species radiation of the *monzinii* group started at the beginning of the Pliocene, following a dramatic geographic event related to the Messinian salinity crisis approximately 6 million years ago, which enabled land communication across the whole Mediterranean region. For mt*COI, R. ibericus* sp. nov. diverges from the east-distributed species *R. monzinii* and *R. diottii* sp. nov. in an average of 12.7% and 13.5%, respectively, which strongly suggests the vicariance event of *R. ibericus* sp. nov. On the other hand, the average genetic difference between *R. monzinii* and *R. diottii* sp. nov. is about 9.6%, indicating that this speciation occurred during the late Pliocene and Quaternary periods [67]. The results of the analysis of the mt*COI*, n*EF-1α*, and n*CAD* genes, along with the results of the median-joining haplotype network analysis, strongly support that species within the *monzinii* group are clearly defined and have distinct lineages. The observed trend in the differentiation of cryptic species within this group indicates that more cryptic species are expected to exist along Mediterranean isles, the Middle East, and Central Asia.

Three species, *R. subaeneus*, *R. cerdanicus*, and *R. loebli*, assigned here as separate groups, are of particular interest for understanding mutual connectivity among species from the genus *Rhamphus* in the western Palearctic and beyond. In contrast to the other groups, these species are easily distinguished from each other as well as from all the other species. *Rhamphus subaeneus* is the only species showing more or less distinct bronze or green sheen not found in other species, while *R. cerdanicus* and *R. loebli* are discriminated by different penis shapes. Nevertheless, the average pairwise distances of *R. loebli* with respect to those of other *Rhamphus* species were high, ranging 15.4–17.6%, 9.9–12.1%, and 13.2–15%, for mt*COI*, n*EF-1α*, and n*CAD*, respectively. In addition to having curious genetic properties, *R. loebli* is the only species from the western Palearctic associated with *Halimium* sp. from the rockrose family Cistaceae. Cistaceae plants are widely distributed in the Mediterranean region; thus, more undiscovered *Rhamphus* species are expected.

Research on contemporary, archival/museum specimens, especially type specimens, is important for determining the precise taxonomic status of a studied organism. Furthermore, understanding of their morphological, ecological, and behavioral aspects contributes to our hypotheses about species limits and the treatment of these species. When analysis of selected genetic markers confirms these hypotheses, it leads to understanding the taxonomic position of all entities and, consequently, to better understanding the evolutionary processes. Considering our findings and de Queiroz’s [56] concept that species represent separately evolving lineages and that evidence of lineage separation is evidence of the existence of different species, our hypotheses about the treatment of *Rhamphus* species in this study are justified and confirmed.

## Data Availability

All molecular sequence data is available on GenBank and can be accessed with the accession numbers specified in the text and in Tables S1–S3.

## Supplementary Materials

The following supporting information can be downloaded at: https://www.mdpi.com/article/10.3390/insects16111123/s1, Table S1: List of Rhamphus specimens sequenced for mitochondrial COI gene; Table S2: List of Rhamphus specimens sequenced for nuclear EF-1α gene; Table S3: List of Rhamphus specimens sequenced for nuclear CAD gene; Table S4: Thermal protocol and reaction mix protocol for amplification of mtCOI, nEF-1α and nCAD gene; Table S5: Primers used for mtCOI amplification; Table S6: Primers used for nEF-1α amplification; Table S7: Primers used for nCAD amplification; Table S8 Pairwise analysis (p-distance method) among all recorded haplotypes of Rhamphus species on mtCOI, nEF-1α and nCAD genes. Figure S1: The primer scheme for nEF-1α amplification; Figure S2: The primer scheme for nCAD amplification.
