# Supplementary material for: An Integrative Revision of the Genus Rhamphus (Curculionidae) from the Western Palearctic: Morphological and Molecular Data Reveal the Radiation of Multiple Species
Source: Insects. 2025 Nov 3;16(11):1123. doi: 10.3390/insects16111123 (PMC12653807; doi:10.3390/insects16111123)
Supplement: Supplementary file 1 [file insects-16-01123-s001.zip › Figure_S1.pdf]

Figure S1. The primer scheme for n*EF-1α* amplification

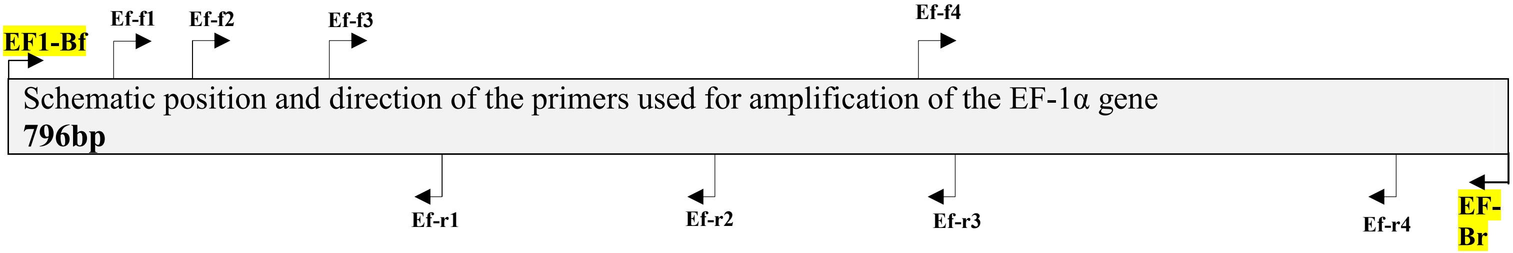

PCR product length amplified using corresponding *EF-1α* primers pairs (bp with primers included)					
**Primers**	**EF-Br**	**Ef-r1**	**Ef-r2**	**Ef-r3**	**Ef-r4**
**EF1-Bf**	**≈ 796 bp**	245 bp	396 bp	562 bp	672 bp
**Ef-f1**	≈ 752 bp	201 bp	352 bp	518 bp	628 bp
**Efr-f2**	≈ 714 bp	163 bp	314 bp	480 bp	590 bp
**Ef-f3**	≈ 630 bp	79 bp	230 bp	396 bp	506 bp
**Ef-f4**	≈ 303 bp	-	-	69 bp	179 bp
