# Supplementary material for: An Integrative Revision of the Genus Rhamphus (Curculionidae) from the Western Palearctic: Morphological and Molecular Data Reveal the Radiation of Multiple Species
Source: Insects. 2025 Nov 3;16(11):1123. doi: 10.3390/insects16111123 (PMC12653807; doi:10.3390/insects16111123)
Supplement: Supplementary file 1 [file insects-16-01123-s001.zip › Figure_S2.pdf]

Figure S2. The primer scheme for n*CAD* amplification

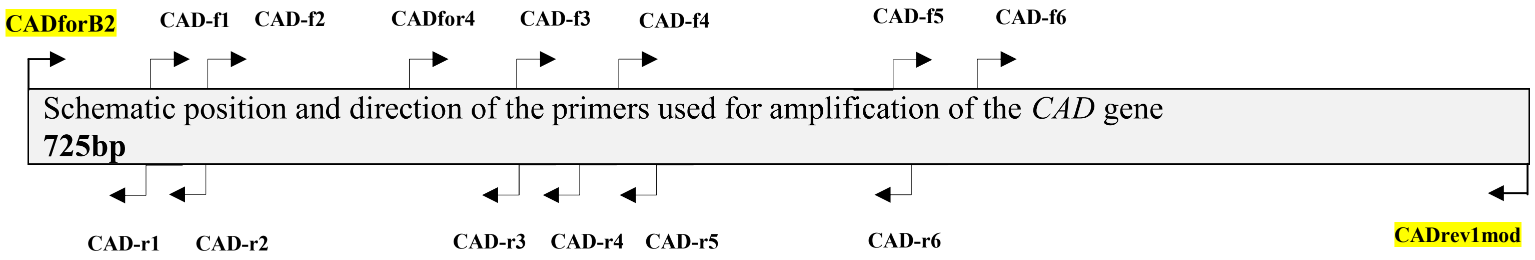

PCR product length amplified using corresponding CAD primers pairs (in bp with primers included)							
**primers**	**CADrev1mod**	**CAD-r1**	**CAD-r2**	**CAD-r3**	**CAD-r4**	**CAD-r5**	**CAD-r6**
**CADforB2**	**725 bp**	86 bp	113 bp	287 bp	329 bp	491 bp	524 bp
**CADfor4**	515 bp	-	-	77 bp	119 bp	281 bp	314 bp
**CAD-f1**	662 bp	-	50 bp	224 bp	266 bp	428 bp	461 bp
**CAD-f2**	635 bp	-	-	197 bp	239 bp	410 bp	434 bp
**CAD-f3**	461 bp	-	-	-	65 bp	227 bp	260 bp
**CAD-f4**	419 bp	-	-	-	-	185 bp	218 bp
**CAD-f5**	259 bp	-	-	-	-	-	58 bp
**CAD-f6**	226 bp	-	-	-	-	-	-
