# Supplementary material for: An Integrative Revision of the Genus Rhamphus (Curculionidae) from the Western Palearctic: Morphological and Molecular Data Reveal the Radiation of Multiple Species
Source: Insects. 2025 Nov 3;16(11):1123. doi: 10.3390/insects16111123 (PMC12653807; doi:10.3390/insects16111123)
Supplement: Supplementary file 1 [file insects-16-01123-s001.zip › Table_S1.pdf]

Table S1. List of *Rhamphus* specimens sequenced for mitochondrial *cytochrome c oxidase subunit I* gene (mt*COI*), sorted by DNA voucher code, species name, locality, country of origin, host plant affiliation, haplotype name with accession number in NCBI GenBank and frequency.

***Rhamphus oxyacanthae* (Marsham, 1802)**						
**Voucher DNA**	**Species**	**Location**	**Origin**	**Host**	**COI haplotype name (accession number)**	**Frequency**
5168	*R. oxyacanthae*	Italy, Basilicata, bosco di Accettura, 10.06.2018, lgt. L. Diotti	Italy	no data	oxy1_COI (MW879276)[a]	**2**
5170	*R. oxyacanthae*	Italy, Basilicata, bosco di Accettura, 10.06.2018, lgt. L. Diotti	Italy	no data		
5172	*R. oxyacanthae*	Italy, Muro Lucano (PT), Monte Paratiello, 10.06.2018, lgt. L. Diotti	Italy	no data	oxy2_COI (MW879277)[a]	**2**
5173	*R. oxyacanthae*	Italy, Muro Lucano (PT), Monte Paratiello, 10.06.2018, lgt. L. Diotti	Italy	no data		
5177	*R. oxyacanthae*	Italy, Emilia (Parma), Passo Cirrone 225 m, 8.08.2018, lgt. L. Diotti	Italy	no data	oxy3_COI (MW879278)[a]	**1**
5169	*R. oxyacanthae*	Italy, Basilicata, bosco di Accettura, 10.06.2018, lgt. L. Diotti	Italy	no data	oxy4_COI (MW879279)[a]	**3**
6015	*R. oxyacanthae*	F. 33, Queyrac, Le Gross Cap, 4.05.2018, lgt. Lessieur David	France	*Crataegus monogyna*		
6326	*R. oxyacanthae*	Italy, Sila Grande, (CS) Lago di Cecita, 17.06.2015, lgt. L. Diotti	Italy	no data		
5160	*R. oxyacanthae*	Italy, Giaglione, (TO), Val Clarea, 7.07.2018, lgt. L. Diotti	Italy	no data	oxy5_COI (MW879280)[a]	**1**
5182	*R. oxyacanthae*	Italy, Fino Mornasco, (CO), Ville dei Mulini, 25.05.2018, lgt. L. Diotti	Italy	no data	oxy6_COI (MW879281)[a]	**1**
5181	*R. oxyacanthae*	Italy, Fino Mornasco, (CO), Ville dei Mulini, 25.05.2018, lgt. L. Diotti	Italy	no data	oxy7_COI (MW879282)[a]	**1**
5159	*R. oxyacanthae*	Italy, Giaglione, (TO), Val Clarea, 7.07.2018, lgt. L. Diotti	Italy	no data	oxy8_COI (MW879283)[a]	**3**
5161	*R. oxyacanthae*	Italy, Giaglione, (TO), Val Clarea, 7.07.2018, lgt. L. Diotti	Italy	no data		
6012	*R. oxyacanthae*	F. 33, Queyrac, Le Gross Cap, 4.05.2018, lgt. Lessieur David	France	*Crataegus monogyna*		
5171	*R. oxyacanthae*	Italy, Basilicata, bosco di Accettura, 10.06.2018, lgt. L. Diotti	Italy	no data	oxy9_COI (MW879284)[a]	**1**
5178	*R. oxyacanthae*	Italy, Emilia (Parma), Passo	Italy	no data	oxy10_COI	**1**

		Cirrone 225 m, 8.08.2018, lgt. L. Diotti			(MW879285)[a]	
5953	*R. oxyacanthae* **NEOTYPE**	England, Suffolk, TL7086, 23.06.2020., lgt. H. Mandel	England	*Crataegus* sp.	oxy11_COI (MZ404333) [b]	**4**
5954	*R. oxyacanthae*	England, Suffolk, TL7086, 23.06.2020., lgt. H. Mandel	England	*Crataegus* sp.		
5955	*R. oxyacanthae*	England, Suffolk, TL7086, 23.06.2020., lgt. H. Mandel	England	*Crataegus* sp.		
5959	*R. oxyacanthae*	England, Suffolk, TL7086, 23.06.2020., lgt. H. Mandel	England	*Crataegus* sp.		
5956	*R. oxyacanthae*	England, Suffolk, TL7086, 23.06.2020., lgt. H. Mandel	England	*Crataegus* sp.	oxy12_COI (PV910589)	**1**
5957	*R. oxyacanthae*	England, Suffolk, TL7086, 23.06.2020., lgt. H. Mandel	England	*Crataegus* sp.	oxy13_COI (PV910590)	**1**
5958	*R. oxyacanthae*	England, Suffolk, TL7086, 23.06.2020., lgt. H. Mandel	England	*Crataegus* sp.	oxy14_COI (PV910591)	**1**
5332	*R. oxyacanthae*	Spain, San Vicente sa de logiz, Álava, 8.07.2018, lgt. Iñigo Ugarte San Vicente & Fernando Salgueira	Spain	*Crataegus monogyna*	oxy15_COI (PV910592)	**3**
5335	*R. oxyacanthae*	Spain, San Vicente de Arana, La Dehesa Álava, 8.07.2018, lgt. Iñigo Ugarte San Vicente & Fernando Salgueira	Spain	*Crataegus monogyna*		
6602	*R. oxyacanthae*	Spain, País Vasco, prov. de Araba/ Álava, Subijana de Álava, 518 m s.n.m., 25.06.2019, lgt. Iñigo Ugarte San Vicente & Fernando Salgueira	Spain	*Crataegus monogyna*		
4856	*R. oxyacanthae*	Serbia, Brusnik, Negotin, N44 6.489 E22 24.115, 322 m, 21.05.2018, lgt. Toševski	Serbia	*Cydonia oblonga*	oxy16_COI (PV910519)	**1**
4860	*R. oxyacanthae*	Serbia, Brusnik, Negotin, N44 6.489 E22 24.115, 322 m, 21.05.2018, lgt. Toševski	Serbia	*Pyrus* sp.	oxy17_COI (PV910520)	**2**
6350	*R. oxyacanthae*	Serbia, Vratarnica, Zaječar, 15.06.2021, N43 47.630 E22 18.125, 165 m., lgt. Toševski	Serbia	*Malus domestica*		
4854	*R. oxyacanthae*	Serbia, Pitot, Staničenje, 20.05.2018, N43 13.020 E22 30.556, 403 m, lgt. Toševski	Serbia	*Crataegus* sp.	oxy18_COI (PV910521)	**1**
4534	*R. oxyacanthae*	Serbia, Mt. Zlatibor, 9.07.2017, N43 47.310 E19 43.721, 662 m, lgt. Toševski	Serbia	*Crataegus* sp.	oxy19_COI (PV910522)	**2**
4892	*R. oxyacanthae*	Serbia, Vlasina, Božićki Kanal, N42 40.997 E22 21.888, 1289 m, 21.06.2018, lgt. Toševski	Serbia	*Prunus cerasifera*		
4895	*R. oxyacanthae*	Serbia, Vlasina, Božićki	Serbia	*Prunus*	oxy20_COI	**1**

		Kanal, N42 40.997 E22 21.888, 1289 m, 21.06.2018, lgt. Toševski		*cerasifera*	(PV910523)	
4537	*R. oxyacanthae*	Serbia, Mt. Zlatibor, 9.07.2017, N43 47.310 E19 43.721, 662 m, lgt. Toševski	Serbia	*Crataegus* sp.	oxy21_COI (PV910524)	**1**
4891	*R. oxyacanthae*	Serbia, Vlasina, N42 40.573 E22 18.818, 1246 m, 21.06.2018, lgt. Toševski	Serbia	*Crataegus* sp.	oxy22_COI (PV910525)	**1**
4907	*R. oxyacanthae*	Greece, Mt. Taygetos 10.07.2018, N37 04.155 E22 15.882, 1381 m, lgt. Toševski	Greece	*Pyrus spinosa*	oxy23_COI (PV910526)	**1**
5271	*R. oxyacanthae*	Greece, Mt. Taygetos 10.07.2018, N37 04.155 E22 15.882, 1381 m, lgt. Toševski	Greece	*Pyrus spinosa*	oxy24_COI (PV910527)	**1**
4908	*R. oxyacanthae*	Greece, Mt. Taygetos 10.07.2018, N37 04.155 E22 15.882, 1381 m, lgt. Toševski	Greece	*Pyrus spinosa*	oxy25_COI (PV910528)	**3**
5272	*R. oxyacanthae*	Greece, Mt. Taygetos 10.07.2018, N37 04.155 E22 15.882, 1381 m, lgt. Toševski	Greece	*Pyrus spinosa*		
5346	*R. oxyacanthae*	Greece, Mt. Taygetos 10.07.2018, N37 04.155 E22 15.882, 1381 m, lgt. Toševski	Greece	*Pyrus spinosa*		
5273	*R. oxyacanthae*	Greece, Mt. Taygetos 10.07.2018, N37 04.155 E22 15.882, 1381 m, lgt. Toševski	Greece	*Pyrus spinosa*	oxy26_COI (PV910529)	**7**
5274	*R. oxyacanthae*	Greece, Mt. Taygetos 10.07.2018, N37 04.155 E22 15.882, 1381 m, lgt. Toševski	Greece	*Pyrus spinosa*		
5275	*R. oxyacanthae*	Greece, Mt. Taygetos 10.07.2018, N37 04.155 E22 15.882, 1381 m, lgt. Toševski	Greece	*Pyrus spinosa*		
5276	*R. oxyacanthae*	Greece, Mt. Taygetos 10.07.2018, N37 04.155 E22 15.882, 1381 m, lgt. Toševski	Greece	*Pyrus spinosa*		
5343	*R. oxyacanthae*	Greece, Mt. Taygetos 10.07.2018, N37 04.155 E22 15.882, 1381 m, lgt. Toševski	Greece	*Pyrus spinosa*		
5344	*R. oxyacanthae*	Greece, Mt. Taygetos 10.07.2018, N37 04.155 E22 15.882, 1381 m, lgt. Toševski	Greece	*Pyrus spinosa*		
5345	*R. oxyacanthae*	Greece, Mt. Taygetos 10.07.2018, N37 04.155 E22 15.882, 1381 m, lgt. Toševski	Greece	*Pyrus spinosa*		

6013	*R. oxyacanthae*	F. 33, Queyrac, Le Gross Cap, 4.05.2018, lgt. Lessieur David	France	*Crataegus monogyna*	oxy27_COI (PV910593)	**1**
6014	*R. oxyacanthae*	F. 33, Queyrac, Le Gross Cap, 4.05.2018, lgt. Lessieur David	France	*Crataegus monogyna*	oxy28_COI (PV910594)	**1**
6016	*R. oxyacanthae*	F 19, 14.7.2020, lgt. Lessieur David	France	no data	oxy29_COI (PV910595)	**1**
6017	*R. oxyacanthae*	F 19, 14.7.2020, lgt. Lessieur David	France	no data	oxy30_COI (PV910596)	**1**
6095	*R. oxyacanthae* (NEOTYPE of *R. flavicornis*)	Swiss, 329_19.8 SZ, GR, Müstair, Wald, GS, Laubstreu, Corylus, N830'037 E168'456, 1350m, 15.06.2019, lgt. C. Germann	Swiss	*Crataegus* sp.	oxy31_COI (MZ404334)[b]	**1**
6327	*R. oxyacanthae*	Italy, Sila Grande, (CS) Lago di Cecita, 17.06.2015, lgt. L. Diotti	Italy	no data	oxy32_COI (PV910597)	**1**
6323	*R. oxyacanthae*	Italy, Sila Grande, (CS) Lago di Cecita, 17.06.2015, lgt. L. Diotti	Italy	no data	oxy33_COI (PV910598)	**3**
6324	*R. oxyacanthae*	Italy, Sila Grande, (CS) Lago di Cecita, 17.06.2015, lgt. L. Diotti	Italy	no data		
6325	*R. oxyacanthae*	Italy, Sila Grande, (CS) Lago di Cecita, 17.06.2015, lgt. L. Diotti	Italy	no data		
6411	*R. oxyacanthae* (larva L3)	Serbia, Krnjevo, 13.10.2022, N44 25.985 E21 1.927, lgt. Toševski	Serbia	*Crataegus nigra*	oxy34_COI (PV910530)	**2**
6412	*R. oxyacanthae* (larva L3)	Serbia, Krnjevo, 13.10.2022, N44 25.985 E21 1.927, lgt. Toševski	Serbia	*Crataegus monogyna*		
6417	*R. oxyacanthae* (larva L3)	Serbia, Staničenje, 15.09.2022, N43 13.020 E22 30.556, 403 m, lgt. Toševski	Serbia	*Crataegus monogyna*	oxy35_COI (PV910531)	**1**
6418	*R. oxyacanthae* (larva L3)	Serbia, Staničenje, 15.09.2022, N43 13.020 E22 30.556, 403 m, lgt. Toševski	Serbia	*Pyrus spinosa*	oxy36_COI (PV910532)	**1**
6407	*R. oxyacanthae*	Bulgaria, Sinemorets, 11.07.2021, N42 03.036 E27 58.504, 15 m., lgt. Toševski	Bulgaria	*Crataegus monogyna*	oxy37_COI (PV910532)	**1**
6408	*R. oxyacanthae*	Bulgaria, Sinemorets, 11.07.2021, N42 03.036 E27 58.504, 15 m., lgt. Toševski	Bulgaria	*Crataegus monogyna*	oxy38_COI (PV910534)	**1**
6406	*R. oxyacanthae*	Bulgaria, Sinemorets, 11.07.2021, N42 03.036 E27 58.504, 15 m., lgt. Toševski	Bulgaria	*Crataegus monogyna*	oxy39_COI (PV910535)	**1**
6405	*R. oxyacanthae*	Bulgaria, Sinemorets, 11.07.2021, N42 03.036 E27 58.504, 15 m.,	Bulgaria	*Crataegus monogyna*	oxy40_COI (PV910536)	**1**

		lgt. Toševski				
6402	*R. oxyacanthae* (larva L3)	Serbia, Krnjevo, 13.10.2022, N44 25.985 E21 1.927, ex larva L3, lgt. Toševski	Serbia	*Crataegus monogyna*	oxy41_COI (PV910537)	**1**
						**65**

[a] Diotti, L., Caldara, R., & Toševski, I. (2021). Description of two new species of *Rhamphus* related to *R. oxyacanthae* (Curculionidae, Curculioninae, Rhamphini) from Italy based on a morphological study supported by molecular data. *Zootaxa*, 4995(1), 111-128.
[b] Caldara, R., Toevski, I., Mendel, H., & Germann, C. (2022). In search of some type-specimens of *Rhamphus* [Clairville], 1798 (Coleoptera: Curculionidae). *Zootaxa*, *5169* (4), 371-380.

Rhamphus bavierai Diotti, Caldara & Toševski, 2021

**Voucher DNA**	**Species**	**Location**	**Origin**	**Host**	**COI haplotype name (accession number)**	**Frequency**
5967	*R. bavierai* HOLOTYPE	Sicily, Catania: Etna 1200 m., Linguaglossa, 27.06.2020, lgt. Baviera C.	Sicily	*Crataegus monogyna, C. laciniata*	bav1_COI (MW879286)[a]	**1**
5966	*R. bavierai*	Sicily, Catania: Etna 1200 m., Linguaglossa, 27.06.2020, lgt. Baviera C.	Sicily	*Crataegus monogyna, C. laciniata*	bav2_COI (MW879287)[a]	**2**
5970	*R. bavierai*	Sicily, Catania: Etna 1200 m., Linguaglossa, 27.06.2020, lgt. Baviera C.	Sicily	*Crataegus monogyna, C. laciniata*		
5984	*R. bavierai*	Sicily, Messina: Nebrodi Caronia, 1400 m., 20.06.2020, lgt. Baviera C.	Sicily	*Crataegus monogyna, C. laciniata*	bav3_COI (MW879288)[a]	**2**
5986	*R. bavierai*	Sicily, Messina: Nebrodi Caronia, 1400 m., 20.06.2020, lgt. Baviera C.	Sicily	*Crataegus monogyna, C. laciniata*		
5975	*R. bavierai*	Sicily, Messina: Nebrodi Caronia, 1400 m., 20.06.2020, lgt. Baviera C.	Sicily	*Crataegus monogyna, C. laciniata*	bav4_COI (MW879289)[a]	**1**
5973	*R. bavierai*	Sicily, Messina: Nebrodi Caronia, 1400 m., 20.06.2020, lgt. Baviera C.	Sicily	*Crataegus monogyna, C. laciniata*	bav5_COI (MW879290)[a]	**10**
5974	*R. bavierai*	Sicily, Messina: Nebrodi Caronia, 1400 m., 20.06.2020, lgt. Baviera C.	Sicily	*Crataegus monogyna, C. laciniata*		
5978	*R. bavierai*	Sicily, Messina: Nebrodi Caronia, 1400 m., 20.06.2020, lgt. Baviera C.	Sicily	*Crataegus monogyna, C. laciniata*		
5977	*R. bavierai*	Sicily, Messina: Nebrodi Caronia, 1400 m., 20.06.2020, lgt. Baviera C.	Sicily	*Crataegus monogyna, C. laciniata*		
5979	*R. bavierai*	Sicily, Messina: Nebrodi Caronia, 1400 m.,	Sicily	*Crataegus monogyna,*		

		20.06.2020, lgt. Baviera C.		*C. laciniata*		
5980	*R. bavierai*	Sicily, Messina: Nebrodi Caronia, 1400 m., 20.06.2020, lgt. Baviera C.	Sicily	*Crataegus monogyna, C. laciniata*		
5981	*R. bavierai*	Sicily, Messina: Nebrodi Caronia, 1400 m., 20.06.2020, lgt. Baviera C.	Sicily	*Crataegus monogyna, C. laciniata*		
5982	*R. bavierai*	Sicily, Messina: Nebrodi Caronia, 1400 m., 20.06.2020, lgt. Baviera	Sicily	*Crataegus monogyna, C. laciniata*		
5983	*R. bavierai*	Sicily, Messina: Nebrodi Caronia, 1400 m., 20.06.2020, lgt. Baviera C.	Sicily	*Crataegus monogyna, C. laciniata*		
5989	*R. bavierai*	Sicily, Messina: Nebrodi, Capizzi, 1450 m, Portella Obolo, 20.06.2020, lgt. Baviera C.	Sicily	*Crataegus monogyna, C. laciniata*		
5988	*R. bavierai*	Sicily, Messina: Nebrodi, Capizzi, 1450 m, Portella Obolo, 20.06.2020, lgt. Baviera C.	Sicily	*Crataegus monogyna, C. laciniata*	bav6_COI (MW879291)[a]	**1**
5965	*R. bavierai*	Sicily, Palermo: Madonie, P. zucchi, 1100 m a.s.l., 16.06.2020, lgt. Baviera C.	Sicily	*Pyrus* sp.	bav7_COI (MW879292)[a]	**2**
5985	*R. bavierai*	Sicily, Messina: Nebrodi Caronia, 1400 m., 20.06.2020, lgt. Baviera C.	Sicily	*Crataegus monogyna, C. laciniata*		
5976	*R. bavierai*	Sicily, Messina: Nebrodi Caronia, 1400 m., 20.06.2020, lgt. Baviera C.	Sicily	*Crataegus monogyna, C. laciniata*	bav8_COI (MW879293)[a]	**1**
5987	*R. bavierai*	Sicily, Messina: Nebrodi, Capizzi, 1450 m, Portella Obolo, 20.06.2020 lgt. Baviera C.	Sicily	*Crataegus monogyna, C. laciniata*	bav9_COI (MW879294)[a]	**1**
4406	*R. bavierai*	Sicily, Piano Battaglia, 1600 m., 15.07.2016, lgt Baviera C.	Sicily	*Crataegus monogyna, C. laciniata*	bav10_COI (MW879295)[a]	**1**
4404	*R. bavierai*	Sicily, Piano Battaglia, 1600 m., 15.07.2016, lgt Baviera C.	Sicily	*Crataegus monogyna, C. laciniata*	bav11_COI (MW879296)[a]	**3**
4405	*R. bavierai*	Sicily, Piano Battaglia, 1600 m, 15.07.2016, lgt Baviera C.	Sicily	*Crataegus monogyna, C. laciniata*		
4407	*R. bavierai*	Sicily, Piano Battaglia, 1600 m., 15.07.2016, lgt Baviera C.	Sicily	*Crataegus monogyna, C. laciniata*		

5968	*R. bavierai*	Sicily, Catania: Etna 1200 m., Linguaglossa, 27.06.2020 lgt. Baviera C.	Sicily	*Crataegus monogyna, C. laciniata*	bav12_COI (MW879297)[a]	**1**
5972	*R. bavierai*	Sicily, Catania: Etna 1200 m., Linguaglossa, 27.06.2020 lgt. Baviera C.	Sicily	*Crataegus monogyna, C. laciniata*	bav13_COI (MW879298)[a]	**1**
5969	*R. bavierai*	Sicily, Catania: Etna 1200 m., Linguaglossa, 27.06.2020 lgt. Baviera C.	Sicily	*Crataegus monogyna, C. laciniata*	bav14_COI (MW879299)[a]	**1**
5971	*R. bavierai*	Sicily, Catania: Etna 1200 m., Linguaglossa, 27.06.2020 lgt. Baviera C.	Sicily	*Crataegus monogyna, C. laciniata*	bav15_COI (MW879300)[a]	**1**
5964	*R. bavierai*	Sicily, Palermo: Madonie, P. Zucchi, 1100 m a.s.l., 16.06.2020, lgt. Baviera	Sicily	*Pyrus* sp.	bav16_COI (MW879301)[a]	**1**
5960	*R. bavierai*	Sicily, Palermo: Madonie, P. Zucchi, 1100 m a.s.l., 16.06.2020, lgt. Baviera C	Sicily	*Pyrus* sp.	bav17_COI (MW879302)[a]	**2**
5961	*R. bavierai*	Sicily, Palermo: Madonie, P. Zucchi, 1100 m a.s.l., 16.06.2020, lgt. Baviera C.	Sicily	*Pyrus* sp.		
4403	*R. bavierai*	Sicily, Piano Battaglia, 1600 m., 15.07.2016, lgt. Baviera C.	Sicily	*Crataegus monogyna, C. laciniata*	bav18_COI (MW879303)[a]	**1**
6004	*R. bavierai*	Sicily, Piano Battaglia, 1600 m., 15.07.2016, Lgt. Baviera C.	Sicily	*Crataegus monogyna, C. laciniata*	bav19_COI (PV910599)	**1**
6005	*R. bavierai*	Sicily, Piano Battaglia, 1600 m., 15.07.2016, Lgt. Baviera C.	Sicily	*Crataegus monogyna, C. laciniata*	bav20_COI (PV910600)	**1**
						**35**

[a] Diotti, L., Caldara, R., & Toševski, I. (2021). Description of two new species of *Rhamphus* related to *R. oxyacanthae* (Curculionidae, Curculioninae, Rhamphini) from Italy based on a morphological study supported by molecular data. *Zootaxa*, 4995(1), 111-128.

Rhamphus hampsicora **Diotti, Caldara & Toševski, 2021**

**Voucher DNA**	**Species**	**Location**	**Origin**	**Host**	**COI haplotype name (accession number)**	**Frequency**
5341	*R. hampsicora*	Sardinia, Siniscola (NU), Pandici Monte Albo, 25.05.2012, lgt. Diotti	Sardinia	*Prunus* sp.	ham1_COI (MW879304)[a]	**1**
5340	*R. hampsicora*	Sardinia, Siniscola (NU), Pandici Monte Albo, 25.05.2012, lgt. Diotti	Sardinia	*Prunus* sp.	ham2_COI (MW879305)[a]	**1**
5339	*R. hampsicora*	Sardinia, Siniscola (NU), Pandici Monte Albo, 25.05.2012, lgt. Diotti	Sardinia	*Prunus* sp.	ham3_COI (MW879306)[a]	**2**

5342	*R. hampsicora*	Sardinia, Siniscola (NU), Pandici Monte Albo, 25.05.2012, lgt. Diotti	Sardinia	*Prunus* sp.		
						**4**

[a] Diotti, L., Caldara, R., & Toševski, I. (2021). Description of two new species of *Rhamphus* related to *R. oxyacanthae* (Curculionidae, Curculioninae, Rhamphini) from Italy based on a morphological study supported by molecular data. *Zootaxa*, 4995(1), 111-128.

Rhamphus cypricus sp. n. Toševski & Caldara

Voucher DNA	Species	Location	Origin	Host	COI haplotype name (accession number)	Frequency
6178	*R. cypricus* sp.n. PARATYPE	Cyprus, Paphos p., Drouseia env., 541 m. 23.03.2019, lgt. F. Pavel	Cyprus	no data	cyp1_COI (PV910601)	**6**
7374	*R. cypricus* sp.n. PARATYPE	Cyprus, Parsata, 7.04.2024, 34.8281745 N, 33.2648008 E, lgt. L. Forbicioni	Cyprus	*Crataegus azarolus*		
7377	*R. cypricus* sp.n. PARATYPE	Cyprus, Parsata, 7.04.2024, 34.8281745 N, 33.2648008 E, lgt. L. Forbicioni	Cyprus	*Crataegus azarolus*		
7378	*R. cypricus* sp.n. PARATYPE	Cyprus, Parsata, 7.04.2024, 34.8281745 N, 33.2648008 E, lgt. L. Forbicioni	Cyprus	*Crataegus azarolus*		
7379	*R. cypricus* sp.n. PARATYPE	Cyprus, Lefkara, 12.04.2024, 34.8692177 N, 33.2839350 E, lgt. L. Forbicioni	Cyprus	*Crataegus azarolus*		
7380	*R. cypricus* sp.n. PARATYPE	Cyprus, Lefkara, 12.04.2024, 34.8692177 N, 33.2839350 E, lgt. L. Forbicioni	Cyprus	*Crataegus azarolus*		
7372	*R. cypricus* sp.n. **HOLOTYPE**	Cyprus, Parsata, 7.04.2024, 34.8281745 N, 33.2648008 E, lgt. L. Forbicioni	Cyprus	*Crataegus azarolus*	cyp2_COI (PV910538)	**1**
7373	*R. cypricus* sp.n. PARATYPE	Cyprus, Parsata, 7.04.2024, 34.8281745 N, 33.2648008 E, lgt. L. Forbicioni	Cyprus	*Crataegus azarolus*	cyp3_COI (PV910539)	**1**
7371	*R. cypricus* sp.n. PARATYPE	Cyprus, Parsata, 7.04.2024, 34.8281745 N, 33.2648008 E, lgt. L. Forbicioni	Cyprus	*Crataegus azarolus*	cyp4_COI (PV910540)	**2**
7376	*R. cypricus* sp.n. PARATYPE	Cyprus, Parsata, 7.04.2024, 34.8281745 N, 33.2648008 E, lgt. L. Forbicioni	Cyprus	*Crataegus azarolus*		
7375	*R. cypricus* sp.n. PARATYPE	Cyprus, Parsata, 7.04.2024, 34.8281745 N, 33.2648008 E, lgt. L. Forbicioni	Cyprus	*Crataegus azarolus*	cyp5_COI (PV910541)	**1**
7346	*R. cypricus* sp.n. PARATYPE	Cyprus, Droushia-Ineia(Paphos, Chypre) 600m, 12.04.2005, G. et H. Alziar lgt. sur Crataegus	Cyprus	*Crataegus* sp.	-	**-**

		fleuri. Collection G. Alziar				
7349	*R. cypricus* sp.n. PARATYPE	Cyprus, Droushia-Ineia(Paphos, Chypre) 600m, 12.04.2005, G. et H. Alziar lgt. sur Crataegus fleuri. Collection G. Alziar	Cyprus	*Crataegus* sp.	-	**-**
						**11**

***Rhamphus macedonicus* sp. n. Toševski & Caldara**						
**Voucher DNA**	**Species**	**Location**	**Origin**	**Host**	**COI haplotype name (accession number)**	**Frequency**
6582	*R. macedonicus* sp.n. **HOLOTYPE**	Greece, Macedonia, Arethousa, 23.06.2023, N40 44.134 E23 36.193, 374 m. lgt. Toševski	Greece	*Crataegus* sp.	mac1_COI (PV910542)	**2**
6583	*R. macedonicus* sp.n. PARATYPE	Greece, Macedonia, Arethousa, 23.06.2023, N40 44.134 E23 36.193, 374 m. lgt. Toševski	Greece	*Crataegus* sp.		
6584	*R. macedonicus* sp.n. PARATYPE	Greece, Macedonia, Arethousa, 23.06.2023, N40 44.134 E23 36.193, 374 m. lgt. Toševski	Greece	*Crataegus* sp.	mac2_COI (PV910543)	**1**
7390	*R. macedonicus* sp.n. PARATYPE	Greece, Macedonia, Arethousa, 13.06.2024, N40 44.134 E23 36.193, 374 m. lgt. J. Jović	Greece	*Crataegus* sp.	mac3_COI (PV910544)	**1**
7385	*R. macedonicus* sp.n. PARATYPE	Greece, Macedonia, Arethousa, 13.06.2024, N40 44.134 E23 36.193, 374 m. lgt. J. Jović	Greece	*Crataegus* sp.	mac4_COI (PV910545)	**1**
7381	*R. macedonicus* sp.n. PARATYPE	Greece, Macedonia, Arethousa, 13.06.2024, N40 44.134 E23 36.193, 374 m. lgt. J. Jović	Greece	*Crataegus* sp.	mac5_COI (PV910546)	**6**
7383	*R. macedonicus* sp.n. PARATYPE	Greece, Macedonia, Arethousa, 13.06.2024, N40 44.134 E23 36.193, 374 m. lgt. J. Jović	Greece	*Crataegus* sp.		
7386	*R. macedonicus* sp.n. PARATYPE	Greece, Macedonia, Arethousa, 13.06.2024, N40 44.134 E23 36.193, 374 m. lgt. J. Jović	Greece	*Crataegus* sp.		
7389	*R. macedonicus* sp.n. PARATYPE	Greece, Macedonia, Arethousa, 13.06.2024, N40 44.134 E23 36.193, 374 m. lgt. J. Jović	Greece	*Crataegus* sp.		
7384	*R. macedonicus* sp.n. PARATYPE	Greece, Macedonia, Arethousa, 13.06.2024, N40 44.134 E23 36.193,	Greece	*Crataegus* sp.		

		374 m. lgt. J. Jović				
7393	*R. macedonicus* sp.n. PARATYPE	Greece, Macedonia, Arethousa, 13.06.2024, N40 44.134 E23 36.193, 374 m. lgt. J. Jović	Greece	*Crataegus* sp.		
7387	*R. macedonicus* sp.n. PARATYPE	Greece, Macedonia, Arethousa, 13.06.2024, N40 44.134 E23 36.193, 374 m. lgt. J. Jović	Greece	*Crataegus* sp.	mac6_COI (PV910547)	**3**
7388	*R. macedonicus* sp.n. PARATYPE	Greece, Macedonia, Arethousa, 13.06.2024, N40 44.134 E23 36.193, 374 m. lgt. J. Jović	Greece	*Crataegus* sp.		
7391	*R. macedonicus* sp.n. PARATYPE	Greece, Macedonia, Arethousa, 13.06.2024, N40 44.134 E23 36.193, 374 m. lgt. J. Jović	Greece	*Crataegus* sp.		
7392	*R. macedonicus* sp.n. PARATYPE	Greece, Macedonia, Arethousa, 13.06.2024, N40 44.134 E23 36.193, 374 m. lgt. J. Jović	Greece	*Crataegus* sp.	mac7_COI (PV910548)	**1**
						**15**

Rhamphus pulicarius **(Herbst, 1795)**

**Voucher DNA**	**Species**	**Location**	**Origin**	**Host**	**COI haplotype name (accession number)**	**Frequency**
4540	*R. pulicarius*	Serbia, Draglica, Mt. Zlatar, N43 35.173 E19 43.645, 929 m., 12.07.2017, lgt. Toševski	Serbia	*Salix caprea*	pul1_COI (PV910549)	**12**
4541	*R. pulicarius*	Serbia, Babin Zub, Stara Planina, N43 23.165 E22 35.549, 1250 m, 5.07.2017, lgt. Toševski	Serbia	*Salix caprea*		
4585	*R. pulicarius*	Serbia, Babin Zub, Stara Planina, N43 23.165 E22 35.549, 1250 m, 5.07.2017, lgt. Toševski	Serbia	*Salix caprea*		
5179	*R. pulicarius*	Italy, Lombardia (CO), dintorni Monguzzo, 7.06.2017, lgt. L. Diotti	Italy	no data		
6010	*R. pulicarius*	France, F 19, Chavanac, 6.07.2020, 8.07.2020, lgt. Lessieur David	France	*Salix sp.*		
6223	*R. pulicarius*	Poland, Rudnik ad Lublin, 51°16'58.8"N 22°38'28.8"E, 15.06.2021, lgt. Rafał Gosik	Poland	*Betula* sp.		
6226	*R. pulicarius*	Poland, Rudnik ad Lublin, 51°16'58.8"N 22°38'28.8"E, 15.06.2021, lgt. Rafał Gosik	Poland	*Salix* sp.		
6227	*R. pulicarius*	Serbia, Jokino Vrelo, Kremna, Mt. Tara	Serbia	*Salix alba*		

		24.06.2021, lgt. Toševski				
6228	*R. pulicarius*	Serbia, Jokino Vrelo, Kremna, Mt. Tara 24.06.2021, lgt. Toševski	Serbia	*Salix alba*		
6229	*R. pulicarius*	Serbia, Jokino Vrelo, Kremna, Mt. Tara 24.06.2021, lgt. Toševski	Serbia	*Salix alba*		
6351	*R. pulicarius*	England, 21/023, Lynford, Mundford, West Norfolk (VC28), TL8294, 16.06.2021, lgt. H. Mendel	England	*Salix* sp.		
6353	*R. pulicarius*	England, 21/023, Lynford, Mundford, West Norfolk (VC28), TL8294, 16.06.2021, lgt. H. Mendel	England	*Salix* sp.		
4579	*R. pulicarius*	Serbia, Draglica, Mt. Zlatar, N43 35.173 E19 43.645, 929 m., 12.07.2017, lgt. Toševski	Serbia	*Salix caprea*	pul2_COI (PV910550)	**2**
4583	*R. pulicarius*	Serbia, Babin Zub, Stara Planina, N43 23.165 E22 35.549, 1250 m, 5.07.2017, lgt. Toševski	Serbia	*Salix caprea*		
ZFMK-TIS-2550709	*R. pulicarius* (NEOTYPE)	Germany, Saxony-Anhalt, Halberstadt, Lkr. Harz, Freigelaende suedl. Klussiedlung, 10.06.2015, N51.8494 E11.0496 Alexander Koenig Mus. https://id.zfmk.de/collection_ZFMK/html/2550709/1051495/201827	Germany	no data	pul3_COI (KU909870)[C]	**1**
4580	*R. pulicarius*	Serbia, Draglica, Mt. Zlatar, N43 35.173 E19 43.645, 929 m., 12.07.2017, lgt. Toševski	Serbia	*Salix caprea*	pul4_COI (PV910551)	**1**
5180	*R. pulicarius*	Italy, Lombardia (CO), dintorni Monguzzo, 7.06.2017, lgt. L. Diotti	Italy	*Salix sp.*	pul5_COI (PV910602)	**4**
6392	*R. pulicarius*	Erba, Lago di Alserio, 17.07.2022, lgt. L. Diotti,	Italy	*Salix sp.*		
6393	*R. pulicarius*	Erba, Lago di Alserio, 17.07.2022, lgt. L. Diotti,	Italy	*Salix sp.*		
6394	*R. pulicarius*	Erba, Lago di Alserio, 17.07.2022, lgt. L. Diotti,	Italy	*Salix sp.*		
6011	*R. pulicarius*	France, F 19, Chavanac, 6.07.2020, 8.07.2020, on Salix, lgt. Lessieur David	France	*Salix sp.*	pul6_COI (PV910603)	**1**
6391	R. pulicarius	Erba, Lago di Alserio, 17.07.2022, lgt. L. Diotti	Italy	*Salix sp.*	pul7_COI (PV910604)	1
7442	*R. pulicarius*	Greece, Prodromis, mined leave of *Salix* sp., GPS 006 N40 27.650 E23 23.248, 28.09.2024, lgt. Toševski	Greece	*Salix sp.*	pul8_COI (PV910552)	**6**

7443	*R. pulicarius*	Greece, Prodromis, mined leave of *Salix* sp., GPS 006 N40 27.650 E23 23.248, 28.09.2024, lgt. Toševski	Greece	*Salix sp.*		
7444	*R. pulicarius*	Greece, Prodromis, mined leave of *Salix* sp., GPS 006 N40 27.650 E23 23.248, 28.09.2024, lgt. Toševski	Greece	*Salix sp.*		
7445	*R. pulicarius*	Greece, Prodromis, mined leave of *Salix* sp., GPS 006 N40 27.650 E23 23.248, 28.09.2024, lgt. Toševski	Greece	*Salix sp.*		
7446	*R. pulicarius*	Greece, Prodromis, mined leave of *Salix* sp., GPS 006 N40 27.650 E23 23.248, 28.09.2024, lgt. Toševski	Greece	*Salix sp.*		
7447	*R. pulicarius*	Greece, Prodromis, mined leave of *Salix* sp., GPS 006 N40 27.650 E23 23.248, 28.09.2024, lgt. Toševski	Greece	*Salix sp.*		
						**28**

[c] Rulik, B., Eberle, J., von der Mark, L., Thormann, J., Jung, M., Köhler, F., Apfel, W., Weigel, A., Kopetz, A., Köhler, J. and Fritzlar, F., 2017. Using taxonomic consistency with semi-automated data pre-processing for high quality DNA barcodes. *Methods in Ecology and Evolution*, *8*(12), pp.1878-1887.

Rhamphus pullus **Hustache, 1920**

**Voucher DNA**	**Species**	**Location**	**Origin**	**Host**	**COI haplotype name (accession number)**	**Frequency**
6334	*R. pullus*	Japan, Mikuni pass., Yamanakako vlg., Yamanashi pref., 12.06.2021, lgt. Y. Notsu	Japan	*Betula* sp.	pull1_COI (PV910605)	**1**
6336	*R. pullus*	Japan, Mikuni pass., Yamanakako vlg., Yamanashi pref., 12.06.2021, lgt. Y. Notsu	Japan	*Betula* sp.	pull2_COI (PV910606)	**2**
6337	*R. pullus*	Japan, Mikuni pass., Yamanakako vlg., Yamanashi pref., 12.06.2021, lgt. Y. Notsu	Japan	*Betula* sp.		
						**3**

Rhamphus betulae **sp. n. Toševski & Caldara**

**Voucher DNA**	**Species**	**Location**	**Origin**	**Host**	**COI haplotype name (accession number)**	**Frequency**
6216	*R. betulae* sp.n. PARATYPE	Poland, Rudnik ad Lublin 51°16'58.8"N 22°38'28.8"E, 15.06.2021, lgt. Rafal Gosik	Poland	*Betula* sp.	bet1_COI (PV910553)	**1**

6217	*R. betulae* sp.n. PARATYPE	Poland, Rudnik ad Lublin 51°16'58.8"N 22°38'28.8"E, 15.06.2021, lgt. Rafal Gosik	Poland	*Betula* sp.	bet2_COI (PV910554)	**9**
6218	*R. betulae* sp.n. PARATYPE	Poland, Rudnik ad Lublin 51°16'58.8"N 22°38'28.8"E, 15.06.2021, lgt. Rafal Gosik	Poland	*Betula* sp.		
6219	*R. betulae* sp.n. PARATYPE	Poland, Rudnik ad Lublin 51°16'58.8"N 22°38'28.8"E, 15.06.2021, lgt. Rafal Gosik	Poland	*Betula* sp.		
6220	*R. betulae* sp.n. PARATYPE	Poland, Rudnik ad Lublin 51°16'58.8"N 22°38'28.8"E, 15.06.2021, lgt. Rafal Gosik	Poland	*Betula* sp.		
6221	*R. betulae* sp.n. PARATYPE	Poland, Rudnik ad Lublin 51°16'58.8"N 22°38'28.8"E, 15.06.2021, lgt. Rafal Gosik	Poland	*Betula* sp.		
6222	*R. betulae* sp.n. PARATYPE	Poland, Rudnik ad Lublin 51°16'58.8"N 22°38'28.8"E, 15.06.2021, lgt. Rafal Gosik	Poland	*Betula* sp.		
6224	*R. betulae* sp.n. PARATYPE	Poland, Rudnik ad Lublin 51°16'58.8"N 22°38'28.8"E, 15.06.2021, lgt. Rafal Gosik	Poland	*Betula* sp.		
6225	*R. betulae* sp.n. PARATYPE	Poland, Rudnik ad Lublin 51°16'58.8"N 22°38'28.8"E, 15.06.2021, lgt. Rafal Gosik	Poland	*Betula* sp.		
6322	*R. betulae* sp.n. PARATYPE	Italy, Val Sesia, (VC) Monte Tovo, 1100 m, 12.06,2021, lgt. L. Diotti	Italy	*Betula pendula*		
6315	*R. betulae* sp.n. PARATYPE	Italy, Val Sesia, (VC) Monte Tovo, 1100 m, 12.06,2021, lgt. L. Diotti	Italy	*Betula pendula*	bet3_COI (PV910607)	**7**
6316	*R. betulae* sp.n. **HOLOTYPE**	Italy, Val Sesia, (VC) Monte Tovo, 1100 m, 12.06,2021, lgt. L. Diotti	Italy	*Betula pendula*		
6317	*R. betulae* sp.n. PARATYPE	Italy, Val Sesia, (VC) Monte Tovo, 1100 m, 12.06,2021, lgt. L. Diotti	Italy	*Betula pendula*		
6318	*R. betulae* sp.n. PARATYPE	Italy, Val Sesia, (VC) Monte Tovo, 1100 m, 12.06,2021, lgt. L. Diotti	Italy	*Betula pendula*		
6319	*R. betulae* sp.n. PARATYPE	Italy, Val Sesia, (VC) Monte Tovo, 1100 m, 12.06,2021, lgt. L. Diotti	Italy	*Betula pendula*		
6320	*R. betulae* sp.n. PARATYPE	Italy, Val Sesia, (VC) Monte Tovo, 1100 m, 12.06,2021, lgt. L. Diotti	Italy	*Betula pendula*		
6321	*R. betulae* sp.n. PARATYPE	Italy, Val Sesia, (VC) Monte Tovo, 1100 m, 12.06,2021, lgt. L. Diotti	Italy	*Betula pendula*		
6606	*R. betulae* sp.n. PARATYPE	France, 65 Trébons, 43.0799872049 /0.113917399, 30.06.2023 bouhaben, lgt. D. Lessieur	France	*Betula* sp.	bet4_COI (PV910555)	**8**
6608	*R. betulae* sp.n. PARATYPE	France, 65 Trébons, 43.0799872049	France	*Betula* sp.		

		/0.113917399, 30.06.2023 bouhaben, lgt. D. Lessieur				
6609	*R. betulae* sp.n. PARATYPE	France, 65 Trébons, 43.0799872049 /0.113917399, 30.06.2023 bouhaben, lgt. D. Lessieur	France	*Betula* sp.		
6610	*R. betulae* sp.n. PARATYPE	France, 65 Trébons, 43.0799872049 /0.113917399, 30.06.2023 bouhaben, lgt. D. Lessieur	France	*Betula* sp.		
6611	*R. betulae* sp.n. PARATYPE	France, 65 Trébons, 43.0799872049 /0.113917399, 30.06.2023 bouhaben, lgt. D. Lessieur	France	*Betula* sp.		
6612	*R. betulae* sp.n. PARATYPE	France, 65 Trébons, 43.0799872049 /0.113917399, 30.06.2023 bouhaben, lgt. D. Lessieur	France	*Betula* sp.		
6613	*R. betulae* sp.n. PARATYPE	France - 65 Pouzac, 43.0758181239 /0.116744447, 2.07.2023, gardeloup, lgt. D. Lessieur	France	*Betula* sp.		
6614	*R. betulae* sp.n. PARATYPE	France - 65 Pouzac, 43.0758181239 /0.116744447, 2.07.2023, gardeloup, lgt. D. Lessieur	France	*Betula* sp.		
6607	*R. betulae* sp.n. PARATYPE	France, 65 Trébons, 43.0799872049 /0.113917399, 30.06.2023 bouhaben, lgt. D. Lessieur	France	*Betula* sp.	bet5_COI (PV910556)	**1**
						**26**

Rhamphus crypticus **sp. n. Toševski & Caldara**

**Voucher DNA**	**Species**	**Location**	**Origin**	**Host**	**COI haplotype name (accession number)**	**Frequency**
6356	*R. crypticus* sp.n. PARATYPE	England, 21/026, Denge Beach, East Kent (VC15), TLR0817, 28.06.2021, lgt. H. Mendel	England	*Salix* sp.	cry1_COI (PV910608)	**6**
6357	*R. crypticus* sp.n. PARATYPE	England, 21/026, Denge Beach, East Kent (VC15), TLR0817, 28.06.2021, lgt. H. Mendel	England	*Salix* sp.		
6358	*R. crypticus* sp.n. PARATYPE	England, 21/026, Denge Beach, East Kent (VC15), TLR0817, 28.06.2021, lgt. H. Mendel	England	*Salix* sp.		

6359	*R. crypticus* sp.n. PARATYPE	England, 21/026, Denge Beach, East Kent (VC15), TLR0817, 28.06.2021, lgt. H. Mendel	England	*Salix* sp.		
6360	*R. crypticus* sp.n. PARATYPE	England, 21/026, Denge Beach, East Kent (VC15), TLR0817, 28.06.2021, lgt. H. Mendel	England	*Salix* sp.		
6352	*R. crypticus* sp.n. **HOLOTYPE**	England, 21/023, Lynford, Mundford, West Norfolk (VC28), TL8294, lgt. H. Mendel	England	*Salix* sp.		
6354	*R. crypticus* sp.n. PARATYPE	England, 21/026, Denge Beach, East Kent (VC15), TLR0817 28.06.2021, lgt. H. Mendel	England	*Salix* sp.	cry2_COI (PV910609)	**2**
6355	*R. crypticus* sp.n. PARATYPE	England, 21/026, Denge Beach, East Kent (VC15), TLR0817 28.06.2021, lgt. H. Mendel	England	*Salix* sp.		
6006	*R. crypticus* sp.n. PARATYPE	England, New Forest, S. Hants, SU2404, 13.07.2020, lgt. H. Mendel	England	*Salix* sp.	cry3_COI (PV910610)	**1**
6007	*R. crypticus* sp.n. PARATYPE	England, New Forest, S. Hants, SU2404, 13.07.2020, lgt. H. Mendel	England	*Salix* sp.	cry4_COI (PV910611)	**1**
6008	*R. crypticus* sp.n. PARATYPE	England, New Forest, S. Hants, SU2404, 13.07.2020, lgt. H. Mendel	England	*Salix* sp.	cry5_COI (PV910612)	**1**
6009	*R. crypticus* sp.n. PARATYPE	England, New Forest, S. Hants, SU2404, 13.07.2020, lgt. H. Mendel	England	*Salix* sp.	cry6_COI (PV910613)	**1**
6018	*R. crypticus* sp.n. PARATYPE	France, F 19, La Tronche (19110), la croix de Layre, 16.VII.2020, lgt. D. Lessieur	France	no data	cry7_COI (PV910614)	**2**
6618	*R. crypticus* sp.n. PARATYPE	France, Hautes-Pyrénées (65) - Poueyferré, 43.112498397 /-0.093337389, 29.06.2023, Tourbiére de Lourdes, lgt. D. Lessieur	France	*Salix atrocinerea*		
6625	*R. crypticus* sp.n. PARATYPE	France - Hautes-Pyrénées (65) Esparros, 43.0262449 /0.280096, 2.07.2023, lgt. D. Lessieur	France	*Salix atrocinerea*	cry8_COI (PV910557)	**1**
6623	*R. crypticus* sp.n. PARATYPE	France - Hautes-Pyrénées (65) Banios, 43.0409039 /0.233232, 1.07.2023, lgt. D. Lessieur	France	*Salix atrocinerea*	cry9_COI (PV910558)	**1**

6615	*R. crypticus* sp.n. PARATYPE	France, Hautes-Pyrénées (65) - Poueyferré, 43.112498397 /-0.093337389, 29.06.2023, Tourbiére de Lourdes, lgt. D. Lessieur	France	*Salix atrocinerea*	cry10_COI (PV910559)	**7**
6616	*R. crypticus* sp.n. PARATYPE	France, Hautes-Pyrénées (65) - Poueyferré, 43.112498397 /-0.093337389, 29.06.2023, Tourbiére de Lourdes, lgt. D. Lessieur	France	*Salix atrocinerea*		
6617	*R. crypticus* sp.n. PARATYPE	France, Hautes-Pyrénées (65) - Poueyferré, 43.112498397 /-0.093337389, 29.06.2023, Tourbiére de Lourdes, lgt. D. Lessieur	France	*Salix atrocinerea*		
6619	*R. crypticus* sp.n. PARATYPE	France, Hautes-Pyrénées (65) Pouzac, 43.076836910 /0.119550038, 28.06.2023, gardeloup, lgt. D. Lessieur	France	*Salix atrocinerea*		
6620	*R. crypticus* sp.n. PARATYPE	France, Hautes-Pyrénées (65) Pouzac, 43.076836910 /0.119550038, 28.06.2023, gardeloup, lgt. D. Lessieur	France	*Salix atrocinerea*		
6621	*R. crypticus* sp.n. PARATYPE	France, Hautes-Pyrénées (65) Pouzac, 43.076836910 /0.119550038, 28.06.2023, gardeloup, lgt. D. Lessieur	France	*Salix atrocinerea*		
6624	*R. crypticus* sp.n. PARATYPE	France, Hautes-Pyrénées (65) Banios, 43.0409039 /0.233232, 1.07.2023, lgt. D. Lessieur	France	*Salix atrocinerea*		
6599	*R. crypticus* sp.n. PARATYPE	Spain, provincia de Soria, Vinuesa, río Revinuesa, 13-VII-2020, lgt. Iñigo Ugarte San Vicente & Fernando Salgueira	Spain	*Salix salviifolia*	cry11_COI (PV910615)	**1**
6600	*R. crypticus* sp.n. PARATYPE	Spain, provincia de Soria, Vinuesa, río Revinuesa, 13-VII-2020, lgt. Iñigo Ugarte San Vicente & Fernando Salgueira	Spain	*Salix salviifolia*	cry12_COI (PV910616)	**1**
						**25**

Rhamphus monzinii Pesarini & Diotti, 2012						
**Voucher DNA**	**Species**	**Location**	**Origin**	**Host**	**COI haplotype name (accession number)**	**Frequency**
5174	*R. monzinii*	Italy, Liguria, (GE), Piani di Creto, 23.06.2018 lgt. L. Diotti	Italy	no data	mon1_COI (MW879307)[a]	**1**
5175	*R. monzinii*	Italy, Liguria, (GE), Piani di Creto, 23.06.2018 lgt. L. Diotti	Italy	no data	mon2_COI (MW879308)[a]	**8**
5176	*R. monzinii*	Italy, Liguria, (GE), Piani di Creto, 23.06.2018 lgt. L. Diotti	Italy	no data		
5997	*R. monzinii*	Italy, Liguria, (GE), Piani di Creto, 23.06.2018 lgt. L. Diotti	Italy	no data		
5998	*R. monzinii*	Italy, Liguria, (GE), Piani di Creto, 23.06.2018 lgt. L. Diotti	Italy	no data		
6000	*R. monzinii*	Italy, Liguria, (GE), Piani di Creto, 23.06.2018 lgt. L. Diotti	Italy	no data		
6001	*R. monzinii*	Italy, Liguria, (GE), Piani di Creto, 23.06.2018 lgt. L. Diotti	Italy	no data		
6002	*R. monzinii*	Italy, Liguria, (GE), Piani di Creto, 23.06.2018 lgt. L. Diotti	Italy	no data		
6003	*R. monzinii*	Italy, Liguria, (GE), Piani di Creto, 23.06.2018 lgt. L. Diotti	Italy	no data		
5999	*R. monzinii*	Italy, Liguria, (GE), Piani di Creto, 23.06.2018, lgt. L. Diotti	Italy	no data	mon3_COI (PV910617)	**1**
6400	*R. monzinii* (larva L3)	Serbia, Krnjevo, Radovanovic, 13.10.2022, N44 25.985 E21 1.927, ex larva L3, lgt. Toševski	Serbia	*Prunus cerasifera*	mon4_COI (PV910560)	**21**
6409	*R. monzinii* (larva L3)	Serbia, Krnjevo, Radovanovic, 13.10.2022, N44 25.985 E21 1.927, ex larva L3, lgt. Toševski	Serbia	*Prunus cerasifera*		
6413	*R. monzinii* (larva L3)	Serbia, Krnjevo, Radovanovic, 13.10.2022, N44 25.985 E21 1.927, ex larva L3, lgt. Toševski	Serbia	*Prunus cerasifera*		
6414	*R. monzinii* (larva L3)	Serbia, Krnjevo, Radovanovic, 13.10.2022,	Serbia	*Prunus*		

		N44 25.985 E21 1.927, ex larva L3, lgt. Toševski		*cerasifera*		
4539	*R. monzinii*	Serbia, Mt. Zlatibor, 9.07.2017, N43 47.310 E19 43.721, 662 m, lgt. Toševski	Serbia	*Prunus cerasifera*		
4863	*R. monzinii*	Serbia, Pirot, Staničenje, 20.05.2018, N43 13.020 E22 30.556, 403 m, lgt. Toševski	Serbia	*Prunus spinosa*		
4883	*R. monzinii*	Serbia, Brusnik, Negotin, N44 6.489 E22 24.115, 322 m, 21.05.2018, lgt. Toševski	Serbia	*Prunus spinosa*		
4887	*R. monzinii*	Serbia, Brusnik, Negotin, N44 6.489 E22 24.115, 322 m, 21.05.2018, lgt. Toševski	Serbia	*Prunus spinosa*		
4888	*R. monzinii*	Serbia, Brusnik, Negotin, N44 6.489 E22 24.115, 322 m, 21.05.2018, lgt. Toševski	Serbia	*Prunus spinosa*		
4894	*R. monzinii*	Serbia, Vlasina, Božićki Kanal, N42 40.997 E22 21.888, 1289 m, 21.06.2018, lgt. Toševski	Serbia	*Prunus cerasifera*		
4898	*R. monzinii*	Serbia, Vranje, Devotin, N42 36.511 E21 52.252, 991 m, 22.06.2018, lgt. Toševski	Serbia	*Prunus spinosa*		
4899	*R. monzinii*	Serbia, Vranje, Devotin, N42 36.511 E21 52.252, 991 m, 22.06.2018, lgt. Toševski	Serbia	*Prunus spinosa*		
4900	*R. monzinii*	Serbia, Vranje, Devotin, N42 36.511 E21 52.252, 991 m, 22.06.2018, lgt. Toševski	Serbia	*Prunus spinosa*		
4897	*R. monzinii*	Serbia, Vranje, Devotin, N42 36.511 E21 52.252, 991 m, 22.06.2018, lgt. Toševski	Serbia	*Prunus spinosa*		
7067	*R. monzinii* (larva L3)	Serbia, Kostol, Kladovo, 17.11.2023 N44 36.533 E22 38.028, 79 m, lgt. Toševski	Serbia	*Prunus cerasifera*		
7069	*R. monzinii* (larva L3)	Serbia, Kostol, Kladovo, 17.11.2023 N44 36.533 E22 38.028, 79 m, lgt. Toševski	Serbia	*Prunus cerasifera*		
7071	*R. monzinii*	Serbia, Velika Vrbica,	Serbia	*Prunus*		

	(larva L3)	Kladovo, 17.11.2023 N44 36.072 E22 41.557, 59 m., lgt. Toševski		*cerasifera*		
7075	*R. monzinii* (larva L3)	Serbia, Petrovo Selo, 17.11.2023, N44 37.732 E22 31.321, 217 m., lgt. Toševski	Serbia	*Prunus cerasifera*		
7079	*R. monzinii* (larva L3)	Serbia, Petrovo Selo, 17.11.2023, N44 37.732 E22 31.321, 217 m., lgt. Toševski	Serbia	*Prunus spinosa*		
7080	*R. monzinii* (larva L3)	Serbia, Petrovo Selo, 17.11.2023, N44 37.732 E22 31.321, 217 m., lgt. Toševski	Serbia	*Prunus spinosa*		
7081	*R. monzinii* (larva L3)	Serbia, Petrovo Selo, 17.11.2023, N44 37.732 E22 31.321, 217 m., lgt. Toševski	Serbia	*Prunus spinosa*		
4858	*R. monzinii*	Serbia, Brusnik, Negotin, N44 6.489 E22 24.115, 322 m, 21.05.2018, lgt. Toševski	Serbia	*Cydonia oblonga*	mon5_COI (PV910561)	**17**
4862	*R. monzinii*	Serbia, Brusnik, Negotin, N44 6.489 E22 24.115, 322 m, 21.05.2018, lgt. Toševski	Serbia	*Prunus spinosa*		
4884	*R. monzinii*	Serbia, Brusnik, Negotin, N44 6.489 E22 24.115, 322 m, 21.05.2018, lgt. Toševski	Serbia	*Prunus spinosa*		
4885	*R. monzinii*	Serbia, Brusnik, Negotin, N44 6.489 E22 24.115, 322 m., 21.05.2018, lgt. Toševski	Serbia	*Prunus spinosa*		
4886	*R. monzinii*	Serbia, Brusnik, Negotin, N44 6.489 E22 24.115, 322 m, 21.05.2018, lgt. Toševski	Serbia	*Prunus spinosa*		
4890	*R. monzinii*	Serbia, Brusnik, Negotin, N44 6.489 E22 24.115, 322 m, 21.05.2018, lgt. Toševski	Serbia	*Prunus spinosa*		
6605	*R. monzinii* (larva L2)	Serbia, Krnjevo, 14.07.2023, N44 25.985 E21 1.927, ex larva L2, lgt. Toševski	Serbia	*Prunus cerasifera*		
7064	*R. monzinii* (larva L3)	Serbia, Kostol, Kladovo, 17.11.2023, N44 36.533 E22 38.028, 79 m,	Serbia	*Prunus cerasifera*		

		lgt. Toševski				
7065	*R. monzinii* (larva L3)	Serbia, Kostol, Kladovo, 17.11.2023, N44 36.533 E22 38.028, 79 m, lgt. Toševski	Serbia	*Prunus cerasifera*		
7066	*R. monzinii* (larva L3)	Serbia, Kostol, Kladovo, 17.11.2023, N44 36.533 E22 38.028, 79 m, lgt. Toševski	Serbia	*Prunus cerasifera*		
7068	*R. monzinii* (larva L3)	Serbia, Kostol, Kladovo, 17.11.2023, N44 36.533 E22 38.028, 79 m, lgt. Toševski	Serbia	*Prunus cerasifera*		
7070	*R. monzinii* (larva L3)	Serbia, Velika Vrbica, Kladovo, 17.11.2023 N44 36.072 E22 41.557, 59 m., lgt. Toševski	Serbia	*Prunus cerasifera*		
7073	*R. monzinii* (larva L3)	Serbia, Petrovo Selo, 17.11.2023 N44 37.732 E22 31.321, 217 m., lgt. Toševski	Serbia	*Prunus cerasifera*		
7076	*R. monzinii* (larva L3)	Serbia, Petrovo Selo, 17.11.2023 N44 37.732 E22 31.321, 217 m., lgt. Toševski	Serbia	*Prunus cerasifera*		
7077	*R. monzinii* (larva L3)	Serbia, Petrovo Selo, 17.11.2023 N44 37.732 E22 31.321, 217 m., lgt. Toševski	Serbia	*Prunus spinosa*		
7078	*R. monzinii* (larva L3)	Serbia, Petrovo Selo, 17.11.2023 N44 37.732 E22 31.321, 217 m., lgt. Toševski	Serbia	*Prunus spinosa*		
7082	*R. monzinii* (larva L3)	Serbia, Petrovo Selo, 17.11.2023 N44 37.732 E22 31.321, 217 m., lgt. Toševski	Serbia	*Prunus spinosa*		
4536	*R. monzinii*	Serbia, Mt. Zlatibor, 9.07.2017, N43 47.310 E19 43.721, 662 m, lgt. Toševski	Serbia	*Prunus cerasifera*	mon6_COI (PV910562)	**1**
4896	*R. monzinii*	Serbia, Vlasina, Božićki Kanal, N42 40.997 E22 21.888, 1289 m, 21.06.2018, lgt. Toševski	Serbia	*Malus pumila*	mon7_COI (PV910563)	**1**
4538	*R. monzinii*	Serbia, Mt. Zlatibor, 9.07.2017, N43 47.310 E19 43.721, 662 m, lgt. Toševski	Serbia	*Prunus cerasifera*	mon8_COI (PV910564)	**1**
4882	*R. monzinii*	Serbia, Brusnik, Negotin, N44 6.489 E22 24.115, 322	Serbia	*Prunus spinosa*	mon9_COI (PV910565)	**1**

		m, 21.05.2018, lgt. Toševski				
4881	*R. monzinii*	Serbia, Brusnik, Negotin, N44 6.489 E22 24.115, 322 m, 21.05.2018, lgt. Toševski	Serbia	*Prunus spinosa*	mon10_COI (PV910566)	**1**
4889	*R. monzinii*	Serbia, Brusnik, Negotin, N44 6.489 E22 24.115, 322 m, 21.05.2018, lgt. Toševski	Serbia	*Prunus spinosa*	mon11_COI (PV910567)	**1**
4859	*R. monzinii*	Serbia, Brusnik, Negotin, N44 6.489 E22 24.115, 322 m, 21.05.2018, lgt. Toševski	Serbia	*Cydonia oblonga*	mon12_COI (PV910568)	**1**
4857	*R. monzinii*	Serbia, Brusnik, Negotin, N44 6.489 E22 24.115, 322 m, 21.05.2018, lgt. Toševski	Serbia	*Cydonia oblonga*	mon13_COI (PV910569)	**1**
4893	*R. monzinii*	Serbia, Vlasina, Božićki Kanal, N42 40.997 E22 21.888, 1289 m, 21.06.2018, lgt. Toševski	Serbia	*Prunus cerasifera*	mon14_COI (PV910570)	**2**
5258	*R. monzinii*	Serbia, Brusnik, Negotin, N44 6.489 E22 24.115, 322 m, 21.05.2018, lgt. Toševski	Serbia	*Prunus spinosa*		
4906	*R. monzinii*	Greece, Mt. Taygetos 10.07.2018, N37 04.155 E22 15.882, lgt. Toševski	Greece	*Prunus spinosa*	mon15_COI (PV910571)	**1**
4535	*R. monzinii*	Serbia, Mt. Zlatibor, 9.07.2017, N43 47.310 E19 43.721, 662 m, lgt. Toševski	Serbia	*Prunus cerasifera*	mon16_COI (PV910572)	**1**
6586	*R. monzinii*	Greece, Arethousa, 23.06.2023, Macedonia, N40 44.299 E23 34.854, lgt. Toševski	Greece	*Prunus spinosa*	mon17_COI (PV910573)	**5**
6587	*R. monzinii*	Greece, Arethousa, 23.06.2023, Macedonia, N40 44.299 E23 34.854, lgt. Toševski	Greece	*Prunus spinosa*		
6588	*R. monzinii*	Greece, Arethousa, 23.06.2023, Macedonia, N40 44.299 E23 34.854, lgt. Toševski	Greece	*Prunus spinosa*		
6589	*R. monzinii*	Greece, Arethousa, 23.06.2023, Macedonia, N40 44.299 E23 34.854, lgt. Toševski	Greece	*Prunus spinosa*		
6592	*R. monzinii*	Greece, Arethousa, 23.06.2023, Macedonia,	Greece	*Prunus spinosa*		

		N40 44.299 E23 34.854, lgt. Toševski				
6585	*R. monzinii*	Greece, Arethousa, 23.06.2023, Macedonia, N40 44.299 E23 34.854, lgt. Toševski	Greece	*Prunus spinosa*	mon18_COI (PV910574)	**1**
6590	*R. monzinii*	Greece, Arethousa, 23.06.2023, Macedonia, N40 44.299 E23 34.854, lgt. Toševski	Greece	*Prunus spinosa*	mon19_COI (PV910575)	**1**
6591	*R. monzinii*	Greece, Arethousa, 23.06.2023, Macedonia, N40 44.299 E23 34.854, lgt. Toševski	Greece	*Prunus spinosa*	mon20_COI (PV910576)	**1**
7072	*R. monzinii* (larva L3)	Serbia, Velika Vrbica, Kladovo, 17.11.2023 N44 36.072 E22 41.557, 59 m., lgt. Toševski	Serbia	*Prunus spinosa*	mon21_COI (PV910577)	**1**
						**69**

[a] Diotti, L., Caldara, R., & Toševski, I. (2021). Description of two new species of *Rhamphus* related to *R. oxyacanthae* (Curculionidae, Curculioninae, Rhamphini) from Italy based on a morphological study supported by molecular data. *Zootaxa*, 4995(1), 111-128.

Rhamphus diottii **sp. n. Toševski & Caldara**

**Voucher DNA**	**Species**	**Location**	**Origin**	**Host**	**COI haplotype name (accession number)**	**Frequency**
4409	*R. diottii* sp.n PARATYPE	Serbia, Slankamen Vinogradi, 29.05.2009, N45 9.715 E20 11.750, 224 m., lgt. Toševski	Serbia	*Prunus spinosa*	dio1_COI (PV910578)	**10**
5262	*R. diottii* sp.n PARATYPE	Serbia, Slankamen Vinogradi, 29.05.2009, N45 9.715 E20 11.750, 224 m., lgt. Toševski	Serbia	*Prunus spinosa*		
5263	*R. diottii* sp.n PARATYPE	Serbia, Slankamen Vinogradi, 29.05.2009, N45 9.715 E20 11.750, 224 m., lgt. Toševski	Serbia	*Prunus spinosa*		
5264	*R. diottii* sp.n PARATYPE	Serbia, Slankamen Vinogradi, 29.05.2009, N45 9.715 E20 11.750, 224 m., lgt. Toševski	Serbia	*Prunus spinosa*		
5269	*R. diottii* sp.n PARATYPE	Serbia, Slankamen Vinogradi, 29.05.2009, N45 9.715 E20 11.750, 224 m., lgt. Toševski	Serbia	*Prunus spinosa*		
5270	*R. diottii* sp.n. **HOLOTYPE**	Serbia, Slankamen Vinogradi, 29.05.2009, N45	Serbia	*Prunus spinosa*		

		9.715 E20 11.750, 224 m., lgt. Toševski				
6344	*R. diottii* sp.n PARATYPE	Serbia, Slankamen Vinogradi, N45 9.610 E20 11.903, 15.06.2021, lgt. Toševski	Serbia	*Prunus spinosa*		
6345	*R. diottii* sp.n PARATYPE	Serbia, Sl. Vinogradi, N45 9.610 E20 11.903, 15.06.2021, lgt. Toševski	Serbia	*Prunus spinosa*		
6399	*R. diottii* sp.n PARATYPE	Serbia, Backa, Ada, 24.10.2022 N45 47.786 E20 05.647 (L3 larva), lgt. Toševski	Serbia	*Prunus cerasifera*		
6395	*R. diottii* sp.n PARATYPE	Serbia, Dobanovci, 15.06.2021, 4.11.2022, N44 51.034 E20 11.132, lgt. Toševski	Serbia	*Prunus cerasifera*		
5266	*R. diottii* sp.n PARATYPE	Serbia, Slankamen Vinogradi, 10.06.2017, N45 9.715 E20 11.750, 224 m., lgt. Toševski	Serbia	*Prunus spinosa*	dio2_COI (PV910579)	**6**
5268	*R. diottii* sp.n PARATYPE	Serbia, Slankamen Vinogradi, 10.06.2017, N45 9.715 E20 11.750, 224 m., lgt. Toševski	Serbia	*Prunus spinosa*		
6348	*R. diottii* sp.n PARATYPE	Serbia, Slankamen Vinogradi, 15.06.2021, N45 9.610 E20 11.903, lgt. Toševski	Serbia	*Prunus spinosa*		
6349	*R. diottii* sp.n PARATYPE	Serbia, Slankamen Vinogradi, 15.06.2021, N45 9.610 E20 11.903, lgt. Toševski	Serbia	*Prunus spinosa*		
6396	*R. diottii* sp.n (larva L3)	Serbia, Dobanovci, 4.11.2022, N44 51.034 E20 11.132, 76 m., lgt. Toševski	Serbia	*Prunus cerasifera*		
6415	*R. diottii* sp.n (larva L3)	Serbia, Slankamen Vinogradi, 29.09.2022, (L3 larva) N45 9.715 E20 11.750, 224 m, lgt. Toševski	Serbia	*Prunus spinosa*		
4410	*R. diottii* sp.n PARATYPE	Serbia, Slankamen Vinogradi, 29.05.2009, N45 9.715 E20 11.750, 224 m., lgt. Toševski	Serbia	*Prunus spinosa*	dio3_COI (PV910580)	**1**
4542	*R. diottii* sp.n PARATYPE	Serbia, Slankamen Vinogradi, 10.06.2017, N45 9.715 E20 11.750, 224 m., lgt. Toševski	Serbia	*Prunus spinosa*	dio4_COI (PV910581)	**1**
5265	*R. diottii* sp.n	Serbia, Slankamen Vinogradi, 10.06.2017, N45	Serbia	*Prunus spinosa*	dio5_COI	**3**

	PARATYPE	9.715 E20 11.750, 224 m., lgt. Toševski			(PV910582)	
6347	*R. diottii* sp.n PARATYPE	Serbia, Slankamen Vinogradi, N45 9.610 E20 11.903, 15.06.2021, lgt. Toševski	Serbia	*Prunus spinosa*		
6346	*R. diottii* sp.n PARATYPE	Serbia, Slankamen Vinogradi, 15.06.2021, N45 9.610 E20 11.903, lgt. Toševski	Serbia	*Prunus spinosa*		
5267	*R. diottii* sp.n PARATYPE	Serbia, Slankamen Vinogradi, 10.06.2017, N45 9.715 E20 11.750, 224 m., lgt. Toševski	Serbia	*Prunus spinosa*	dio6_COI (PV910583)	**1**
6401	*R. diottii* sp.n (larva L3)	Serbia, Backa, Ada, 24.10.2022 N45 47.786 E20 05.647 (L3 larva), lgt. Toševski	Serbia	*Prunus cerasifera*	dio7_COI (PV910584)	**1**
						**23**

Rhamphus ibericus **sp. n. Toševski & Caldara**

Voucher DNA	Species	Location	Origin	Host	COI haplotype name (accession number)	Frequency
6593	*R. ibericus* sp.n. PARATYPE	España, País Vasco, Araba/Álava, Elburgo-Burgelu, 556 m, N 42.812 W 2.633, 15.06.2023, lgt. Iñigo Ugarte San Vicente & Fernando Salgueira	Spain	*Prunus spinosa*	ibe1_COI (PV910585)	**3**
6594	*R. ibericus* sp.n. PARATYPE	España, País Vasco, Araba/Álava, Elburgo-Burgelu, 556 m, N 42.812 W 2.633, 15.06.2023, lgt. Iñigo Ugarte San Vicente & Fernando Salgueira	Spain	*Prunus spinosa*		
6598	*R. ibericus* sp.n. **HOLOTYPE**	España, País Vasco, Araba/Álava, Elburgo-Burgelu, 556 m, N 42.812 W 2.633, 15.06.2023, lgt. Iñigo Ugarte San Vicente & Fernando Salgueira	Spain	*Prunus spinosa*		
						**3**

Rhamphus subaeneus Illiger, 1808						
**Voucher DNA**	**Species**	**Location**	**Origin**	**Host**	**COI haplotype name (accession number)**	**Frequency**
5331	*R. subaeneus*	Spain, San Vicente de Arana, La Dehesa Álava, 8.07.2018, lgt. Iñigo Ugarte San Vicente & Fernando Salgueira	Spain	*Crataegus monogyna*	sub1_COI (PV910618)	**4**
5337	*R. subaeneus*	Spain, San Vicente de Arana, La Dehesa, Álava, 7.07.2018, lgt. Iñigo Ugarte San Vicente & Fernando Salgueira	Spain	*Crataegus monogyna*		
5338	*R. subaeneus*	Spain, San Vicente de Arana, La Dehesa, Álava, 7.07.2018, lgt. Iñigo Ugarte San Vicente & Fernando Salgueira	Spain	*Crataegus monogyna*		
6601	*R. subaeneus*	España, País Vasco, provincia de Araba/Álava, Subijana de Álava, 518 m s.n.m., 25-VI-2019, lgt. Iñigo Ugarte San Vicente & Fernando Salgueira	Spain	*Crataegus monogyna*		
5336	*R. subaeneus*	Spain, San Vicente de Arana, La Dehesa, Álava, 7.07.2018, lgt. Iñigo Ugarte San Vicente & Fernando Salgueira	Spain	*Crataegus monogyna*	sub2_COI (PV910619)	**1**
6094	*R. subaeneus*	Czechia, Bohemia Centr., Chramosty, Brdce hill, N49°40'12", E14°19'57", 415 m., 21.05.2020, lgt J. Kratky	Czechia	*Crataegus* sp.	sub3_COI (PV910586)	**1**
						**6**

Rhamphus cerdanicus Tempère, 1982						
**Voucher DNA**	**Species**	**Location**	**Origin**	**Host**	**COI haplotype name (accession number)**	**Frequency**
**6577**	*R. cerdanicus*	Spain, Pais Vasco, Araba/ Álava, Elburgo, 30TWN34, 550 m, 23.06.2007, lgt. Ugarte Salgueira & Fernando Salgueira	Spain	*Prunus spinosa*	cer1_COI (PV910587)	**3**

6596	*R. cerdanicus*	España, País Vasco, Araba/Álava, Elburgo-Burgelu, 556 m, N 42.812 W 2.633, 15.06.2023, lgt. Iñigo Ugarte San Vicente & Fernando Salgueira	Spain	*Prunus spinosa*		
6597	*R. cerdanicus*	España, País Vasco, Araba/Álava, Elburgo-Burgelu, 556 m, N 42.812 W 2.633, 15.06.2023, lgt. Iñigo Ugarte San Vicente & Fernando Salgueira	Spain	*Prunus spinosa*		
**6595**	*R. cerdanicus*	España, País Vasco, Araba/Álava, Elburgo-Burgelu, 556 m, N 42.812 W 2.633, 15.06.2023, lgt. Iñigo Ugarte San Vicente & Fernando Salgueira	Spain	*Prunus spinosa*	cer2_COI (PV910588)	**1**
						**4**

Rhamphus loebli Germann & Colonnelli, 2018

Voucher DNA	Species	Location	Origin	Host	COI haplotype name (accession number)	Frequency
6580	*R. loebli*	Spain, E. Castilia, Srr. Francia, La Alberca, env., 1075 m, 40°31'49"N, 06°08'44"W, 25.05.2019, lgt. J. Kratky	Spain	*Halimium sp.*	loe1_COI (PV910620)	**1**
7352	*R. loebli*	Spain, E. Andalucia, 4 km N of Rociana del Condado, 37.344 N 6.596 W, 10.03.2011, lgt. J. Kratky	Spain	*Halimium sp.*	loe2_COI (PX149871)	**1**
						**2**

Rhamphus hisamatsui Chûjô & Morimoto, 1960

Voucher DNA	Species	Location	Origin	Host	COI haplotype name (accession number)	Frequency
6328	*R. hisamatsui*	Japan, Inugoeji forest, Yamakita town, Kanagawa pref., 28.06.2021,	Japan	*Acer pictum*	his1_COI (PV910621)	**7**

		lgt. Y. Notsu		ssp. *mono*		
6329	*R. hisamatsui*	Japan, Inugoeji forest, Yamakita town, Kanagawa pref., 28.06.2021, lgt. Y. Notsu	Japan	*Acer pictum* ssp. *mono*		
6330	*R. hisamatsui*	Japan, Inugoeji forest, Yamakita town, Kanagawa pref., 28.06.2021, lgt. Y. Notsu	Japan	*Acer pictum* ssp. *mono*		
6331	*R. hisamatsui*	Japan, Inugoeji forest, Yamakita town, Kanagawa pref., 28.06.2021, lgt. Y. Notsu	Japan	*Acer pictum* ssp. *mono*		
6332	*R. hisamatsui*	Japan, Inugoeji forest, Yamakita town, Kanagawa pref., 28.06.2021, lgt. Y. Notsu	Japan	*Acer pictum* ssp. *mono*		
6333	*R. hisamatsui*	Japan, Inugoeji forest, Yamakita town, Kanagawa pref., 28.06.2021, lgt. Y. Notsu	Japan	*Acer pictum* ssp. *mono*		
6335	*R. hisamatsui*	Japan, Mikuni pass., Yamanakako vlg., Yamanashi pref., 12.06.2021, lgt. Y. Notsu	Japan	*Acer pictum* ssp. *mono*		
						**7**

**Total**	**326**
