# Supplementary material for: An Integrative Revision of the Genus Rhamphus (Curculionidae) from the Western Palearctic: Morphological and Molecular Data Reveal the Radiation of Multiple Species
Source: Insects. 2025 Nov 3;16(11):1123. doi: 10.3390/insects16111123 (PMC12653807; doi:10.3390/insects16111123)
Supplement: Supplementary file 1 [file insects-16-01123-s001.zip › Table_S2.pdf]

Table S2. List of *Rhamphus* specimens sequenced for *elongation factor-1α* (*EF-1α*) gene, sorted by DNA voucher code, species name, locality, country of origin, host plant affiliation, haplotype name with accession number in NCBI GenBank and frequency.

Rhamphus oxyacanthae **(Marsham, 1802)**						
**Voucher DNA**	**Species**	**Location**	**Origin**	**Host**	**Ef haplotype name (accession number)**	**Frequency**
4534	*R. oxyacanthae*	Serbia, Mt. Zlatibor, 9.07.2017, N43 47.310 E19 43.721, 662 m, lgt. Toševski	Serbia	*Crataegus* sp.	oxy1_Ef (PV943804)	**40**
4537	*R. oxyacanthae*	Serbia, Mt. Zlatibor, 9.07.2017, GPS 82: N43 47.310 E19 43.721, 662 m, lgt. Toševski	Serbia	*Crataegus* sp.		
4854	*R. oxyacanthae*	Serbia, Pitot, Staničenje, 20.05.2018, N43 13.020 E22 30.556, 403 m, lgt. Toševski	Serbia	*Crataegus* sp.		
4856	*R. oxyacanthae*	Serbia, Brusnik, Negotin, N44 6.489 E22 24.115, 322 m, 21.05.2018, lgt. Toševski	Serbia	*Cydonia oblonga*		
4891	*R. oxyacanthae*	Serbia, Vlasina, N42 40.573 E22 18.818, 1246 m, 21.06.2018, lgt. Toševski	Serbia	*Crataegus* sp.		
4892	*R. oxyacanthae*	Serbia, Vlasina, Bozicki Kanal, N42 40.997 E22 21.888, 1289 m, 21.06.2018, lgt. Toševski	Serbia	*Prunus cerasifera*		
4895	*R. oxyacanthae*	Serbia, Vlasina, Bozicki Kanal, N42 40.997 E22 21.888, 1289 m, 21.06.2018, lgt. Toševski	Serbia	*Prunus cerasifera*		
4907	*R. oxyacanthae*	Greece, Mt. Taygetos 10.07.2018, N37 04.155 E22 15.882, 1381 m, lgt. Toševski	Greece	*Pyrus spinosa*		
4908	*R. oxyacanthae*	Greece, Mt. Taygetos 10.07.2018, N37 04.155 E22 15.882, 1381 m, lgt. Toševski	Greece	*Pyrus spinosa*		
5160	*R. oxyacanthae*	Italy, Giaglione, (TO), Val Clarea, 7.07.2018, lgt. L. Diotti	Italy	-		
5161	*R. oxyacanthae*	Italy, Giaglione, (TO), Val Clarea, 7.07.2018, lgt. L. Diotti	Italy	-		
5168	*R. oxyacanthae*	Italy, Basilicata, bosco di Accettura, 10.06.2018, lgt.	Italy	-		

		L. Diotti				
5170	*R. oxyacanthae*	Italy, Basilicata, bosco di Accettura, 10.06.2018, lgt. L. Diotti	Italy	-		
5171	*R. oxyacanthae*	Italy, Basilicata, bosco di Accettura, 10.06.2018, lgt. L. Diotti	Italy	-		
5172	*R. oxyacanthae*	Italy, Muro Lucano (PT), Monte Paratiello, 10.06.2018, lgt. L. Diotti	Italy	-		
5173	*R. oxyacanthae*	Italy, Muro Lucano (PT), Monte Paratiello, 10.06.2018, lgt. L. Diotti	Italy	-		
5178	*R. oxyacanthae*	Italy, Emilia (Parma), Passo Cirrone 225 m, 8.08.2018 lgt. L. Diotti	Italy	-		
5182	*R. oxyacanthae*	Italy, Fino Mornasco, (CO), Ville dei Mulini, 25.05.2018, lgt. L. Diotti	Italy	-		
5271	*R. oxyacanthae*	Greece, Mt. Taygetos 10.07.2018, N37 04.155 E22 15.882, 1381 m, lgt. Toševski	Greece	*Pyrus spinosa*		
5273	*R. oxyacanthae*	Greece, Mt. Taygetos 10.07.2018, N37 04.155 E22 15.882, 1381 m, lgt. Toševski	Greece	*Pyrus spinosa*		
5274	*R. oxyacanthae*	Greece, Mt. Taygetos 10.07.2018, N37 04.155 E22 15.882, 1381 m, lgt. Toševski	Greece	*Pyrus spinosa*		
5275	*R. oxyacanthae*	Greece, Mt. Taygetos 10.07.2018, N37 04.155 E22 15.882, 1381 m, lgt. Toševski	Greece	*Pyrus spinosa*		
5332	*R. oxyacanthae*	Spain, San Vicente sa de logiz, Álava, 8.07.2018, lgt. Iñigo Ugarte San Vicente & Fernando Salgueira	Spain	*Crataegus monogyna*		
5335	*R. oxyacanthae*	Spain, San Vicente sa de logiz, Álava, 8.07.2018, lgt. Iñigo Ugarte San Vicente & Fernando Salgueira	Spain	*Crataegus monogyna*		
5344	*R. oxyacanthae*	Greece, Mt. Taygetos 10.07.2018, N37 04.155 E22 15.882, 1381 m, lgt. Toševski	Greece	*Pyrus spinosa*		
5345	*R. oxyacanthae*	Greece, Mt. Taygetos 10.07.2018, N37 04.155 E22 15.882, 1381 m, lgt.	Greece	*Pyrus spinosa*		

		Toševski				
5346	*R. oxyacanthae*	Greece, Mt. Taygetos 10.07.2018, N37 04.155 E22 15.882, 1381 m, lgt. Toševski	Greece	*Pyrus spinosa*		
5953	*R. oxyacanthae* **NEOTYPE**	England, Suffolk, TL7086, 23.06.2020., lgt. H. Mandel	England	*Crataegus* sp.		
5954	*R. oxyacanthae*	England, Suffolk, TL7086, 23.06.2020., lgt. H. Mandel	England	*Crataegus* sp.		
5955	*R. oxyacanthae*	England, Suffolk, TL7086, 23.06.2020., lgt. H. Mandel	England	*Crataegus* sp.		
5956	*R. oxyacanthae*	England, Suffolk, TL7086, 23.06.2020., lgt. H. Mandel	England	*Crataegus* sp.		
5957	*R. oxyacanthae*	England, Suffolk, TL7086, 23.06.2020., lgt. H. Mandel	England	*Crataegus* sp.		
5959	*R. oxyacanthae*	England, Suffolk, TL7086, 23.06.2020., lgt. H. Mandel	England	*Crataegus* sp.		
6012	*R. oxyacanthae*	F. 33, Queyrac, Le Gross Cap, 4.05.2018, lgt. Lessieur David	France	Crataegus monogyna		
6013	*R. oxyacanthae*	F. 33, Queyrac, Le Gross Cap, 4.05.2018, lgt. Lessieur David	France	Crataegus monogyna		
6014	*R. oxyacanthae*	F. 33, Queyrac, Le Gross Cap, 4.05.2018, lgt. Lessieur David	France	Crataegus monogyna		
6015	*R. oxyacanthae*	F. 33, Queyrac, Le Gross Cap, 4.05.2018, lgt. Lessieur David	France	Crataegus monogyna		
6324	*R. oxyacanthae*	Italy, Sila Grande, (CS) Lago di Cecita, 17.06.2015, lgt. L. Diotti	Italy	-		
6325	*R. oxyacanthae*	Italy, Sila Grande, (CS) Lago di Cecita, 17.06.2015, lgt. L. Diotti	Italy	-		
7382	*R. oxyacanthae*	Greece, Macedonia, Arethousa, 13.06.2024, N40 44.134 E23 36.193, 374 m. lgt. Toševski	Greece	*Crataegus* sp.		
5958	*R. oxyacanthae*	England, Suffolk, TL7086, 23.06.2020., lgt. H. Mandel	England	*Crataegus* sp.	oxy2_Ef (PV943771)	**1**
5177	*R. oxyacanthae*	Italy, Emilia (Parma), Passo Cirrone 225 m, 8.08.2018 lgt. L. Diotti	Italy	-	oxy3_Ef (PV943772)	**2**
5181	*R. oxyacanthae*	Italy, Fino Mornasco, (CO), Ville dei Mulini, 25.05.2018, lgt. L. Diotti	Italy	-		
5159	*R. oxyacanthae*	Italy, Giaglione, (TO), Val Clarea, 7.07.2018, lgt. L.	Italy	-	oxy4_Ef (PV943773)	**1**

		Diotti				
5272	*R. oxyacanthae*	Greece, Mt. Taygetos 10.07.2018, N37 04.155 E22 15.882, 1381 m, lgt. Toševski	Greece	*Pyrus spinosa*	oxy5_Ef (PV943805)	**1**
5276	*R. oxyacanthae*	Greece, Mt. Taygetos 10.07.2018, N37 04.155 E22 15.882, 1381 m, lgt. Toševski	Greece	*Pyrus spinosa*	oxy6_Ef (PV943806)	**1**
4860	*R. oxyacanthae*	Serbia, Brusnik, Negotin, N44 6.489 E22 24.115, 322 m, 21.05.2018, lgt. Toševski	Serbia	*Pyrus* sp.	oxy7_Ef (PV943807)	**1**
6602	*R. oxyacanthae*	Spain, País Vasco, prov. de Araba/ Álava, Subijana de Álava, 518 m s.n.m., 25.06.2019, lgt. Iñigo Ugarte San Vicente & Fernando Salgueira		*Crataegus monogyna*	oxy8_Ef (PV943774)	**1**
						**48**

Rhamphus bavierai **Diotti, Caldara & Toševski, 2021**						
**Voucher DNA**	**Species**	**Location**	**Origin**	**Host**	**Ef haplotype name (accession number)**	**Frequency**
4403	*R. bavierai*	Sicily, Piano Battaglia, 1600 m., 15.07.2016, lgt. Baviera C.	Sicily	*Crataegus monogyna, C. laciniata*	bav1_Ef (PV943775)	**17**
4404	*R. bavierai*	Sicily, Piano Battaglia, 1600 m., 15.07.2016, lgt Baviera C.	Sicily	*Crataegus monogyna, C. laciniata*		
4405	*R. bavierai*	Sicily, Piano Battaglia, 1600 m, 15.07.2016, lgt Baviera C.	Sicily	*Crataegus monogyna, C. laciniata*		
4406	*R. bavierai*	Sicily, Piano Battaglia, 1600 m, 15.07.2016, lgt Baviera C.	Sicily	*Crataegus monogyna, C. laciniata*		
4407	*R. bavierai*	Sicily, Piano Battaglia, 1600 m., 15.07.2016, lgt Baviera C.	Sicily	*Crataegus monogyna, C. laciniata*		
5979	*R. bavierai*	Sicily, Messina: Nebrodi Caronia, 1400 m., 20.06.2020, lgt. Baviera C.	Sicily	*Crataegus monogyna, C. laciniata*		
5977	*R. bavierai*	Sicily, Messina: Nebrodi Caronia, 1400 m., 20.06.2020, lgt. Baviera C.	Sicily	*Crataegus monogyna,*		

				C. laciniata		
5980	*R. bavierai*	Sicily, Messina: Nebrodi Caronia, 1400 m., 20.06.2020, lgt. Baviera C.	Sicily	*Crataegus monogyna, C. laciniata*		
5981	*R. bavierai*	Sicily, Messina: Nebrodi Caronia, 1400 m., 20.06.2020, lgt. Baviera C.	Sicily	*Crataegus monogyna, C. laciniata*		
5982	*R. bavierai*	Sicily, Messina: Nebrodi Caronia, 1400 m., 20.06.2020, lgt. Baviera	Sicily	*Crataegus monogyna, C. laciniata*		
5983	*R. bavierai*	Sicily, Messina: Nebrodi Caronia, 1400 m., 20.06.2020, lgt. Baviera C.	Sicily	*Crataegus monogyna, C. laciniata*		
5984	*R. bavierai*	Sicily, Messina: Nebrodi Caronia, 1400 m., 20.06.2020, lgt. Baviera C.	Sicily	*Crataegus monogyna, C. laciniata*		
5985	*R. bavierai*	Sicily, Messina: Nebrodi Caronia, 1400 m., 20.06.2020 lgt. Baviera C.	Sicily	*Crataegus monogyna, C. laciniata*		
5986	*R. bavierai*	Sicily, Messina: Nebrodi Caronia, 1400 m., 20.06.2020, lgt. Baviera C.	Sicily	*Crataegus monogyna, C. laciniata*		
6004	*R. bavierai*	Sicily, Piano Battaglia, 1600 m, 15.07.2016, lgt Baviera C.	Sicily	*Crataegus monogyna, C. laciniata*		
6005	*R. bavierai*	Sicily, Piano Battaglia, 1600 m, 15.07.2016, lgt Baviera C.	Sicily	*Crataegus monogyna, C. laciniata*		
5989	*R. bavierai*	Sicily, Messina: Nebrodi, Capizzi, 1450 m, Portella Obolo, 20.06.2020, lgt. Baviera C.	Sicily	*Crataegus monogyna, C. laciniata*		
5960	*R. bavierai*	Sicily, Palermo: Madonie, P. Zucchi, 1100 m a.s.l., 16.06.2020, lgt. Baviera C	Sicily	*Crataegus monogyna, C. laciniata*	bav2_Ef (PV943776)	**4**
5961	*R. bavierai*	Sicily, Palermo: Madonie, P. Zucchi, 1100 m a.s.l., 16.06.2020, lgt. Baviera C	Sicily	*Crataegus monogyna, C. laciniata*		
5965	*R. bavierai*	Sicily, Palermo: Madonie, Piano Zucchi, 1100 m a.s.l., 16.06.2020, lgt. Baviera C.	Sicily	*Crataegus monogyna, C. laciniata*		
5970	*R. bavierai*	Sicily, Catania: Etna 1200 m., Linguaglossa, 27.06.2020, lgt. Baviera C.	Sicily	*Crataegus monogyna, C. laciniata*		

5978	*R. bavierai*	Sicily, Messina: Nebrodi Caronia, 1400 m., 20.06.2020, lgt. Baviera C.	Sicily	*Crataegus monogyna, C. laciniata*	bav3_Ef (PV943777)	**3**
5987	*R. bavierai*	Sicily, Messina: Nebrodi, Capizzi, 1450 m, Portella Obolo, 20.06.2020 lgt. Baviera C.	Sicily	*Crataegus monogyna, C. laciniata*		
5988	*R. bavierai*	Sicily, Messina: Nebrodi, Capizzi, 1450 m, Portella Obolo, 20.06.2020, lgt. Baviera C.	Sicily	*Crataegus monogyna, C. laciniata*		
5966	*R. bavierai*	Sicily, Catania: Etna 1200 m., Linguaglossa, 27.06.2020, lgt. Baviera C.	Sicily	*Crataegus monogyna, C. laciniata*	bav4_Ef (PV943778)	**1**
						**25**

Rhamphus hampsicora Diotti, Caldara & Toševski, 2021

Voucher DNA	Species	Location	Origin	Host	Ef haplotype name (accession number)	Frequency
5339	*R. hampsicora*	Sardinia, Siniscola (NU), Pendici Monte Albo, 25.05.2012, lgt. Diotti	Sardinia	*Prunus* sp.	ham1_Ef (PV943779)	**3**
5340	*R. hampsicora*	Sardinia, Siniscola (NU), Pendici Monte Albo, 25.05.2012, lgt. Diotti	Sardinia	*Prunus* sp.		
5341	*R. hampsicora*	Sardinia, Siniscola (NU), Pendici Monte Albo, 25.05.2012, lgt. Diotti	Sardinia	*Prunus* sp.		
5342	*R. hampsicora*	Sardinia, Siniscola (NU), Pendici Monte Albo, 25.05.2012, lgt. Diotti	Sardinia	*Prunus* sp.	ham2_Ef (PV943780)	**1**
						**4**

Rhamphus cypricus sp. n. Toševski & Caldara

Voucher DNA	Species	Location	Origin	Host	Ef haplotype name (accession number)	Frequency
6178	*R. cypricus* sp.n. PARATYPE	Cyprus, Paphos p., Drouseia env., 541 m. 23.03.2019, lgt. F. Pavel	Cyprus	no data	-	-
7346	*R. cypricus* sp.n. PARATYPE	Cyprus, Droushia-Ineia(Paphos, Chypre) ± 600m, 12.04.2005, G. et H. Alziar lgt. sur *Crataegus* fleuri. Collection G. Alziar	Cyprus	*Crataegus* sp.	-	-

7349	*R. cypricus* sp.n. PARATYPE	Cyprus, Droushia-Ineia(Paphos, Chypre) 600m, 12.04.2005, G. et H. Alziar lgt. sur Crataegus fleuri. Collection G. Alziar	Cyprus	*Crataegus* sp.	-	-
7372	*R. cypricus* sp.n. **HOLOTYPE**	Cyprus, Parsata, 7.04.2024, 34.8281745 N, 33.2648008 E, lgt. L. Forbicioni	Cyprus	*Crataegus azarolus*	cyp1_Ef (PV943808)	**8**
7373	*R. cypricus* sp.n. PARATYPE	Cyprus, Parsata, 7.04.2024, 34.8281745 N, 33.2648008 E, lgt. L. Forbicioni	Cyprus	*Crataegus azarolus*		
7374	*R. cypricus* sp.n. PARATYPE	Cyprus, Parsata, 7.04.2024, 34.8281745 N, 33.2648008 E, lgt. L. Forbicioni	Cyprus	*Crataegus azarolus*		
7375	*R. cypricus* sp.n. PARATYPE	Cyprus, Parsata, 7.04.2024, 34.8281745 N, 33.2648008 E, lgt. L. Forbicioni	Cyprus	*Crataegus azarolus*		
7376	*R. cypricus* sp.n. PARATYPE	Cyprus, Parsata, 7.04.2024, 34.8281745 N, 33.2648008 E, lgt. L. Forbicioni	Cyprus	*Crataegus azarolus*		
7377	*R. cypricus* sp.n. PARATYPE	Cyprus, Parsata, 7.04.2024, 34.8281745 N, 33.2648008 E, lgt. L. Forbicioni	Cyprus	*Crataegus azarolus*		
7379	*R. cypricus* sp.n. PARATYPE	Cyprus, Lefkara, 12.04.2024, 34.8692177 N, 33.2839350 E, lgt. L. Forbicioni	Cyprus	*Crataegus azarolus*		
7380	*R. cypricus* sp.n. PARATYPE	Cyprus, Lefkara, 12.04.2024, 34.8692177 N, 33.2839350 E, lgt. L. Forbicioni	Cyprus	*Crataegus azarolus*		
7371	*R. cypricus* sp.n. PARATYPE	Cyprus, Parsata, 7.04.2024, 34.8281745 N, 33.2648008 E, lgt. L. Forbicioni	Cyprus	*Crataegus azarolus*	cyp2_Ef (PV943809)	**2**
7378	*R. cypricus* sp.n. PARATYPE	Cyprus, Parsata, 7.04.2024, 34.8281745 N, 33.2648008 E, lgt. L. Forbicioni	Cyprus	*Crataegus azarolus*		
						**10**

Rhamphus macedonicus **sp. n. Toševski & Caldara**

Voucher DNA	Species	Location	Origin	Host	Ef haplotype name (accession number)	Frequency
6582	*R. macedonicus* sp.n. **HOLOTYPE**	Greece, Macedonia, Arethousa, 23.06.2023, N40 44.134 E23 36.193, 374 m. lgt. Toševski	Greece	*Crataegus* sp.	mac01_Ef (PV943810)	**15**
6583	*R. macedonicus* sp.n. PARATYPE	Greece, Macedonia, Arethousa, 23.06.2023, N40 44.134 E23 36.193, 374 m. lgt. Toševski	Greece	*Crataegus* sp.		
6584	*R. macedonicus* sp.n. PARATYPE	Greece, Macedonia, Arethousa, 23.06.2023, N40 44.134 E23 36.193,	Greece	*Crataegus* sp.		

		374 m. lgt. Toševski				
7381	*R. macedonicus* sp.n. PARATYPE	Greece, Macedonia, Arethousa, 13.06.2024, N40 44.134 E23 36.193, 374 m. lgt. J. Jović	Greece	*Crataegus* sp.		
7383	*R. macedonicus* sp.n. PARATYPE	Greece, Macedonia, Arethousa, 13.06.2024, N40 44.134 E23 36.193, 374 m. lgt. J. Jović	Greece	*Crataegus* sp.		
7384	*R. macedonicus* sp.n. PARATYPE	Greece, Macedonia, Arethousa, 13.06.2024, N40 44.134 E23 36.193, 374 m. lgt. J. Jović	Greece	*Crataegus* sp.		
7385	*R. macedonicus* sp.n. PARATYPE	Greece, Macedonia, Arethousa, 13.06.2024, N40 44.134 E23 36.193, 374 m. lgt. J. Jović	Greece	*Crataegus* sp.		
7386	*R. macedonicus* sp.n. PARATYPE	Greece, Macedonia, Arethousa, 13.06.2024, N40 44.134 E23 36.193, 374 m. lgt. J. Jović	Greece	*Crataegus* sp.		
7387	*R. macedonicus* sp.n. PARATYPE	Greece, Macedonia, Arethousa, 13.06.2024, N40 44.134 E23 36.193, 374 m. lgt. J. Jović	Greece	*Crataegus* sp.		
7388	*R. macedonicus* sp.n. PARATYPE	Greece, Macedonia, Arethousa, 13.06.2024, N40 44.134 E23 36.193, 374 m. lgt. J. Jović	Greece	*Crataegus* sp.		
7389	*R. macedonicus* sp.n. PARATYPE	Greece, Macedonia, Arethousa, 13.06.2024, N40 44.134 E23 36.193, 374 m. lgt. J. Jović	Greece	*Crataegus* sp.		
7390	*R. macedonicus* sp.n. PARATYPE	Greece, Macedonia, Arethousa, 13.06.2024, N40 44.134 E23 36.193, 374 m. lgt. J. Jović	Greece	*Crataegus* sp.		
7391	*R. macedonicus* sp.n. PARATYPE	Greece, Macedonia, Arethousa, 13.06.2024, N40 44.134 E23 36.193, 374 m. lgt. J. Jović	Greece	*Crataegus* sp.		
7392	*R. macedonicus* sp.n. PARATYPE	Greece, Macedonia, Arethousa, 13.06.2024, N40 44.134 E23 36.193, 374 m. lgt. J. Jović	Greece	*Crataegus* sp.		
7393	*R. macedonicus* sp.n. PARATYPE	Greece, Macedonia, Arethousa, 13.06.2024, N40 44.134 E23 36.193, 374 m. lgt. J. Jović	Greece	*Crataegus* sp.		
						**15**

Rhamphus pulicarius (Marsham, 1802)						
**Voucher DNA**	**Species**	**Location**	**Origin**	**Host**	**Ef haplotype name (accession number)**	**Frequency**
4541	*R. pulicarius*	Serbia, Draglica, Mt. Zlatar, N43 35.173 E19 43.645, 929 m., 12.07.2017, lgt. Toševski	Serbia	*Salix caprea*	pul1_Ef (PV943811)	**7**
4583	*R. pulicarius*	Serbia, Babin Zub, Stara Planina, N43 23.165 E22 35.549, 1250 m, 5.07.2017, lgt. Toševski	Serbia	*Salix caprea*		
4585	*R. pulicarius*	Serbia, Babin Zub, Stara Planina, N43 23.165 E22 35.549, 1250 m, 5.07.2017, lgt. Toševski	Serbia	*Salix caprea*		
5179	*R. pulicarius*	Italy, Lombardia (CO), dintorni Monguzzo, 7.06.2017, lgt. L. Diotti	Italy	-		
6223	*R. pulicarius*	Poland, Rudnik ad Lublin, 51°16'58.8"N 22°38'28.8"E, 15.06.2021, lgt. Rafał Gosik	Poland	*Betula* sp.		
7444	*R. pulicaries*	Greece, Prodromis, mined leave of *Salix* sp., GPS 006 N40 27.650 E23 23.248, 28.09.2024, lgt. Toševski	Greece	*Salix* sp.		
7446	*R. pulicaries*	Greece, Prodromis, mined leave of *Salix* sp., GPS 006 N40 27.650 E23 23.248, 28.09.2024, lgt. Toševski	Greece	*Salix* sp.		
4540	*R. pulicarius*	Serbia, Draglica, Mt. Zlatar, N43 35.173 E19 43.645, 929 m., 12.07.2017, lgt. Toševski	Serbia	*Salix caprea*	pul2_Ef (PV943812)	**3**
4580	*R. pulicarius*	Serbia, Draglica, Mt. Zlatar, N43 35.173 E19 43.645, 929 m., 12.07.2017, lgt. Toševski	Serbia	*Salix caprea*		
6353	*R. pulicarius*	England, 21/023, Lynford, Mundford, West Norfolk (VC28), TL8294, 16.06.2021, lgt. H. Mendel	England	-		
6227	*R. pulicarius*	Serbia, Jokino Vrelo, Kremna, Mt. Tara 24.06.2021, lgt. Toševski	Serbia	*Salix alba*	pul3_Ef (PV943813)	**1**
6010	*R. pulicarius*	France, F 19, Chavanac, 6.07.2020, 8.07.2020, lgt. Lessieur David	France	*Salix sp.*	pul4_Ef (PV943781)	**1**
5180	*R. pulicarius*	Italy, Lombardia (CO), dintorni Monguzzo, 7.06.2017, lgt. L. Diotti	Italy	-	pu5_Ef (PV943782)	**1**
6011	*R. pulicarius*	France, F 19, Chavanac, 6.07.2020, 8.07.2020, on Salix, lgt. Lessieur David	France	*Salix Isp.*	pul6_Ef (PV943783)	**1**
6226	*R. pulicarius*	Poland, Rudnik ad Lublin, 51°16'58.8"N 22°38'28.8"E, 15.06.2021, lgt. Rafał Gosik	Poland	*Salix* sp.	pul7_Ef (PV943814)	**1**

7442	*R. pulicaries*	Greece, Prodromis, mined leave of *Salix* sp., GPS 006 N40 27.650 E23 23.248, 28.09.2024, lgt. Toševski	Greece	*Salix* sp.	pul8_Ef (PV943815)	**1**
7443	*R. pulicaries*	Greece, Prodromis, mined leave of *Salix* sp., GPS 006 N40 27.650 E23 23.248, 28.09.2024, lgt. Toševski	Greece	*Salix* sp.	pul9_Ef (PV943816)	**3**
7445	*R. pulicaries*	Greece, Prodromis, mined leave of *Salix* sp., GPS 006 N40 27.650 E23 23.248, 28.09.2024, lgt. Toševski	Greece	*Salix* sp.		
7447	*R. pulicaries*	Greece, Prodromis, mined leave of *Salix* sp., GPS 006 N40 27.650 E23 23.248, 28.09.2024, lgt. Toševski	Greece	*Salix* sp.		
						**19**

Rhamphus pullus **Hustache, 1920**

**Voucher DNA**	**Species**	**Location**	**Origin**	**Host**	**Ef haplotype name (accession number)**	**Frequency**
6334	*R. pullus*	Japan, Mikuni pass., Yamanakako vlg., Yamanashi pref., 12.06.2021, lgt. Y. Notsu	Japan	*Betula* sp.	pull1_Ef (PV943784)	**2**
6336	*R. pullus*	Japan, Mikuni pass., Yamanakako vlg., Yamanashi pref., 12.06.2021, lgt. Y. Notsu	Japan	*Betula* sp.		
6337	*R. pullus*	Japan, Mikuni pass., Yamanakako vlg., Yamanashi pref., 12.06.2021, lgt. Y. Notsu	Japan	*Betula* sp.	pull2_Ef (PV943785)	**1**
						**3**

Rhamphus betulae **sp. n. Toševski & Caldara**

**Voucher DNA**	**Species**	**Location**	**Origin**	**Host**	**Ef haplotype name (accession number)**	**Frequency**
6608	*R. betulae* sp.n. PARATYPE	France, 65 Trébons, 43.0799872049 /0.113917399, 30.06.2023 bouhaben, lgt. D. Lessieur	France	*Betula* sp.	bet1_Ef (PV943817)	**2**
6610	*R. betulae* sp.n. PARATYPE	France, 65 Trébons, 43.0799872049 /0.113917399, 30.06.2023 bouhaben, lgt. D. Lessieur	France	*Betula* sp.		
6609	*R. betulae* sp.n. PARATYPE	France, 65 Trébons, 43.0799872049 /0.113917399, 30.06.2023	France	*Betula* sp.	bet2_Ef (PV943818)	**2**

		bouhaben, lgt. D. Lessieur					
6612	*R. betulae* sp.n. PARATYPE	France, 65 Trébons, 43.0799872049 /0.113917399, 30.06.2023 bouhaben, lgt. D. Lessieur	France	*Betula* sp.			
6317	*R. betulae* sp.n. PARATYPE	Italy, Val Sesia, (VC) Monte Tovo, 1100 m, 12.06,2021, lgt. L. Diotti	Italy	*Betula pendula*	bet3_Ef (PV943786)	**1**	
6315	*R. betulae* sp.n. PARATYPE	Italy, Val Sesia, (VC) Monte Tovo, 1100 m, 12.06,2021, lgt. L. Diotti	Italy	*Betula pendula*	bet4_Ef (PV943787)	**2**	
6316	R. betulae sp.n. **HOLOTYPE**	Italy, Val Sesia, (VC) Monte Tovo, 1100 m, 12.06,2021, lgt. L. Diotti	Italy	*Betula pendula*			
6319	*R. betulae* sp.n. PARATYPE	Italy, Val Sesia, (VC) Monte Tovo, 1100 m, 12.06,2021, lgt. L. Diotti	Italy	*Betula pendula*	bet5_Ef (PV943788)	**3**	
6221	*R. betulae* sp.n. PARATYPE	Poland, Rudnik ad Lublin 51°16'58.8"N 22°38'28.8"E, 15.06.2021, lgt. Rafal Gosik	Poland	*Betula* sp.			
6321	*R. betulae* sp.n. PARATYPE	Italy, Val Sesia, (VC) Monte Tovo, 1100 m, 12.06,2021, lgt. L. Diotti	Italy	*Betula pendula*			
6220	*R. betulae* sp.n. PARATYPE	Poland, Rudnik ad Lublin 51°16'58.8"N 22°38'28.8"E, 15.06.2021, lgt. Rafal Gosik	Poland	*Betula* sp.	bet6_Ef (PV943819)	**1**	
6217	*R. betulae* sp.n. PARATYPE	Poland, Rudnik ad Lublin 51°16'58.8"N 22°38'28.8"E, 15.06.2021, lgt. Rafal Gosik	Poland	*Betula* sp.	bet7_Ef (PV943820)	**1**	
6318	*R. betulae* sp.n. PARATYPE	Italy, Val Sesia, (VC) Monte Tovo, 1100 m, 12.06,2021, lgt. L. Diotti	Italy	*Betula pendula*	bet8_Ef (PV943789)	**1**	
6322	*R. betulae* sp.n. PARATYPE	Italy, Val Sesia, (VC) Monte Tovo, 1100 m, 12.06,2021, lgt. L. Diotti	Italy	*Betula pendula*	bet9_Ef (PV943790)	**1**	
6320	*R. betulae* sp.n. PARATYPE	Italy, Val Sesia, (VC) Monte Tovo, 1100 m, 12.06,2021, lgt. L. Diotti	Italy	*Betula pendula*	bet10_Ef (PV943791)	**1**	
6224	*R. betulae* sp.n. PARATYPE	Poland, Rudnik ad Lublin 51°16'58.8"N 22°38'28.8"E, 15.06.2021, lgt. Rafal Gosik	Poland	*Betula* sp.	bet11_Ef (PV943821)	**1**	
6219	*R. betulae* sp.n. PARATYPE	Poland, Rudnik ad Lublin 51°16'58.8"N 22°38'28.8"E, 15.06.2021, lgt. Rafal Gosik	Poland	*Betula* sp.	bet12_Ef (PV943822)	**1**	
6225	*R. betulae* sp.n. PARATYPE	Poland, Rudnik ad Lublin 51°16'58.8"N 22°38'28.8"E, 15.06.2021, lgt. Rafal Gosik	Poland	*Betula* sp.	bet13_Ef (PV943823)	**1**	
6607	*R. betulae* sp.n. PARATYPE	France, 65 Trébons, 43.0799872049 /0.113917399, 30.06.2023 bouhaben, lgt. D. Lessieur	France	*Betula* sp.	bet14_Ef (PV943824)	**2**	
6613	*R. betulae* sp.n. PARATYPE	France - 65 Pouzac, 43.0758181239 /0.116744447, 2.07.2023, gardeloup, lgt. D. Lessieur	France	*Betula* sp.			

						**20**

***Rhamphus crypticus* sp. n. Toševski & Caldara**						
**Voucher DNA**	**Species**	**Location**	**Origin**	**Host**	**Ef haplotype name (accession number)**	**Frequency**
6352	*R. crypticus* sp.n. **HOLOTYPE**	England, 21/023, Lynford, Mundford, West Norfolk (VC28), TL8294, lgt. H. Mendel	England	*Salix* sp.	cry1_Ef (PV943792)	**9**
6006	*R. crypticus* sp.n. PARATYPE	England, New Forest, S. Hants, SU2404, 13.07.2020, lgt. H. Mendel	England	*Salix* sp.		
6007	*R. crypticus* sp.n. PARATYPE	England, New Forest, S. Hants, SU2404, 13.07.2020, lgt. H. Mendel	England	*Salix* sp.		
6008	*R. crypticus* sp.n. PARATYPE	England, New Forest, S. Hants, SU2404, 13.07.2020, lgt. H. Mendel	England	*Salix* sp.		
6009	*R. crypticus* sp.n. PARATYPE	England, New Forest, S. Hants, SU2404, 13.07.2020, lgt. H. Mendel	England	*Salix* sp.		
6354	*R. crypticus* sp.n. PARATYPE	England, 21/026, Denge Beach, East Kent (VC15), TLR0817 28.06.2021, lgt. H. Mendel	England	*Salix* sp.		
6355	*R. crypticus* sp.n. PARATYPE	England, 21/026, Denge Beach, East Kent (VC15), TLR0817 28.06.2021, lgt. H. Mendel	England	*Salix* sp.		
6356	*R. crypticus* sp.n. PARATYPE	England, 21/026, Denge Beach, East Kent (VC15), TLR0817, 28.06.2021, lgt. H. Mendel	England	*Salix* sp.		
6618	*R. crypticus* sp.n. PARATYPE	France, Hautes-Pyrénées (65) - Poueyferré, 43.112498397 /-0.093337389, 29.06.2023, Tourbiére de Lourdes, lgt. D. Lessieur	France	*Salix atrocinerea*		
6018	*R. crypticus* sp.n. PARATYPE	France, F 19, La Tronche (19110), la croix de Layre, 16.VII.2020, lgt. D. Lessieur	France	-	cry2_Ef (PV943793)	**9**
6358	*R. crypticus* sp.n. PARATYPE	England, 21/026, Denge Beach, East Kent (VC15), TLR0817, 28.06.2021, lgt. H. Mendel	England	*Salix* sp.		

6359	*R. crypticus* sp.n. PARATYPE	England, 21/026, Denge Beach, East Kent (VC15), TLR0817, 28.06.2021, lgt. H. Mendel	England	*Salix* sp.		
6615	*R. crypticus* sp.n. PARATYPE	France, Hautes-Pyrénées (65) - Poueyferré, 43.112498397 /-0.093337389, 29.06.2023, Tourbiére de Lourdes, lgt. D. Lessieur	France	*Salix atrocinerea*		
6616	*R. crypticus* sp.n. PARATYPE	France, Hautes-Pyrénées (65) - Poueyferré, 43.112498397 /-0.093337389, 29.06.2023, Tourbiére de Lourdes, lgt. D. Lessieur	France	*Salix atrocinerea*		
6617	*R. crypticus* sp.n. PARATYPE	France, Hautes-Pyrénées (65) - Poueyferré, 43.112498397 /-0.093337389, 29.06.2023, Tourbiére de Lourdes, lgt. D. Lessieur	France	*Salix atrocinerea*		
6622	*R. crypticus* sp.n. PARATYPE	France - 65 Pouzac, 43.076836910 /0.119550038, 28.06.2023, gardeloup, lgt. D. Lessieur	France	*Salix atrocinerea*		
6623	*R. crypticus* sp.n. PARATYPE	France - Hautes-Pyrénées (65) Banios, 43.0409039 /0.233232, 1.07.2023, lgt. D. Lessieur	France	*Salix atrocinerea*		
6624	*R. crypticus* sp.n. PARATYPE	France, Hautes-Pyrénées (65) Banios, 43.0409039 /0.233232, 1.07.2023, lgt. D. Lessieur	France	*Salix atrocinerea*		
6357	*R. crypticus* sp.n. PARATYPE	England, 21/026, Denge Beach, East Kent (VC15), TLR0817, 28.06.2021, lgt. H. Mendel	England	*Salix* sp.	cry3_Ef (PV943794)	**2**
6360	*R. crypticus* sp.n. PARATYPE	England, 21/026, Denge Beach, East Kent (VC15), TLR0817, 28.06.2021, lgt. H. Mendel	England	*Salix* sp.		
6599	*R. crypticus* sp.n. PARATYPE	Spain, provincia de Soria, Vinuesa, río Revinuesa, 13-VII-2020, lgt. Iñigo Ugarte San Vicente & Fernando Salgueira	Spain	*Salix salviifolia*	cry4_Ef (PV943795)	**1**
6600	*R. crypticus* sp.n. PARATYPE	Spain, provincia de Soria, Vinuesa, río Revinuesa, 13-VII-2020, lgt. Iñigo Ugarte	Spain	*Salix salviifolia*	cry5_Ef (PV943796)	**1**

		San Vicente & Fernando Salgueira				
						**22**

***Rhamphus monzinii* Pesarini & Diotti, 2012**						
**Voucher DNA**	**Species**	**Location**	**Origin**	**Host**	**Ef haplotype name (accession number)**	**Frequency**
4535	*R. monzinii*	Serbia, Mt. Zlatibor, 9.07.2017, N43 47.310 E19 43.721, 662 m. lgt. Toševski	Serbia	*Prunus cerasifer* *Pyrus* sp.	mon1_Ef (PV943825)	**10**
4538	*R. monzinii*	Serbia, Mt. Zlatibor, 9.07.2017, N43 47.310 E19 43.721, 662 m, lgt. Toševski	Serbia	*Prunus sp.* *Pyrus* sp.		
4888	*R. monzinii*	Serbia, Brusnik, Negotin, N44 6.489 E22 24.115, 322 m, 21.05.2018, lgt. Toševski	Serbia	*Prunus spinosa*		
4890	*R. monzinii*	Serbia, Brusnik, Negotin, N44 6.489 E22 24.115, 322 m, 21.05.2018, lgt. Toševski	Serbia	*Prunus spinosa*		
5174	*R. monzinii*	Italy, Liguria, (GE), Piani di Creto, 23.06.2018 lgt. L. Diotti	Italy	-		
5997	*R. monzinii*	Italy, Liguria, (GE), Piani di Creto, 23.06.2018 lgt. L. Diotti	Italy	-		
5999	*R. monzinii*	Italy, Liguria, (GE), Piani di Creto, 23.06.2018 lgt. L. Diotti	Italy	-		
6000	*R. monzinii*	Italy, Liguria, (GE), Piani di Creto, 23.06.2018 lgt. L. Diotti	Italy	-		
6001	*R. monzinii*	Italy, Liguria, (GE), Piani di Creto, 23.06.2018 lgt. L. Diotti	Italy	-		
6003	*R. monzinii*	Italy, Liguria, (GE), Piani di Creto, 23.06.2018 lgt. L. Diotti	Italy	-		
4539	*R. monzinii*	Serbia, Mt. Zlatibor, 9.07.2017, N43 47.310 E19 43.721, 662 m, lgt. Toševski	Serbia	*Prunus sp.* & *Pyrus* sp.	mon2_Ef (PV943826)	**10**
4881	*R. monzinii*	Serbia, Brusnik, Negotin, N44 6.489 E22 24.115, 322 m, 21.05.2018, lgt. Toševski	Serbia	*Prunus spinosa*		

4885	*R. monzinii*	Serbia, Brusnik, Negotin, N44 6.489 E22 24.115, 322 m., 21.05.2018, lgt. Toševski	Serbia	*Prunus spinosa*		
4889	*R. monzinii*	Serbia, Brusnik, Negotin, N44 6.489 E22 24.115, 322 m, 21.05.2018, lgt. Toševski	Serbia	*Prunus spinosa*		
4893	*R. monzinii*	Serbia, Vlasina, Božički Kanal, N42 40.997 E22 21.888, 1289 m, 21.06.2018, lgt. Toševski	Serbia	*Prunus cerasifera*		
4894	*R. monzinii*	Serbia, Vlasina, Božički Kanal, N42 40.997 E22 21.888, 1289 m, 21.06.2018, lgt. Toševski	Serbia	*Prunus cerasifera*		
5176	*R. monzinii*	Italy, Liguria, (GE), Piani di Creto, 23.06.2018 lgt. L. Diotti	Italy	-		
5258	*R. monzinii*	Serbia, Brusnik, Negotin, N44 6.489 E22 24.115, 322 m, 21.05.2018, lgt. Toševski	Serbia	*Prunus spinosa*		
5998	*R. monzinii*	Italy, Liguria, (GE), Piani di Creto, 23.06.2018 lgt. L. Diotti	Italy	-		
6002	*R. monzinii*	Italy, Liguria, (GE), Piani di Creto, 23.06.2018 lgt. L. Diotti	Italy	-		
4897	*R. monzinii*	Serbia, Vranje, Devotin, N42 36.511 E21 52.252, 991 m, 22.06.2018, lgt. Toševski	Serbia	*Prunus spinosa*	mon3_Ef (PV943827)	**1**
5175	*R. monzinii*	Italy, Liguria, (GE), Piani di Creto, 23.06.2018 lgt. L. Diotti	Italy	-	mon4_Ef (PV943797)	**1**
6585	*R. monzinii*	Greece, Arethousa, 23.06.2023, Macedonia, N40 44.299 E23 34.854, lgt. Toševski	Greece	*Prunus spinosa*	mon5_Ef (PV943828)	**5**
6586	*R. monzinii*	Greece, Arethousa, 23.06.2023, Macedonia, N40 44.299 E23 34.854, lgt. Toševski	Greece	*Prunus spinosa*		
6587	*R. monzinii*	Greece, Arethousa, 23.06.2023, Macedonia, N40 44.299 E23 34.854, lgt. Toševski	Greece	*Prunus spinosa*		
6589	*R. monzinii*	Greece, Arethousa, 23.06.2023, Macedonia,	Greece	*Prunus spinosa*		

		N40 44.299 E23 34.854, lgt. Toševski				
6590	*R. monzinii*	Greece, Arethousa, 23.06.2023, Macedonia, N40 44.299 E23 34.854, lgt. Toševski	Greece	*Prunus spinosa*		
6588	*R. monzinii*	Greece, Arethousa, 23.06.2023, Macedonia, N40 44.299 E23 34.854, lgt. Toševski	Greece	*Prunus spinosa*	mon6_Ef (PV943829)	**2**
6591	*R. monzinii*	Greece, Arethousa, 23.06.2023, Macedonia, N40 44.299 E23 34.854, lgt. Toševski	Greece	*Prunus spinosa*		
4906	*R. monzinii*	Greece, Mt. Taygetos 10.07.2018, N37 04.155 E22 15.882, lgt. Toševski	Greece	*Prunus cerasifera*	mon7_Ef (PV943830)	**1**
						**30**

***Rhamphus diottii* sp. n. Toševski & Caldara**						
**Voucher DNA**	**Species**	**Location**	**Origin**	**Host**	**Ef haplotype name (accession number)**	**Frequency**
4409	*R. diottii* sp.n. PARATYPE	Serbia, Slankamen Vinogradi, 29.05.2009, N45 9.715 E20 11.750, 224 m., lgt. Toševski	Serbia	*Prunus spinosa*	dio1_Ef (PV943831)	**9**
4542	*R. diottii* sp.n. PARATYPE	Serbia, Slankamen Vinogradi, 10.06.2017, N45 9.715 E20 11.750, 224 m., lgt. Toševski	Serbia	*Prunus spinosa*		
5262	*R. diottii* sp.n. PARATYPE	Serbia, Slankamen Vinogradi, 29.05.2009, N45 9.715 E20 11.750, 224 m., lgt. Toševski	Serbia	*Prunus spinosa*		
5263	*R. diottii* sp.n. PARATYPE	Serbia, Slankamen Vinogradi, 29.05.2009, N45 9.715 E20 11.750, 224 m., lgt. Toševski	Serbia	*Prunus spinosa*		
5264	*R. diottii* sp.n. PARATYPE	Serbia, Slankamen Vinogradi, 29.05.2009, N45 9.715 E20 11.750, 224 m., lgt. Toševski	Serbia	*Prunus spinosa*		
5265	*R. diottii* sp.n. PARATYPE	Serbia, Slankamen Vinogradi, 10.06.2017, N45 9.715 E20 11.750, 224 m., lgt. Toševski	Serbia	*Prunus spinosa*		

5268	*R. diottii* sp.n. PARATYPE	Serbia, Slankamen Vinogradi, 10.06.2017, N45 9.715 E20 11.750, 224 m., lgt. Toševski	Serbia	*Prunus spinosa*		
5269	*R. diottii* sp.n. PARATYPE	Serbia, Slankamen Vinogradi, 29.05.2009, N45 9.715 E20 11.750, 224 m., lgt. Toševski	Serbia	*Prunus spinosa*		
5270	*R. diottii* sp.n. **HOLOTYPUS**	Serbia, Slankamen Vinogradi, 29.05.2009, N45 9.715 E20 11.750, 224 m., lgt. Toševski	Serbia	*Prunus spinosa*		
4410	*R. diottii* sp.n. PARATYPE	Serbia, Slankamen Vinogradi, 29.05.2009, N45 9.715 E20 11.750, 224 m., lgt. Toševski	Serbia	*Prunus spinosa*	dio2_Ef (PV943832)	**2**
5267	*R. diottii* sp.n. PARATYPE	Serbia, Slankamen Vinogradi, 10.06.2017, N45 9.715 E20 11.750, 224 m., lgt. Toševski	Serbia	*Prunus spinosa*		
5266	*R. diottii* sp.n. PARATYPE	Serbia, Slankamen Vinogradi, 10.06.2017, N45 9.715 E20 11.750, 224 m., lgt. Toševski	Serbia	*Prunus spinosa*	dio3_Ef (PV943833)	**1**
						**12**

***Rhamphus ibericus* sp. n. Toševski & Caldara**						
**Voucher DNA**	**Species**	**Location**	**Origin**	**Host**	**Ef haplotype name (accession number)**	**Frequency**
6593	*R. ibericus sp.n.* PARATYPE	España, País Vasco, Araba/Álava, Elburgo-Burgelu, 556 m, N 42.812 W 2.633, 15.06.2023, lgt. Iñigo Ugarte San Vicente & Fernando Salgueira	Spain	*Prunus spinosa*	ibe1_Ef (PV943834)	**3**
6594	*R. ibericus sp.n.* PARATYPE	España, País Vasco, Araba/Álava, Elburgo-Burgelu, 556 m, N 42.812 W 2.633, 15.06.2023, lgt. Iñigo Ugarte San Vicente & Fernando Salgueira	Spain	*Prunus spinosa*		
6598	*R. ibericus sp.n.* **HOLOTYPUS**	España, País Vasco, Araba/Álava, Elburgo-Burgelu, 556 m, N 42.812 W 2.633, 15.06.2023, lgt. Iñigo Ugarte San Vicente & Fernando Salgueira	Spain	*Prunus spinosa*		

						**3**

Rhamphus subaeneus Illiger, 1808

Voucher DNA	Species	Location	Origin	Host	Ef haplotype name (accession number)	Frequency
5331	*R. subaeneus*	Spain, San Vicente de Arana, La Dehesa Álava, 8.07.2018, lgt. Iñigo Ugarte San Vicente & Fernando Salgueira	Spain	*Crataegus monogyna*	sub1_Ef (PV943798)	**1**
5337	*R. subaeneus*	Spain, San Vicente de Arana, La Dehesa, Álava, 7.07.2018, lgt. Iñigo Ugarte San Vicente & Fernando Salgueira	Spain	*Crataegus monogyna*	sub2_Ef (PV943799)	**2**
6094	*R. subaeneus*	Czechia, Bohemia Centr., Chramosty, Brdce hill, N49°40'12", E14°19'57", 415 m., 21.05.2020, lgt J. Kratky	Czechia	*Crataegus* sp.		
5336	*R. subaeneus*	Spain, San Vicente de Arana, La Dehesa, Álava, 7.07.2018, lgt. Iñigo Ugarte San Vicente & Fernando Salgueira	Spain	*Crataegus monogyna*	sub3_Ef (PV943800)	**2**
5338	*R. subaeneus*	Spain, San Vicente de Arana, La Dehesa, Álava, 7.07.2018, lgt. Iñigo Ugarte San Vicente & Fernando Salgueira	Spain	*Crataegus monogyna*		
6601	*R. subaeneus*	España, País Vasco, provincia de Araba/Álava, Subijana de Álava, 518 m s.n.m., 25-VI-2019, lgt. Iñigo Ugarte San Vicente & Fernando Salgueira	Spain	Crataegus monogyna	sub4_Ef (PV943801)	**1**
						**6**

Rhamphus cerdanicus Tempère, 1982

Voucher DNA	Species	Location	Origin	Host	Ef haplotype name (accession number)	Frequency

6595	*R. cerdanicus*	España, País Vasco, Araba/Álava, Elburgo-Burgelu, 556 m, N 42.812 W 2.633, 15.06.2023, lgt. Iñigo Ugarte San Vicente & Fernando Salgueira	Spain	*Prunus spinosa*	cer1_Ef (PV943835)	**2**
6596	*R. cerdanicus*	España, País Vasco, Araba/Álava, Elburgo-Burgelu, 556 m, N 42.812 W 2.633, 15.06.2023, lgt. Iñigo Ugarte San Vicente & Fernando Salgueira	Spain	*Prunus spinosa*		
6597	*R. cerdanicus*	España, País Vasco, Araba/Álava, Elburgo-Burgelu, 556 m, N 42.812 W 2.633, 15.06.2023, lgt. Iñigo Ugarte San Vicente & Fernando Salgueira	Spain	*Prunus spinosa*	cer2_Ef (PV943836)	**1**
						**3**

Rhamphus loebli Germann & Colonnelli, 2018

Voucher DNA	Species	Location	Origin	Host	Ef haplotype name (accession number)	Frequency
6995	*R. loebli*	Spain, E. Castilia, Srr. Francia, La Alberca, env., 1075 m, 40°31'49"N, 06°08'44"W, 25.05.2019, lgt. Krátký	Spain	*Halimium sp.*	loe1_Ef (PV943802)	**2**
7352	*R. loebli*	Spain, E. Andalucia, 4 km N of Rociana del Condado, 37.344 N 6.596 W, 10.03.2011, lgt. J. Kratky	Spain	*Halimium sp.*		
						**2**

Rhamphus hisamatsui Chûjô & Morimoto, 1960

Voucher DNA	Species	Location	Origin	Host	Ef haplotype name (accession number)	Frequency

6328	*R. hisamatsui*	Japan, Inugoeji forest, Yamakita town, Kanagawa pref., 28.06.2021, lgt. Y. Notsu	Japan	*Acer pictum* ssp. *mono*	his1_Ef (PV943803)	**7**
6329	*R. hisamatsui*	Japan, Inugoeji forest, Yamakita town, Kanagawa pref., 28.06.2021, lgt. Y. Notsu	Japan	*Acer pictum* ssp. *mono*		
6330	*R. hisamatsui*	Japan, Inugoeji forest, Yamakita town, Kanagawa pref., 28.06.2021, lgt. Y. Notsu	Japan	*Acer pictum* ssp. *mono*		
6331	*R. hisamatsui*	Japan, Inugoeji forest, Yamakita town, Kanagawa pref., 28.06.2021, lgt. Y. Notsu	Japan	*Acer pictum* ssp. *mono*		
6332	*R. hisamatsui*	Japan, Inugoeji forest, Yamakita town, Kanagawa pref., 28.06.2021, lgt. Y. Notsu	Japan	*Acer pictum* ssp. *mono*		
6333	*R. hisamatsui*	Japan, Inugoeji forest, Yamakita town, Kanagawa pref., 28.06.2021, lgt. Y. Notsu	Japan	*Acer pictum* ssp. *mono*		
6335	*R. hisamatsui*	Japan, Mikuni pass., Yamanakako vlg., Yamanashi pref., 12.06.2021, lgt. Y. Notsu	Japan	*Acer pictum* ssp. *mono*		
						**7**
						**229**
