# Supplementary material for: An Integrative Revision of the Genus Rhamphus (Curculionidae) from the Western Palearctic: Morphological and Molecular Data Reveal the Radiation of Multiple Species
Source: Insects. 2025 Nov 3;16(11):1123. doi: 10.3390/insects16111123 (PMC12653807; doi:10.3390/insects16111123)
Supplement: Supplementary file 1 [file insects-16-01123-s001.zip › Table_S3.pdf]

Table S3. List of *Rhamphus* specimens sequenced for *carbamoyl phosphate synthetase* 2 (*CAD*) gene, sorted by DNA voucher code, species name, locality, country of origin, host plant affiliation, haplotype name with accession number in NCBI GenBank and frequency.

***Rhamphus oxyacanthae* (Marsham, 1802)**						
**Voucher DNA**	**Species**	**Location**	**Origin**	**Host**	**CAD haplotype name (accession number)**	**Frequency**
4534	*R. oxyacanthae*	Serbia, Mt. Zlatibor, 9.07.2017, N43 47.310 E19 43.721, 662 m, lgt. Toševski	Serbia	*Crataegus sp.*	oxy1_CAD (PV930506)	**26**
4856	*R. oxyacanthae*	Serbia, Brusnik, Negotin, N44 6.489 E22 24.115, 322 m, 21.05.2018, lgt. Toševski	Serbia	*Cydonia oblonga*		
4860	*R. oxyacanthae*	Serbia, Brusnik, Negotin, N44 6.489 E22 24.115, 322 m, 21.05.2018, lgt. Toševski	Serbia	*Pyrus sp.*		
4891	*R. oxyacanthae*	Serbia, Vlasina, N42 40.573 E22 18.818, 1246 m, 21.06.2018, lgt. Toševski	Serbia	*Crataegus sp.*		
4892	*R. oxyacanthae*	Serbia, Vlasina, Božićki Kanal, N42 40.997 E22 21.888, 1289 m, 21.06.2018, lgt. Toševski	Serbia	*Prunus cerasifera*		
4895	*R. oxyacanthae*	Serbia, Vlasina, Božićki Kanal, N42 40.997 E22 21.888, 1289 m, 21.06.2018, lgt. Toševski	Serbia	*Prunus cerasifera*		
4907	*R. oxyacanthae*	Greece, Mt. Taygetos 10.07.2018, N37 04.155 E22 15.882, 1381 m, lgt. Toševski	Greece	*Pyrus spinosa*		
4908	*R. oxyacanthae*	Greece, Mt. Taygetos 10.07.2018, N37 04.155 E22 15.882, 1381 m, lgt. Toševski	Greece	*Pyrus spinosa*		
5181	*R. oxyacanthae*	Italy, Fino Mornasco, (CO), Ville dei Mulini, 25.05.2018, lgt. L. Diotti	Italy	no data		
5182	*R. oxyacanthae*	Italy, Fino Mornasco, (CO), Ville dei Mulini, 25.05.2018, lgt. L. Diotti	Italy	no data		
5271	*R. oxyacanthae*	Greece, Mt. Taygetos 10.07.2018, N37 04.155 E22 15.882, 1381 m, lgt. Toševski	Greece	*Pyrus spinosa*		
5272	*R. oxyacanthae*	Greece, Mt. Taygetos 10.07.2018, N37 04.155 E22 15.882, 1381 m, lgt. Toševski	Greece	*Pyrus spinosa*		

5273	*R. oxyacanthae*	Greece, Mt. Taygetos 10.07.2018, N37 04.155 E22 15.882, 1381 m, lgt. Toševski	Greece	*Pyrus spinosa*		
5274	*R. oxyacanthae*	Greece, Mt. Taygetos 10.07.2018, N37 04.155 E22 15.882, 1381 m, lgt. Toševski	Greece	*Pyrus spinosa*		
5275	*R. oxyacanthae*	Greece, Mt. Taygetos 10.07.2018, N37 04.155 E22 15.882, 1381 m, lgt. Toševski	Greece	*Pyrus spinosa*		
5332	*R. oxyacanthae*	Spain, San Vicente sa de logiz, Álava, 8.07.2018, lgt. Iñigo Ugarte San Vicente & Fernando Salgueira	Spain	*Crataegus monogyna*		
5335	*R. oxyacanthae*	Spain, San Vicente de Arana, La Dehesa Álava, 8.07.2018, lgt. Iñigo Ugarte San Vicente & Fernando Salgueira	Spain	*Crataegus monogyna*		
5345	*R. oxyacanthae*	Greece, Mt. Taygetos 10.07.2018, N37 04.155 E22 15.882, 1381 m, lgt. Toševski	Greece	*Pyrus spinosa*		
5346	*R. oxyacanthae*	Greece, Mt. Taygetos 10.07.2018, N37 04.155 E22 15.882, 1381 m, lgt. Toševski	Greece	*Pyrus spinosa*		
6323	*R. oxyacanthae*	Italy, Sila Grande, (CS) Lago di Cecita, 17.06.2015, lgt. L. Diotti	Italy	no data		
6324	*R. oxyacanthae*	Italy, Sila Grande, (CS) Lago di Cecita, 17.06.2015, lgt. L. Diotti	Italy	no data		
6325	*R. oxyacanthae*	Italy, Sila Grande, (CS) Lago di Cecita, 17.06.2015, lgt. L. Diotti	Italy	no data		
6326	*R. oxyacanthae*	Italy, Sila Grande, (CS) Lago di Cecita, 17.06.2015, lgt. L. Diotti	Italy	no data		
6327	*R. oxyacanthae*	Italy, Sila Grande, (CS) Lago di Cecita, 17.06.2015, lgt. L. Diotti	Italy	no data		
6350	*R. oxyacanthae*	Serbia, Vratarnica, Zaječar, 15.06.2021, N43 47.630 E22 18.125, 165 m., lgt. Toševski	Serbia	*Malus domestica*		
7382	*R. oxyacanthae*	Greece, Macedonia, Arethousa, 13.06.2024, N40 44.134 E23 36.193, 374 m. lgt. Toševski	Greece	*Crataegus sp.*		
4537	*R. oxyacanthae*	Serbia, Mt. Zlatibor, 9.07.2017, N43 47.310 E19 43.721, 662 m, lgt. Toševski	Serbia	*Crataegus sp.*	oxy2_CAD (PV930507)	**13**
4854	*R. oxyacanthae*	Serbia, Pitot, Staničenje, 20.05.2018, N43 13.020	Serbia	*Crataegus sp.*		

		E22 30.556, 403 m, lgt. Toševski				
5953	*R. oxyacanthae* (NEOTYPE)	England, Suffolk, TL7086, 23.06.2020., lgt. H. Mandel	England	*Crataegus sp.*		
5954	*R. oxyacanthae*	England, Suffolk, TL7086, 23.06.2020., lgt. H. Mandel	England	*Crataegus sp.*		
5955	*R. oxyacanthae*	England, Suffolk, TL7086, 23.06.2020., lgt. H. Mandel	England	*Crataegus sp.*		
5956	*R. oxyacanthae*	England, Suffolk, TL7086, 23.06.2020., lgt. H. Mandel	England	*Crataegus sp.*		
5957	*R. oxyacanthae*	England, Suffolk, TL7086, 23.06.2020., lgt. H. Mandel	England	*Crataegus sp.*		
5958	*R. oxyacanthae*	England, Suffolk, TL7086, 23.06.2020., lgt. H. Mandel	England	*Crataegus sp.*		
5959	*R. oxyacanthae*	England, Suffolk, TL7086, 23.06.2020., lgt. H. Mandel	England	*Crataegus sp.*		
6012	*R. oxyacanthae*	F. 33, Queyrac, Le Gross Cap, 4.05.2018, lgt. Lessieur David	France	*Crataegus monogyna*		
6013	*R. oxyacanthae*	F. 33, Queyrac, Le Gross Cap, 4.05.2018, lgt. Lessieur David	France	*Crataegus monogyna*		
6014	*R. oxyacanthae*	F. 33, Queyrac, Le Gross Cap, 4.05.2018, lgt. Lessieur David	France	*Crataegus monogyna*		
6015	*R. oxyacanthae*	F. 33, Queyrac, Le Gross Cap, 4.05.2018, lgt. Lessieur David	France	*Crataegus monogyna*		
5159	*R. oxyacanthae*	Italy, Giaglione, (TO), Val Clarea, 7.07.2018, lgt. L. Diotti	Italy	no data	oxy3_CAD (PV930483)	**6**
5169	*R. oxyacanthae*	Italy, Basilicata, bosco di Accettura, 10.06.2018, lgt. L. Diotti	Italy	no data		
5171	*R. oxyacanthae*	Italy, Basilicata, bosco di Accettura, 10.06.2018, lgt. L. Diotti	Italy	no data		
5172	*R. oxyacanthae*	Italy, Muro Lucano (PT), Monte Paratiello, 10.06.2018, lgt. L. Diotti	Italy	no data		
5177	*R. oxyacanthae*	Italy, Emilia (Parma), Passo Cirrone 225 m, 8.08.2018, lgt. L. Diotti	Italy	no data		
5178	*R. oxyacanthae*	Italy, Emilia (Parma), Passo Cirrone 225 m, 8.08.2018, lgt. L. Diotti	Italy	no data		
5160	*R. oxyacanthae*	Italy, Giaglione, (TO), Val Clarea, 7.07.2018, lgt. L. Diotti	Italy	no data	oxy4_CAD (PV930484)	**4**
5161	*R. oxyacanthae*	Italy, Giaglione, (TO), Val Clarea, 7.07.2018, lgt. L. Diotti	Italy	no data		
5168	*R. oxyacanthae*	Italy, Basilicata, bosco di Accettura, 10.06.2018, lgt. L. Diotti	Italy	no data		

5173	*R. oxyacanthae*	Italy, Muro Lucano (PT), Monte Paratiello, 10.06.2018, lgt. L. Diotti	Italy	no data		
5170	*R. oxyacanthae*	Italy, Basilicata, bosco di Accettura, 10.06.2018, lgt. L. Diotti	Italy	no data	oxy5_CAD (PV930485)	**1**
6602	*R. oxyacanthae*	Spain, País Vasco, prov. de Araba/ Álava, Subijana de Álava, 518 m s.n.m., 25.06.2019, lgt. Iñigo Ugarte San Vicente & Fernando Salgueira	Spain	*Crataegus monogyna*	oxy6_CAD (PV930486)	**1**
						**51**

Rhamphus bavierai **Diotti, Caldara & Toševski, 2021**

**Voucher DNA**	**Species**	**Location**	**Origin**	**Host**	**CAD haplotype name (accession number)**	**Frequency**
4403	*R. bavierai*	Sicily, Piano Battaglia, 1600 m., 15.07.2016, lgt. Baviera C.	Sicily	*Crataegus monogyna, C. laciniata*	bav1_CAD (PV930487)	**21**
4404	*R. bavierai*	Sicily, Piano Battaglia, 1600 m., 15.07.2016, lgt Baviera C.	Sicily	*Crataegus monogyna, C. laciniata*		
5960	*R. bavierai*	Sicily, Palermo: Madonie, P. Zucchi, 1100 m a.s.l., 16.06.2020, lgt. Baviera C	Sicily	*Crataegus monogyna, C. laciniata*		
5961	*R. bavierai*	Sicily, Palermo: Madonie, P. Zucchi, 1100 m a.s.l., 16.06.2020, lgt. Baviera C.	Sicily	*Crataegus monogyna, C. laciniata*		
5965	*R. bavierai*	Sicily, Palermo: Madonie, P. zucchi, 1100 m a.s.l., 16.06.2020, lgt. Baviera C.	Sicily	*Crataegus monogyna, C. laciniata*		
5966	*R. bavierai*	Sicily, Catania: Etna 1200 m., Linguaglossa, 27.06.2020, lgt. Baviera C.	Sicily	*Crataegus monogyna, C. laciniata*		
5970	*R. bavierai*	Sicily, Catania: Etna 1200 m., Linguaglossa, 27.06.2020, lgt. Baviera C.	Sicily	*Crataegus monogyna, C. laciniata*		
5971	*R. bavierai*	Sicily, Catania: Etna 1200 m., Linguaglossa, 27.06.2020 lgt. Baviera C.	Sicily	*Crataegus monogyna, C. laciniata*		
5972	*R. bavierai*	Sicily, Catania: Etna 1200 m., Linguaglossa, 27.06.2020 lgt. Baviera C.	Sicily	*Crataegus monogyna, C. laciniata*		

5979	*R. bavierai*	Sicily, Messina: Nebrodi Caronia, 1400 m., 20.06.2020, lgt. Baviera C.	Sicily	*Crataegus monogyna, C. laciniata*		
5980	*R. bavierai*	Sicily, Messina: Nebrodi Caronia, 1400 m., 20.06.2020, lgt. Baviera C.	Sicily	*Crataegus monogyna, C. laciniata*		
5981	*R. bavierai*	Sicily, Messina: Nebrodi Caronia, 1400 m., 20.06.2020, lgt. Baviera C.	Sicily	*Crataegus monogyna, C. laciniata*		
5982	*R. bavierai*	Sicily, Messina: Nebrodi Caronia, 1400 m., 20.06.2020, lgt. Baviera	Sicily	*Crataegus monogyna, C. laciniata*		
5983	*R. bavierai*	Sicily, Messina: Nebrodi Caronia, 1400 m., 20.06.2020, lgt. Baviera C.	Sicily	*Crataegus monogyna, C. laciniata*		
5984	*R. bavierai*	Sicily, Messina: Nebrodi Caronia, 1400 m., 20.06.2020, lgt. Baviera C.	Sicily	*Crataegus monogyna, C. laciniata*		
5985	*R. bavierai*	Sicily, Messina: Nebrodi Caronia, 1400 m., 20.06.2020, lgt. Baviera C.	Sicily	*Crataegus monogyna, C. laciniata*		
5986	*R. bavierai*	Sicily, Messina: Nebrodi Caronia, 1400 m., 20.06.2020, lgt. Baviera C.	Sicily	*Crataegus monogyna, C. laciniata*		
5988	*R. bavierai*	Sicily, Messina: Nebrodi, Capizzi, 1450 m, Portella Obolo, 20.06.2020, lgt. Baviera C.	Sicily	*Crataegus monogyna, C. laciniata*		
5989	*R. bavierai*	Sicily, Messina: Nebrodi, Capizzi, 1450 m, Portella Obolo, 20.06.2020, lgt. Baviera C.	Sicily	*Crataegus monogyna, C. laciniata*		
6004	*R. bavierai*	Sicily, Piano Battaglia, 1600 m., 15.07.2016, Lgt. Baviera C.	Sicily	*Crataegus monogyna, C. laciniata*		
6005	*R. bavierai*	Sicily, Piano Battaglia, 1600 m., 15.07.2016, Lgt. Baviera C.	Sicily	*Crataegus monogyna, C. laciniata*		
5987	*R. bavierai*	Sicily, Messina: Nebrodi, Capizzi, 1450 m, Portella Obolo, 20.06.2020 lgt. Baviera C.	Sicily	*Crataegus monogyna, C. laciniata*	bav2_CAD (PV930488)	**1**
						**22**

Rhamphus hampsicora **Diotti, Caldara & Toševski, 2021**						
**Voucher DNA**	**Species**	**Location**	**Origin**	**Host**	**CAD haplotype name (accession number)**	**Frequency**
5339	*R. hampsicora*	Sardinia, Siniscola (NU), Pandici Monte Albo, 25.05.2012, lgt. Diotti	Sardinia	*Prunus* sp.	ham1_CAD (PV930489)	**3**
5341	*R. hampsicora*	Sardinia, Siniscola (NU), Pandici Monte Albo, 25.05.2012, lgt. Diotti	Sardinia	*Prunus* sp.		
5342	*R. hampsicora*	Sardinia, Siniscola (NU), Pandici Monte Albo, 25.05.2012, lgt. Diotti	Sardinia	*Prunus* sp.		
5340	*R. hampsicora*	Sardinia, Siniscola (NU), Pandici Monte Albo, 25.05.2012, lgt. Diotti	Sardinia	*Prunus* sp.	ham2_CAD (PV930490)	**1**
						**4**

Rhamphus cypricus **sp. n. Toševski & Caldara**						
**Voucher DNA**	**Species**	**Location**	**Origin**	**Host**	**CAD haplotype name (accession number)**	**Frequency**
6178	*R. cypricus* n.sp. PARATYPE	Cyprus, Paphos p., Drouseia env., 541 m. 23.03.2019, lgt. F. Pavel	Cyprus	no data	-	-
6578	*Rhamphus cypricus* n.sp. PARATYPE	Cyprus, Kissousa (Limnassol), 570 m, 23.04.2010	Cyprus	no data	-	-
7346	*R. cypricus* sp.n. PARATYPE	Cyprus, Droushia-Ineia(Paphos, Chypre) 600m, 12.04.2005, G. et H. Alziar lgt. sur Crataegus fleuri. Collection G. Alziar	Cyprus	*Crataegus* sp.	-	-
7349	*R. cypricus* sp.n. PARATYPE	Cyprus, Droushia-Ineia(Paphos, Chypre) 600m, 12.04.2005, G. et H. Alziar lgt. sur Crataegus fleuri. Collection G. Alziar	Cyprus	*Crataegus* sp.	-	-
7371	*R. cypricus* sp.n. PARATYPE	Cyprus, Parsata, 7.04.2024, 34.8281745 N, 33.2648008 E, lgt. L. Forbicioni	Cyprus	*Crataegus azarolus*	cyp1_CAD (PV930508)	**5**
7372	*R. cypricus* sp.n. **HOLOTYPE**	Cyprus, Parsata, 7.04.2024, 34.8281745 N, 33.2648008 E, lgt. L. Forbicioni	Cyprus	*Crataegus azarolus*		
7374	*R. cypricus* sp.n. PARATYPE	Cyprus, Parsata, 7.04.2024, 34.8281745 N, 33.2648008 E, lgt. L. Forbicioni	Cyprus	*Crataegus azarolus*		
7377	*R. cypricus* sp.n. PARATYPE	Cyprus, Parsata, 7.04.2024, 34.8281745 N, 33.2648008 E, lgt. L. Forbicioni	Cyprus	*Crataegus azarolus*		

7378	*R. cypricus* sp.n. PARATYPE	Cyprus, Parsata, 7.04.2024, 34.8281745 N, 33.2648008 E, lgt. L. Forbicioni	Cyprus	*Crataegus azarolus*		
7376	*R. cypricus* sp.n. PARATYPE	Cyprus, Parsata, 7.04.2024, 34.8281745 N, 33.2648008 E, lgt. L. Forbicioni	Cyprus	*Crataegus azarolus*	cyp2_CAD (PV930509)	**1**
7373	*R. cypricus* sp.n. PARATYPE	Cyprus, Parsata, 7.04.2024, 34.8281745 N, 33.2648008 E, lgt. L. Forbicioni	Cyprus	*Crataegus azarolus*	cyp3_CAD (PV930510)	**1**
7375	*R. cypricus* sp.n. PARATYPE	Cyprus, Parsata, 7.04.2024, 34.8281745 N, 33.2648008 E, lgt. L. Forbicioni	Cyprus	*Crataegus azarolus*	cyp4_CAD (PV930511)	**1**
7379	*R. cypricus* sp.n. PARATYPE	Cyprus, Lefkara, 12.04.2024, 34.8692177 N, 33.2839350 E, lgt. L. Forbicioni	Cyprus	*Crataegus azarolus*	cyp5_CAD (PV930512)	**2**
7380	*R. cypricus* sp.n. PARATYPE	Cyprus, Lefkara, 12.04.2024, 34.8692177 N, 33.2839350 E, lgt. L. Forbicioni	Cyprus	*Crataegus azarolus*		
						**10**

Rhamphus macedonicus **sp. n. Toševski & Caldara**

Voucher DNA	Species	Location	Origin	Host	CAD haplotype name (accession number)	Frequency
6582	*R. macedonicus* sp.n. HOLOTYPE	Greece, Macedonia, Arethousa, 23.06.2023, N40 44.134 E23 36.193, 374 m. lgt. Toševski	Greece	*Crataegus* sp.	mac1_CAD (PV930513)	**15**
6583	R. macedonicus sp.n. PARATYPE	Greece, Macedonia, Arethousa, 23.06.2023, N40 44.134 E23 36.193, 374 m. lgt. Toševski	Greece	*Crataegus* sp.		
6584	R. macedonicus sp.n. PARATYPE	Greece, Macedonia, Arethousa, 23.06.2023, N40 44.134 E23 36.193, 374 m. lgt. Toševski	Greece	*Crataegus* sp.		
7381	R. macedonicus sp.n. PARATYPE	Greece, Macedonia, Arethousa, 13.06.2024, N40 44.134 E23 36.193, 374 m. lgt. J. Jović	Greece	*Crataegus* sp.		
7383	R. macedonicus sp.n. PARATYPE	Greece, Macedonia, Arethousa, 13.06.2024, N40 44.134 E23 36.193, 374 m. lgt. J. Jović	Greece	*Crataegus* sp.		
7384	R. macedonicus sp.n. PARATYPE	Greece, Macedonia, Arethousa, 13.06.2024, N40 44.134 E23 36.193, 374 m. lgt. J. Jović	Greece	*Crataegus* sp.		
7385	R. macedonicus sp.n. PARATYPE	Greece, Macedonia, Arethousa, 13.06.2024, N40 44.134 E23 36.193, 374 m. lgt. J. Jović	Greece	*Crataegus* sp.		

7386	R. macedonicus sp.n. PARATYPE	Greece, Macedonia, Arethousa, 13.06.2024, N40 44.134 E23 36.193, 374 m. lgt. J. Jović	Greece	*Crataegus* sp.		
7387	R. macedonicus sp.n. PARATYPE	Greece, Macedonia, Arethousa, 13.06.2024, N40 44.134 E23 36.193, 374 m. lgt. J. Jović	Greece	*Crataegus* sp.		
7388	R. macedonicus sp.n. PARATYPE	Greece, Macedonia, Arethousa, 13.06.2024, N40 44.134 E23 36.193, 374 m. lgt. J. Jović	Greece	*Crataegus* sp.		
7389	R. macedonicus sp.n. PARATYPE	Greece, Macedonia, Arethousa, 13.06.2024, N40 44.134 E23 36.193, 374 m. lgt. J. Jović	Greece	*Crataegus* sp.		
7390	R. macedonicus sp.n. PARATYPE	Greece, Macedonia, Arethousa, 13.06.2024, N40 44.134 E23 36.193, 374 m. lgt. J. Jović	Greece	*Crataegus* sp.		
7391	R. macedonicus sp.n. PARATYPE	Greece, Macedonia, Arethousa, 13.06.2024, N40 44.134 E23 36.193, 374 m. lgt. J. Jović	Greece	*Crataegus* sp.		
7392	R. macedonicus sp.n. PARATYPE	Greece, Macedonia, Arethousa, 13.06.2024, N40 44.134 E23 36.193, 374 m. lgt. J. Jović	Greece	*Crataegus* sp.		
7393	R. macedonicus sp.n. PARATYPE	Greece, Macedonia, Arethousa, 13.06.2024, N40 44.134 E23 36.193, 374 m. lgt. J. Jović	Greece	*Crataegus* sp.		
						**15**

***Rhamphus pulicarius* (Herbst, 1795)**

**Voucher DNA**	**Species**	**Location**	**Origin**	**Host**	**CAD haplotype name (accession number)**	**Frequency**
4540	*R. pulicarius*	Serbia, Draglica, Mt. Zlatar, N43 35.173 E19 43.645, 929 m., 12.07.2017, lgt. Toševski	Serbia	*Salix caprea*	pul1_CAD (PV930514)	**11**
4579	*R. pulicarius*	Serbia, Draglica, Mt. Zlatar, N43 35.173 E19 43.645, 929 m., 12.07.2017, lgt. Toševski	Serbia	*Salix caprea*		
4580	*R. pulicarius*	Serbia, Draglica, Mt. Zlatar, N43 35.173 E19 43.645, 929 m., 12.07.2017, lgt. Toševski	Serbia	*Salix caprea*		
4583	*R. pulicarius*	Serbia, Babin Zub, Stara Planina, N43 23.165 E22 35.549, 1250 m, 5.07.2017, lgt. Toševski	Serbia	*Salix caprea*		

4585	*R. pulicarius*	Serbia, Babin Zub, Stara Planina, N43 23.165 E22 35.549, 1250 m, 5.07.2017, lgt. Toševski	Serbia	*Salix caprea*		
5179	*R. pulicarius*	Italy, Lombardia (CO), dintorni Monguzzo, 7.06.2017, lgt. L. Diotti	Italy	no data		
5180	*R. pulicarius*	Italy, Lombardia (CO), dintorni Monguzzo, 7.06.2017, lgt. L. Diotti	Italy	no data		
6226	*R. pulicarius*	Poland, Rudnik ad Lublin, 51°16'58.8"N 22°38'28.8"E, 15.06.2021, lgt. Rafał Gosik	Poland	*Salix* sp.		
6227	*R. pulicarius*	Serbia, Jokino Vrelo, Kremna, Mt. Tara 24.06.2021, lgt. Toševski	Serbia	*Salix alba*		
6228	*R. pulicarius*	Serbia, Jokino Vrelo, Kremna, Mt. Tara 24.06.2021, lgt. Toševski	Serbia	*Salix alba*		
6229	*R. pulicarius*	Serbia, Jokino Vrelo, Kremna, Mt. Tara 24.06.2021, lgt. Toševski	Serbia	*Salix alba*		
4541	*R. pulicarius*	Serbia, Babin Zub, Stara Planina, N43 23.165 E22 35.549, 1250 m, 5.07.2017, lgt. Toševski	Serbia	*Salix caprea*	pul2_CAD (PV930515)	**1**
6391	*R. pulicarius*	Erba, Lago di Alserio, 17.07.2022, lgt. L. Diotti	Italy	no data	pul3_CAD (PV930491)	**3**
6393	*R. pulicarius*	Erba, Lago di Alserio, 17.07.2022, lgt. L. Diotti,	Italy	no data		
6394	*R. pulicarius*	Erba, Lago di Alserio, 17.07.2022, lgt. L. Diotti,	Italy	no data		
6392	*R. pulicarius*	Erba, Lago di Alserio, 17.07.2022, lgt. L. Diotti,	Italy	no data	pul4_CAD (PV930492)	**1**
6223	*R. pulicarius*	Poland, Rudnik ad Lublin, 51°16'58.8"N 22°38'28.8"E, 15.06.2021, lgt. Rafał Gosik	Poland	*Betula* sp.	pul5_CAD (PV930516)	**1**
7442	*R. pulicaries*	Greece, Prodromis, mined leave of *Salix* sp., GPS 006 N40 27.650 E23 23.248, 28.09.2024, lgt. Toševski	Greece	*Salix* sp.	pul6_CAD (PV930517)	**6**
7443	*R. pulicaries*	Greece, Prodromis, mined leave of *Salix* sp., GPS 006 N40 27.650 E23 23.248, 28.09.2024, lgt. Toševski	Greece	*Salix* sp.		
7444	*R. pulicaries*	Greece, Prodromis, mined leave of *Salix* sp., GPS 006 N40 27.650 E23 23.248, 28.09.2024, lgt. Toševski	Greece	*Salix* sp.		
7445	*R. pulicaries*	Greece, Prodromis, mined leave of *Salix* sp., GPS 006 N40 27.650 E23 23.248, 28.09.2024, lgt. Toševski	Greece	*Salix* sp.		
7446	*R. pulicaries*	Greece, Prodromis, mined leave of *Salix* sp., GPS 006 N40 27.650 E23 23.248,	Greece	*Salix* sp.		

		28.09.2024, lgt. Toševski				
7447	*R. pulicaries*	Greece, Prodromis, mined leave of *Salix* sp., GPS 006 N40 27.650 E23 23.248, 28.09.2024, lgt. Toševski	Greece	*Salix* sp.		
						**23**

Rhamphus pullus Hustache, 1920

Voucher DNA	Species	Location	Origin	Host	CAD haplotype name (accession number)	Frequency
6334	*R. pullus*	Japan, Mikuni pass., Yamanakako vlg., Yamanashi pref., 12.06.2021, lgt. Y. Notsu	Japan	*Betula* sp.	pull1_CAD (PV930493)	**2**
6336	*R. pullus*	Japan, Mikuni pass., Yamanakako vlg., Yamanashi pref., 12.06.2021, lgt. Y. Notsu	Japan	*Betula* sp.		
6337	*R. pullus*	Japan, Mikuni pass., Yamanakako vlg., Yamanashi pref., 12.06.2021, lgt. Y. Notsu	Japan	*Betula* sp.	pull2_CAD (PV930494)	**1**
						**3**

Rhamphus betulae sp. n. Toševski & Caldara

Voucher DNA	Species	Location	Origin	Host	CAD haplotype name (accession number)	Frequency
6218	*R. betulae* sp.n. PARATYPE	Poland, Rudnik ad Lublin 51°16'58.8"N 22°38'28.8"E, 15.06.2021, lgt. Rafal Gosik	Poland	*Betula* sp.	bet1_CAD (PV930518)	**14**
6219	*R. betulae* sp.n. PARATYPE	Poland, Rudnik ad Lublin 51°16'58.8"N 22°38'28.8"E, 15.06.2021, lgt. Rafal Gosik	Poland	*Betula* sp.		
6220	*R. betulae* sp.n. PARATYPE	Poland, Rudnik ad Lublin 51°16'58.8"N 22°38'28.8"E, 15.06.2021, lgt. Rafal Gosik	Poland	*Betula* sp.		
6221	*R. betulae* sp.n. PARATYPE	Poland, Rudnik ad Lublin 51°16'58.8"N 22°38'28.8"E, 15.06.2021, lgt. Rafal Gosik	Poland	*Betula* sp.		
6224	*R. betulae* sp.n. PARATYPE	Poland, Rudnik ad Lublin 51°16'58.8"N 22°38'28.8"E, 15.06.2021, lgt. Rafal Gosik	Poland	*Betula* sp.		

6316	*R. betulae* sp.n. HOLOTYPE	Italy, Val Sesia, (VC) Monte Tovo, 1100 m, 12.06,2021, lgt. L. Diotti	Italy	*Betula pendula*		
6317	*R. betulae* sp.n. PARATYPE	Italy, Val Sesia, (VC) Monte Tovo, 1100 m, 12.06,2021, lgt. L. Diotti	Italy	*Betula pendula*		
6318	*R. betulae* sp.n. PARATYPE	Italy, Val Sesia, (VC) Monte Tovo, 1100 m, 12.06,2021, lgt. L. Diotti	Italy	*Betula pendula*		
6319	*R. betulae* sp.n. PARATYPE	Italy, Val Sesia, (VC) Monte Tovo, 1100 m, 12.06,2021, lgt. L. Diotti	Italy	*Betula pendula*		
6315	*R. betulae* sp.n. PARATYPE	Italy, Val Sesia, (VC) Monte Tovo, 1100 m, 12.06,2021, lgt. L. Diotti	Italy	*Betula pendula*		
6321	*R. betulae* sp.n. PARATYPE	Italy, Val Sesia, (VC) Monte Tovo, 1100 m, 12.06,2021, lgt. L. Diotti	Italy	*Betula pendula*		
6609	*R. betulae* sp.n. PARATYPE	France, 65 Trébons, 43.0799872049 /0.113917399, 30.06.2023 bouhaben, lgt. D. Lessieur	France	*Betula* sp.		
6610	*R. betulae* sp.n. PARATYPE	France, 65 Trébons, 43.0799872049 /0.113917399, 30.06.2023 bouhaben, lgt. D. Lessieur	France	*Betula* sp.		
6612	*R. betulae* sp.n. PARATYPE	France, 65 Trébons, 43.0799872049 /0.113917399, 30.06.2023 bouhaben, lgt. D. Lessieur	France	*Betula* sp.		
6216	*R. betulae* sp.n. PARATYPE	Poland, Rudnik ad Lublin 51°16'58.8"N 22°38'28.8"E, 15.06.2021, lgt. Rafal Gosik	Poland	*Betula* sp.	bet2_CAD (PV930519)	**5**
6217	*R. betulae* sp.n. PARATYPE	Poland, Rudnik ad Lublin 51°16'58.8"N 22°38'28.8"E, 15.06.2021, lgt. Rafal Gosik	Poland	*Betula* sp.		
6222	*R. betulae* sp.n. PARATYPE	Poland, Rudnik ad Lublin 51°16'58.8"N 22°38'28.8"E, 15.06.2021, lgt. Rafal Gosik	Poland	*Betula* sp.		
6225	*R. betulae* sp.n. PARATYPE	Poland, Rudnik ad Lublin 51°16'58.8"N 22°38'28.8"E, 15.06.2021, lgt. Rafal Gosik	Poland	*Betula* sp.		
6320	*R. betulae* sp.n. PARATYPE	Italy, Val Sesia, (VC) Monte Tovo, 1100 m, 12.06,2021, lgt. L. Diotti	Italy	*Betula pendula*		
6322	*R. betulae* sp.n. PARATYPE	Italy, Val Sesia, (VC) Monte Tovo, 1100 m, 12.06,2021, lgt. L. Diotti	Italy	*Betula pendula*	bet3_CAD (PV930495)	**1**

						**20**

**_Rhamphus crypticus_ sp. n. Toševski & Caldara**						
**Voucher DNA**	**Species**	**Location**	**Origin**	**Host**	**CAD haplotype name (accession number)**	**Frequency**
6006	*R. crypticus* sp.n. PARATYPE	England, New Forest, S. Hants, SU2404, 13.07.2020, lgt. H. Mendel	England	*Salix* sp.	cry1_CAD (PV930496)	**20**
6007	*R. crypticus* sp.n. PARATYPE	England, New Forest, S. Hants, SU2404, 13.07.2020, lgt. H. Mendel	England	*Salix* sp.		
6008	*R. crypticus* sp.n. PARATYPE	England, New Forest, S. Hants, SU2404, 13.07.2020, lgt. H. Mendel	England	*Salix* sp.		
6009	*R. crypticus* sp.n. PARATYPE	England, New Forest, S. Hants, SU2404, 13.07.2020, lgt. H. Mendel	England	*Salix* sp.		
6018	*R. crypticus* sp.n. PARATYPE	France, F 19, La Tronche (19110), la croix de Layre, 16.VII.2020, lgt. D. Lessieur	France	no data		
6352	*R. crypticus* sp.n. **HOLOTYPE**	England, 21/023, Lynford, Mundford, West Norfolk (VC28), TL8294, lgt. H. Mendel	England	*Salix* sp.		
6354	*R. crypticus* sp.n. HOLOTYPE	England, 21/026, Denge Beach, East Kent (VC15), TLR0817 28.06.2021, lgt. H. Mendel	England	*Salix* sp.		
6355	*R. crypticus* sp.n. PARATYPE	England, 21/026, Denge Beach, East Kent (VC15), TLR0817 28.06.2021, lgt. H. Mendel	England	*Salix* sp.		
6356	*R. crypticus* sp.n. PARATYPE	England, 21/026, Denge Beach, East Kent (VC15), TLR0817, 28.06.2021, lgt. H. Mendel	England	*Salix* sp.		
6357	*R. crypticus* sp.n. PARATYPE	England, 21/026, Denge Beach, East Kent (VC15), TLR0817, 28.06.2021, lgt. H. Mendel	England	*Salix* sp.		
6358	*R. crypticus* sp.n. PARATYPE	England, 21/026, Denge Beach, East Kent (VC15), TLR0817, 28.06.2021, lgt. H. Mendel	England	*Salix* sp.		
6359	*R. crypticus* sp.n.	England, 21/026, Denge Beach, East Kent (VC15),	England	*Salix* sp.		

	PARATYPE	TLR0817, 28.06.2021, lgt. H. Mendel				
6360	*R. crypticus* sp.n. PARATYPE	England, 21/026, Denge Beach, East Kent (VC15), TLR0817, 28.06.2021, lgt. H. Mendel	England	*Salix* sp.		
6599	*R. crypticus* sp.n. PARATYPE	Spain, provincia de Soria, Vinuesa, río Revinuesa, 13-VII-2020, lgt. Iñigo Ugarte San Vicente & Fernando Salgueira	Spain	*Salix salviifolia*		
6600	*R. crypticus* sp.n. PARATYPE	Spain, provincia de Soria, Vinuesa, río Revinuesa, 13-VII-2020, lgt. Iñigo Ugarte San Vicente & Fernando Salgueira	Spain	*Salix salviifolia*		
6615	*R. crypticus* sp.n. PARATYPE	France, Hautes-Pyrénées (65) - Poueyferré, 43.112498397 /-0.093337389, 29.06.2023, Tourbiére de Lourdes, lgt. D. Lessieur	France	*Salix atrocinerea*		
6616	*R. crypticus* sp.n. PARATYPE	France, Hautes-Pyrénées (65) - Poueyferré, 43.112498397 /-0.093337389, 29.06.2023, Tourbiére de Lourdes, lgt. D. Lessieur	France	*Salix atrocinerea*		
6617	*R. crypticus* sp.n. PARATYPE	France, Hautes-Pyrénées (65) - Poueyferré, 43.112498397 /-0.093337389, 29.06.2023, Tourbiére de Lourdes, lgt. D. Lessieur	France	*Salix atrocinerea*		
6618	*R. crypticus* sp.n. PARATYPE	France, Hautes-Pyrénées (65) - Poueyferré, 43.112498397 /-0.093337389, 29.06.2023, Tourbiére de Lourdes, lgt. D. Lessieur	France	*Salix atrocinerea*		
6623	*R. crypticus* sp.n. PARATYPE	France - Hautes-Pyrénées (65) Banios, 43.0409039 /0.233232, 1.07.2023, lgt. D. Lessieur	France	*Salix atrocinerea*		
						**20**

Rhamphus monzinii **Pesarini & Diotti, 2012**						
**Voucher DNA**	**Species**	**Location**	**Origin**	**Host**	**CAD haplotype name (accession number)**	**Frequency**
4535	*R. monzinii*	Serbia, Mt. Zlatibor, 9.07.2017, N43 47.310 E19 43.721, 662 m, lgt. Toševski	Serbia	*Prunus cerasifera* *Pyrus* sp.	mon1_CAD (PV930520)	**22**
4538	*R. monzinii*	Serbia, Mt. Zlatibor, 9.07.2017, N43 47.310 E19 43.721, 662 m, lgt. Toševski	Serbia	*Prunus sp.* *Pyrus* sp.		
4539	*R. monzinii*	Serbia, Mt. Zlatibor, 9.07.2017, N43 47.310 E19 43.721, 662 m, lgt. Toševski	Serbia	*Prunus sp.* *Pyrus* sp.		
4857	*R. monzinii*	Serbia, Brusnik, Negotin, N44 6.489 E22 24.115, 322 m, 21.05.2018, lgt. Toševski	Serbia	*Cydonia oblonga*		
4858	*R. monzinii*	Serbia, Brusnik, Negotin, N44 6.489 E22 24.115, 322 m, 21.05.2018, lgt. Toševski	Serbia	*Cydonia oblonga*		
4859	*R. monzinii*	Serbia, Brusnik, Negotin, N44 6.489 E22 24.115, 322 m, 21.05.2018, lgt. Toševski	Serbia	*Cydonia oblonga*		
4881	*R. monzinii*	Serbia, Brusnik, Negotin, N44 6.489 E22 24.115, 322 m, 21.05.2018, lgt. Toševski	Serbia	*Prunus spinosa*		
4885	*R. monzinii*	Serbia, Brusnik, Negotin, N44 6.489 E22 24.115, 322 m., 21.05.2018, lgt. Toševski	Serbia	*Prunus spinosa*		
4886	*R. monzinii*	Serbia, Brusnik, Negotin, N44 6.489 E22 24.115, 322 m, 21.05.2018, lgt. Toševski	Serbia	*Prunus spinosa*		
4888	*R. monzinii*	Serbia, Brusnik, Negotin, N44 6.489 E22 24.115, 322 m, 21.05.2018, lgt. Toševski	Serbia	*Prunus spinosa*		
4889	*R. monzinii*	Serbia, Brusnik, Negotin, N44 6.489 E22 24.115, 322 m, 21.05.2018, lgt. Toševski	Serbia	*Prunus spinosa*		
4890	*R. monzinii*	Serbia, Brusnik, Negotin, N44 6.489 E22 24.115, 322 m, 21.05.2018, lgt. Toševski	Serbia	*Prunus spinosa*		

4893	*R. monzinii*	Serbia, Vlasina, Božićki Kanal, N42 40.997 E22 21.888, 1289 m, 21.06.2018, lgt. Toševski	Serbia	*Prunus cerasifera*		
4894	*R. monzinii*	Serbia, Vlasina, Božićki Kanal, N42 40.997 E22 21.888, 1289 m, 21.06.2018, lgt. Toševski	Serbia	*Prunus cerasifera*		
4897	*R. monzinii*	Serbia, Vranje, Devotin, N42 36.511 E21 52.252, 991 m, 22.06.2018, lgt. Toševski	Serbia	*Prunus spinosa*		
5175	*R. monzinii*	Italy, Liguria, (GE), Piani di Creto, 23.06.2018 lgt. L. Diotti	Italy	-		
5258	*R. monzinii*	Serbia, Brusnik, Negotin, N44 6.489 E22 24.115, 322 m, 21.05.2018, lgt. Toševski	Serbia	*Prunus spinosa*		
5997	*R. monzinii*	Italy, Liguria, (GE), Piani di Creto, 23.06.2018 lgt. L. Diotti	Italy	no data		
5998	*R. monzinii*	Italy, Liguria, (GE), Piani di Creto, 23.06.2018 lgt. L. Diotti	Italy	no data		
5999	*R. monzinii*	Italy, Liguria, (GE), Piani di Creto, 23.06.2018, lgt. L. Diotti	Italy	no data		
6000	*R. monzinii*	Italy, Liguria, (GE), Piani di Creto, 23.06.2018 lgt. L. Diotti	Italy	no data		
6592	*R. monzinii*	Greece, Arethousa, 23.06.2023, Macedonia, N40 44.299 E23 34.854, lgt. Toševski	Greece	*Prunus spinosa*		
4906	*R. monzinii*	Greece, Mt. Taygetos 10.07.2018, N37 04.155 E22 15.882, lgt. Toševski	Greece	*Prunus spinosa*	mon2_CAD (PV930521)	**8**
6585	*R. monzinii*	Greece, Arethousa, 23.06.2023, Macedonia, N40 44.299 E23 34.854, lgt. Toševski	Greece	*Prunus spinosa*		
6586	*R. monzinii*	Greece, Arethousa, 23.06.2023, Macedonia, N40 44.299 E23 34.854, lgt. Toševski	Greece	*Prunus spinosa*		
6587	*R. monzinii*	Greece, Arethousa, 23.06.2023, Macedonia, N40 44.299 E23 34.854, lgt. Toševski	Greece	*Prunus spinosa*		

6588	*R. monzinii*	Greece, Arethousa, 23.06.2023, Macedonia, N40 44.299 E23 34.854, lgt. Toševski	Greece	*Prunus spinosa*		
6589	*R. monzinii*	Greece, Arethousa, 23.06.2023, Macedonia, N40 44.299 E23 34.854, lgt. Toševski	Greece	*Prunus spinosa*		
6590	*R. monzinii*	Greece, Arethousa, 23.06.2023, Macedonia, N40 44.299 E23 34.854, lgt. Toševski	Greece	*Prunus spinosa*		
6591	*R. monzinii*	Greece, Arethousa, 23.06.2023, Macedonia, N40 44.299 E23 34.854, lgt. Toševski	Greece	*Prunus spinosa*		
5176	*R. monzinii*	Italy, Liguria, (GE), Piani di Creto, 23.06.2018 lgt. L. Diotti	Italy	no data	mon3_CAD (PV930497)	**1**
5174	*R. monzinii*	Italy, Liguria, (GE), Piani di Creto, 23.06.2018 lgt. L. Diotti	Italy	no data	mon4_CAD (PV930498)	**1**
						**32**

Rhamphus diottii **sp. n. Toševski & Caldara**

**Voucher DNA**	**Species**	**Location**	**Origin**	**Host**	**CAD haplotype name (accession number)**	**Frequency**
4409	*R. diottii* sp.n. PARATYPE	Serbia, Slankamen Vinogradi, 29.05.2009, N45 9.715 E20 11.750, 224 m., lgt. Toševski	N. Serbia	*Prunus spinosa*	dio1_CAD (PV930522)	**10**
4542	*R. diottii* sp.n. PARATYPE	Serbia, Slankamen Vinogradi, 10.06.2017, N45 9.715 E20 11.750, 224 m., lgt. Toševski	N. Serbia	*Prunus spinosa*		
5262	*R. diottii* sp.n. PARATYPE	Serbia, Slankamen Vinogradi, 29.05.2009, N45 9.715 E20 11.750, 224 m., lgt. Toševski	N. Serbia	*Prunus spinosa*		
5263	*R. diottii* sp.n. PARATYPE	Serbia, Slankamen Vinogradi, 29.05.2009, N45 9.715 E20 11.750, 224 m., lgt. Toševski	N. Serbia	*Prunus spinosa*		
5264	*R. diottii* sp.n. PARATYPE	Serbia, Slankamen Vinogradi, 29.05.2009, N45 9.715 E20 11.750, 224 m., lgt. Toševski	N. Serbia	*Prunus spinosa*		

5266	*R. diottii* sp.n. PARATYPE	Serbia, Slankamen Vinogradi, 10.06.2017, N45 9.715 E20 11.750, 224 m., lgt. Toševski	N. Serbia	*Prunus spinosa*		
5267	*R. diottii* sp.n. PARATYPE	Serbia, Slankamen Vinogradi, 10.06.2017, N45 9.715 E20 11.750, 224 m., lgt. Toševski	N. Serbia	*Prunus spinosa*		
5268	*R. diottii* sp.n. PARATYPE	Serbia, Slankamen Vinogradi, 10.06.2017, N45 9.715 E20 11.750, 224 m., lgt. Toševski	N. Serbia	*Prunus spinosa*		
5269	*R. diottii* sp.n. PARATYPE	Serbia, Slankamen Vinogradi, 29.05.2009, N45 9.715 E20 11.750, 224 m., lgt. Toševski	N. Serbia	*Prunus spinosa*		
5270	*R. diottii* sp.n. HOLOTYPUS	Serbia, Slankamen Vinogradi, 29.05.2009, N45 9.715 E20 11.750, 224 m., lgt. Toševski	N. Serbia	*Prunus spinosa*		
4410	*R. diottii* sp.n. PARATYPE	Serbia, Slankamen Vinogradi, 29.05.2009, N45 9.715 E20 11.750, 224 m., lgt. Toševski	N. Serbia	*Prunus spinosa*	dio2_CAD (PV930523)	**2**
5265	*R. diottii* sp.n. PARATYPE	Serbia, Slankamen Vinogradi, 10.06.2017, N45 9.715 E20 11.750, 224 m., lgt. Toševski	N. Serbia	*Prunus spinosa*		
						**12**

Rhamphus ibericus **sp. n. Toševski & Caldara**

**Voucher DNA**	**Species**	**Location**	**Origin**	**Host**	**CAD haplotype name (accession number)**	**Frequency**
6593	*R. ibericus* sp.n. HOLOTYPE	España, País Vasco, Araba/Álava, Elburgo-Burgelu, 556 m s.n.m., N 46º50'26.27'' W 02º33'10.55'', 15.06.2023, lgt. Iñigo Ugarte San Vicente & Fernando Salgueira	Spain	*Prunus spinosa*	ibe1_CAD (PV930524)	**1**
6598	*R. ibericus* sp.n. PARATYPE	España, País Vasco, Araba/Álava, Elburgo-Burgelu, 556 m s.n.m., N 46º50'26.27'' W 02º33'10.55'', 15.06.2023, lgt. Iñigo Ugarte San	Spain	*Prunus spinosa*	ibe2_CAD (PV930525)	**1**

		Vicente & Fernando Salgueira				
6594	*R. ibericus* sp.n. PARATYPE	España, País Vasco, Araba/Álava, Elburgo-Burgelu, 556 m s.n.m., N 46º50'26.27'' W 02º33'10.55'', 15.06.2023, lgt. Iñigo Ugarte San Vicente & Fernando Salgueira	Spain	*Prunus spinosa*	ibe3_CAD (PV930526)	**1**
						**3**

***Rhamphus subaeneus* Illiger, 1808**						
**Voucher DNA**	**Species**	**Location**	**Origin**	**Host**	**CAD haplotype name (accession number)**	**Frequency**
5331	*R. subaeneus*	Spain, San Vicente de Arana, La Dehesa Álava, 8.07.2018, lgt. Iñigo Ugarte San Vicente & Fernando Salgueira	Spain	*Crataegus monogyna*	sub1_CAD (PV930499)	**4**
5337	*R. subaeneus*	Spain, San Vicente de Arana, La Dehesa, Álava, 7.07.2018, lgt. Iñigo Ugarte San Vicente & Fernando Salgueira	Spain	*Crataegus monogyna*		
5338	*R. subaeneus*	Spain, San Vicente de Arana, La Dehesa, Álava, 7.07.2018, lgt. Iñigo Ugarte San Vicente & Fernando Salgueira	Spain	*Crataegus monogyna*		
6601	*R. subaeneus*	España, País Vasco, provincia de Araba/Álava, Subijana de Álava, 518 m s.n.m., 25-VI-2019, lgt. Iñigo Ugarte San Vicente & Fernando Salgueira	Spain	*Crataegus monogyna*		
5336	*R. subaeneus*	Spain, San Vicente de Arana, La Dehesa, Álava, 7.07.2018, lgt. Iñigo Ugarte San Vicente & Fernando Salgueira	Spain	*Crataegus monogyna*	sub2_CAD (PV930500)	**1**
6094	*R. subaeneus*	Czech Rep., Bohemia Centr., Chramosty, Brdce hill, N49°40'12", E14°19'57", 415 m., 21.05.2020, lgt J. Kratky	Czechia	no data	sub3_CAD (PV930527)	**1**

						**6**

Rhamphus cerdanicus Tempère, 1982

Voucher DNA	Species	Location	Origin	Host	COI haplotype name (accession number)	Frequency
6595	*R. cerdanicus*	Spain, País Vasco, Araba/Álava, Elburgo-Burgelu, 556 m s.n.m., N 46º50'26.27'' W 02º33'10.55'', 15.06.2023, lgt. Iñigo Ugarte San Vicente & Fernando Salgueira	Spain	*Prunus spinosa*	Cer1_CAD (PV930528)	**1**
6596	*R. cerdanicus*	Spain, País Vasco, Araba/Álava, Elburgo-Burgelu, 556 m s.n.m., N 46º50'26.27'' W 02º33'10.55'', 15.06.2023, lgt. Iñigo Ugarte San Vicente & Fernando Salgueira	Spain	*Prunus spinosa*	Cer2_CAD (PV930529)	**1**
6597	*R. cerdanicus*	Spain, País Vasco, Araba/Álava, Elburgo-Burgelu, 556 m s.n.m., N 46º50'26.27'' W 02º33'10.55'', 15.06.2023, lgt. Iñigo Ugarte San Vicente & Fernando Salgueira	Spain	*Prunus spinosa*	Cer3_CAD (PV930530)	**1**
						**3**

Rhamphus loebli Germann & Colonnelli, 2018

Voucher DNA	Species	Location	Origin	Host	CAD haplotype name (accession number)	Frequency
6179	*R. loebli*	Spain, E. Andalucia, 4 km N of Rociana del Condado, N37 20.561 E6 36.165, 10.03.2011, lgt. J. Kratky	Spain	*Halimium sp.*	loe1_CAD (PV930531)	**2**

6580	*R. loebli*	Spain, E. Castilia, Srr. Francia, La Alberca, env., 1075 m, 40°31'49"N, 06°08'44"W, 25.05.2019, lgt. J. Kratky	Spain	*Halimium sp.*		
7352	*R. loebli*	Spain, E. Andalucia, 4 km N of Rociana del Condado, 37.344 N 6.596 W, 10.03.2011, lgt. J. Kratky	Spain	*Halimium sp.*	loe2_CAD (PV930532)	**1**
						**3**

Rhamphus hisamatsui Chûjô & Morimoto, 1960

Voucher DNA	Species	Location	Origin	Host	CAD haplotype name (accession number)	Frequency
6328	*R. hisamatsui*	Japan, Inugoeji forest, Yamakita town, Kanagawa pref., 28.06.2021, lgt. Y. Notsu	Japan	*Acer pictum* ssp. *mono*	his1_CAD (PV930501)	**1**
6331	*R. hisamatsui*	Japan, Inugoeji forest, Yamakita town, Kanagawa pref., 28.06.2021, lgt. Y. Notsu	Japan	*Acer pictum* ssp. *mono*	his2_CAD (PV930502)	**1**
6332	*R. hisamatsui*	Japan, Inugoeji forest, Yamakita town, Kanagawa pref., 28.06.2021, lgt. Y. Notsu	Japan	*Acer pictum* ssp. *mono*	his3_CAD (PV930503)	**1**
6329	*R. hisamatsui*	Japan, Inugoeji forest, Yamakita town, Kanagawa pref., 28.06.2021, lgt. Y. Notsu	Japan	*Acer pictum* ssp. *mono*	his4_CAD (PV930504)	**3**
6330	*R. hisamatsui*	Japan, Inugoeji forest, Yamakita town, Kanagawa pref., 28.06.2021, lgt. Y. Notsu	Japan	*Acer pictum* ssp. *mono*		
6333	*R. hisamatsui*	Japan, Inugoeji forest, Yamakita town, Kanagawa pref., 28.06.2021, lgt. Y. Notsu	Japan	*Acer pictum* ssp. *mono*		
6335	*R. hisamatsui*	Japan, Mikuni pass., Yamanakako vlg., Yamanashi pref., 12.06.2021, lgt. Y. Notsu	Japan	*Acer pictum* ssp. *mono*	his5_CAD (PV930505)	**1**
						**7**

						**238**
