# Supplementary material for: An Integrative Revision of the Genus Rhamphus (Curculionidae) from the Western Palearctic: Morphological and Molecular Data Reveal the Radiation of Multiple Species
Source: Insects. 2025 Nov 3;16(11):1123. doi: 10.3390/insects16111123 (PMC12653807; doi:10.3390/insects16111123)
Supplement: Supplementary file 1 [file insects-16-01123-s001.zip › Table_S4.pdf]

Table S4. Thermal protocol and reaction mix protocol for amplification of mt*COI*, n*EF-1α* and n*CAD* gene.

**Thermal protocol**					
	Initial denaturation	40 cycles			Final extension
		denaturation	annealing	extension	
mt*COI*	95°C/5 min	95°C/60 s	54°C/60 s	72°C/90 s	72°C/10 min
n*EF-1α*	95°C/5 min	95°C/45 s	52°C/60 s	68°C/90 s	68°C/10 min
n*CAD*	95°C/5 min	95°C/60 s	53°C/60 s	68°C/60 s	68°C/10 min
All DNA markers and short fragments*	95°C/5 min	95°C/45 s	52–54°C/30 s	68°C/30–60 s	68°C/10 min

**Reaction mix protocol**
FastGene *Taq* DNA polymerase (NIPPON Genetics, Duren, Germany)
20 µl reaction volume, High Yield Reaction Buffer A (1× with 1.5 mM MgCl2), additional 2.25 mM MgCl2, 0.6 mM each dNTP, 0.5 µM each primer, 1U of *Taq* DNA polymerase, 1 µl of DNA
FastGene Optima HotStart Ready Mix (NIPPON Genetics, Dueren, Germany)
25 µl reaction volume, 12.5 µl of 2X Optima HotStart ReadyMix with dye and 0.5 µM each primer, 1 µl of DNA

*When employing FastGene Optima HotStart ReadyMix
