# Supplementary material for: An Integrative Revision of the Genus Rhamphus (Curculionidae) from the Western Palearctic: Morphological and Molecular Data Reveal the Radiation of Multiple Species
Source: Insects. 2025 Nov 3;16(11):1123. doi: 10.3390/insects16111123 (PMC12653807; doi:10.3390/insects16111123)
Supplement: Supplementary file 1 [file insects-16-01123-s001.zip › Table_S5.pdf]

Table S5. Primers used for mt*COI* amplification

**Mitochondrial cytochrome oxidase subunit I gene (mt*COI*)**		
Primer name	Primer sequence	Reference
LCO1490	GGTCAACAAATCATAAAGATATTGG	Folmer et al., 1994
HCO2198	TAAACTTCAGGCTGACCAAAAAATCA	
LepF1	ATTCAACCAATCATAAAGATATTGG	Hebert et al. 2004
LepR1	TAAACTTCTGGATGTCCAAAAAATCA	
LCO1490Hem	TTTCAACTAAYCATAARGATATYGG	Germain et al., 2003
HCO2198Hem	TAAACYTCDGGATGBCCAAARAATCA	

References:

Folmer, O., Black, M., Hoeh, W., Lutz, R. and Vrijenhoek, R. (1994) DNA primers for amplification of mitochondrial cytochrome c oxidase subunit I from diverse metazoanin vertebrates. *Molecular Marine Biology and Biotechnology*, 3, 294–299.

Germain, J. F., Chatot, C., Meusnier, I., Artige, E., Rasplus, J. Y., & Cruaud, A. (2013). Molecular identification of *Epitrix* potato flea beetles (Coleoptera: Chrysomelidae) in Europe and North America. *Bulletin of Entomological Research*, 103(3), 354-362.

Hebert P.D.N., Penton E.H., Burns J.M., Janzen D.H. & Hallwachs W. 2004. Ten species in one: DNA barcoding reveals cryptic species in the neotropical skipper butterfly *Astraptes fulgerator*. *Proceedings of the National Academy of Sciences* 101: 14812–14817. https://doi.org/10.1073/pnas.0406166101.
