# Supplementary material for: An Integrative Revision of the Genus Rhamphus (Curculionidae) from the Western Palearctic: Morphological and Molecular Data Reveal the Radiation of Multiple Species
Source: Insects. 2025 Nov 3;16(11):1123. doi: 10.3390/insects16111123 (PMC12653807; doi:10.3390/insects16111123)
Supplement: Supplementary file 1 [file insects-16-01123-s001.zip › Table_S6.pdf]

Table S6. Primers used for n*EF-1α* amplification

**Elongation factor 1-alfa gene (*EF*-1α)**		
Primer name	Primer sequence	Reference
EF1-Bf	AGAACGTGAACGTGGTATCA	Hernández-Vera et al., 2013
EF-Br	CTTGGAGTCACCAGCTACATAACC	
*Ef-f1	TCGAAACTGCCARGTACTACGTCN	This study
*Efr-f2	CCCGGACACAGAGATTTCATC	
*Ef-f3	GGTGAATTYGAGGCTGGTATTTCCN	
*Ef-f4	GAAGCTYTGGACTCKATCCTGCCN	
*Ef-r1	GYTTSACTCCAAGRGTGAAAGCN	
*Ef-r2	TGCCAGCCGGAGATGGGTACAAAN	
*Ef-r3	TACCTGAAGAGGAAGACGCAGAGN	
*Ef-r4	ACTTCAGTGGTAATGTTGGCAGG	

Primers highlighted in yellow are the main primers used for n*EF-1α* amplification. They are also highlighted in the Figure S1.
*Primers for short fragments amplification of n*EF-1α*. For the archival and the specimens with presumably fragmented DNA due to degradation, we designed primers for short fragments amplification.

Reference:

Hernández-Vera, G., Caldara, R., Toševski, I., & Emerson, B. C. (2013). Molecular phylogenetic analysis of archival tissue reveals the origin of a disjunct southern African–Palaearctic weevil radiation. *Journal of Biogeography*, 40(7), 1348-1359.
