# Supplementary material for: An Integrative Revision of the Genus Rhamphus (Curculionidae) from the Western Palearctic: Morphological and Molecular Data Reveal the Radiation of Multiple Species
Source: Insects. 2025 Nov 3;16(11):1123. doi: 10.3390/insects16111123 (PMC12653807; doi:10.3390/insects16111123)
Supplement: Supplementary file 1 [file insects-16-01123-s001.zip › Table_S7.pdf]

Table S7. Primers used for n*CAD* amplification

**Primers for amplification of Carbamoyl-phosphate synthetase 2 gene (CAD)**		
Primer name	Primer sequence	Littereature
CADforB2	GARAARGTNGCNCCNAGTATGGC	Dole et al., 2010
CADfor4	TGGAARGARGTBGARTACGARGTGGTYCG	Danforth et al., 2006
CADrev1mod	GCCATYRCYTCBCCYACRCTYTTCAT	
*CAD-f1	CTCAARTATCCGGTAATGGCBAG	This study
*CAD-f2	GCYTTTTCTCTTGGAGGACTMGG	
*CAD-f3	GTATGCAACAKGGAAAATGTAGA	
*CAD-f4	GGAGAGTCCATAGTTGTKGCTCC	
*CAD-f5	AAGTCAATGCYAGATTRTCCAGGAG	
*CAD-f6	TGGCAAGTAARGCCACAGGSTATCC	
*CAD-r1	CTVGCCATTACCGGATAYTTGAG	
*CAD-r2	CCKAGTCCTCCAAGAGAAAARGC	
*CAD-r3	TCTACATTTTCCMTGTTGCATAC	
*CAD-r4	GGAGCMACAACTATGGACTCTCC	
*CAD-r5	CTCCTGGAYAATCTRGCATTGACTT	
*CAD-r6	GGATASCCTGTGGCYTTACTTGCCA	

Primers highlighted in yellow are the main primers used for n*CAD* amplification. They are also highlighted in the Figure S2.
*Primers for short fragments amplification of n*CAD*. For the archival and the specimens with presumably fragmented DNA due to degradation, we designed primers for short fragments amplification.

References:

Danforth, B. N., Fang, J., & Sipes, S. (2006). Analysis of family-level relationships in bees (Hymenoptera: Apiformes) using 28S and two previously unexplored nuclear genes: CAD and RNA polymerase II. *Molecular Phylogenetics and Evolution*, 39(2), 358-372.

Dole, S. A., Jordal, B. H., & Cognato, A. I. (2010). Polyphyly of Xylosandrus Reitter inferred from nuclear and mitochondrial genes (Coleoptera: Curculionidae: Scolytinae). *Molecular Phylogenetics and Evolution*, 54(3), 773-782.
