# Supplementary material for: An Integrative Revision of the Genus Rhamphus (Curculionidae) from the Western Palearctic: Morphological and Molecular Data Reveal the Radiation of Multiple Species
Source: Insects. 2025 Nov 3;16(11):1123. doi: 10.3390/insects16111123 (PMC12653807; doi:10.3390/insects16111123)
Supplement: Supplementary file 1 [file insects-16-01123-s001.zip › Table_S8.pdf]

Tables S8. Pairwise analysis (p-distance method) among all recorded haplotypes of *Rhamphus* species grouped according to species entities

Table S8 a. Average mitochondrial DNA *cytochrome oxydase subunit I* (*COI* gene) divergence based on pairwise analysis (p-distance method) among all *Rhamphus* haplotypes grouped according to their species affiliation: oxy - *Rhamphus oxyacanthae*; bav - *R. bavierai*; ham - *R. hampsicora*; cyp -*R. cypricus* sp. n.: mac - *R. macedonicus* sp. n.; pul - *R. pulicarius*; pullus - *R. pullus*; bet - *R. betulae* sp. n.; cry - *R. crypticus* sp. n.; mon - *R. monzinii*; dio - *R. diottii* sp. n.; ibe - *R. ibericus* sp. n.: sub - *R. subaeneus*; cer - *R. cerdanicus*; loe - *R. loebli*; his - *hisamatsui*.

			P (SE)															
Species	d_1 (SE)	d_2 (SE)	1	2	3	4	5	6	7	8	9	10	11	12	13	14	15	16
1. oxy.	0.118 (0.008)	0.010 (0.002)		(0.012)	(0.013)	(0.013)	(0.013)	(0.013)	(0.013)	(0.013)	(0.013)	(0.013)	(0.013)	(0.013)	(0.013)	(0.014)	(0.015)	(0.015)
2. bav.		0.018 (0.003)	0.127		(0.008)	(0.012)	(0.012)	(0.013)	(0.013)	(0.013)	(0.012)	(0.013)	(0.012)	(0.013)	(0.013)	(0.014)	(0.014)	(0.013)
3. ham.		0.002 (0.003)	0.134	0.062		(0.013)	(0.013)	(0.013)	(0.013)	(0.013)	(0.013)	(0.012)	(0.012)	(0.013)	(0.013)	(0.014)	(0.014)	(0.013)
4. cyp.		0.003 (0.001)	0.121	0.126	0.132		(0.007)	(0.012)	(0.013)	(0.014)	(0.013)	(0.013)	(0.013)	(0.014)	(0.014)	(0.014)	(0.014)	(0.015)
5. mac.		0.006 (0.002)	0.138	0.122	0.132	0.042		(0.013)	(0.014)	(0.014)	(0.013)	(0.014)	(0.014)	(0.014)	(0.014)	(0.014)	(0.015)	(0.015)
6. pul.		0.004 (0.001)	0.135	0.132	0.131	0.125	0.129		(0.009)	(0.009)	(0.012)	(0.013)	(0.013)	(0.013)	(0.014)	(0.015)	(0.014)	(0.015)
7. pullus		0.011 (0.004)	0.134	0.135	0.123	0.133	0.138	0.069		(0.007)	(0.011)	(0.013)	(0.013)	(0.013)	(0.014)	(0.015)	(0.015)	(0.014)
8. bet.		0.004 (0.002)	0.135	0.135	0.127	0.135	0.138	0.068	0.043		(0.011)	(0.013)	(0.013)	(0.014)	(0.014)	(0.016)	(0.015)	(0.014)
9. cry.		0.007 (0.002)	0.133	0.126	0.119	0.131	0.134	0.112	0.109	0.105		(0.013)	(0.013)	(0.013)	(0.014)	(0.015)	(0.014)	(0.014)
10. mon.		0.018 (0.003)	0.146	0.136	0.125	0.160	0.163	0.141	0.138	0.145	0.139		(0.010)	(0.012)	(0.013)	(0.015)	(0.015)	(0.014)
11. dio.		0.006 (0.002)	0.132	0.128	0.129	0.146	0.151	0.131	0.129	0.131	0.135	0.096		(0.012)	(0.013)	(0.015)	(0.014)	(0.014)
12. ibe.		n/c	0.142	0.141	0.136	0.161	0.172	0.146	0.150	0.148	0.147	0.135	0.127		(0.014)	(0.014)	(0.015)	(0.014)
13. sub.		0.010 (0.003)	0.145	0.147	0.150	0.149	0.152	0.155	0.148	0.157	0.150	0.165	0.145	0.159		(0.014)	(0.015)	(0.014)
14.cer.		0.008 (0.003)	0.150	0.160	0.156	0.152	0.155	0.170	0.176	0.175	0.165	0.188	0.169	0.169	0.159		(0.013)	(0.014)
15. loe.		0.003 (0.002)	0.176	0.167	0.172	0.163	0.172	0.154	0.167	0.165	0.165	0.176	0.157	0.166	0.167	0.151		(0.014)
16. his.		n/c	0.160	0.138	0.148	0.166	0.171	0.165	0.155	0.155	0.157	0.173	0.165	0.161	0.152	0.159	0.159	

d1, divergence over all sequence pairs; d2, divergence over sequence pairs within groups; P, p-distance over sequence pairs between groups; SE, standard error.

Table S8 b. Average *Elongation factor 1-alfa* (*Ef 1-alfa* gene) divergence based on pairwise analysis (p-distance method) among all *Rhamphus* haplotypes grouped according to their species affiliation: oxy - *Rhamphus oxyacanthae*; bav - *R. bavierai*; ham - *R. hampsicora*; cyp -*R. cypricus* sp. n.: mac - *R. macedonicus* sp. n.; pul - *R. pulicarius*; pullus - *R. pullus*; bet - *R. betulae* sp. n.; cry - *R. crypticus* sp. n.; mon - *R. monzinii*; dio - *R. diottii* sp. n.; ibe - *R. ibericus* sp. n.: sub - *R. subaeneus*; cer - *R. cerdanicus*; loe - *R. loebli*; his - *hisamatsui*.

			P (SE)															
Species	d_1 (SE)	d_2 (SE)	1	2	3	4	5	6	7	8	9	10	11	12	13	14	15	16
1. oxy.	0.046 (0.005)	0.005 (0.002)		(0.004)	(0.005)	(0.007)	(0.007)	(0.009)	(0.009)	(0.009)	(0.009)	(0.009)	(0.008)	(0.009)	(0.010)	(0.011)	(0.013)	(0.012)
2. bav.		0.006 (0.003)	0.014		(0.004)	(0.006)	(0.006)	(0.008)	(0.009)	(0.009)	(0.008)	(0.008)	(0.008)	(0.008)	(0.010)	(0.010)	(0.013)	(0.012)
3. ham.		0.002 (0.002)	0.016	0.011		(0.007)	(0.007)	(0.009)	(0.009)	(0.009)	(0.009)	(0.008)	(0.008)	(0.009)	(0.010)	(0.010)	(0.013)	(0.012)
4. cyp.		0.004 (0.003)	0.029	0.021	0.028		(0.004)	(0.009)	(0.010)	(0.009)	(0.008)	(0.008)	(0.008)	(0.009)	(0.011)	(0.011)	(0.013)	(0.013)
5. mac.		n/c	0.029	0.022	0.028	0.010		(0.009)	(0.010)	(0.009)	(0.008)	(0.009)	(0.009)	(0.010)	(0.011)	(0.011)	(0.014)	(0.013)
6. pul.		0.007 (0.002)	0.052	0.047	0.051	0.055	0.056		(0.004)	(0.002)	(0.007)	(0.010)	(0.010)	(0.010)	(0.010)	(0.010)	(0.014)	(0.013)
7. pullus		0.006 (0.003)	0.050	0.046	0.048	0.058	0.058	0.014		(0.004)	(0.007)	(0.010)	(0.010)	(0.010)	(0.011)	(0.011)	(0.013)	(0.013)
8. bet.		0.006 (0.002)	0.052	0.047	0.050	0.055	0.056	0.006	0.013		(0.007)	(0.010)	(0.010)	(0.010)	(0.010)	(0.010)	(0.014)	(0.013)
9. cry.		0.004 (0.002)	0.049	0.043	0.048	0.046	0.045	0.031	0.033	0.030		(0.009)	(0.009)	(0.010)	(0.011)	(0.011)	(0.014)	(0.013)
10. mon.		0.005 (0.002)	0.046	0.037	0.038	0.041	0.041	0.060	0.062	0.059	0.054		(0.004)	(0.007)	(0.011)	(0.011)	(0.014)	(0.012)
11. dio.		0.003 (0.002)	0.046	0.038	0.038	0.042	0.041	0.062	0.064	0.062	0.053	0.011		(0.007)	(0.010)	(0.011)	(0.014)	(0.013)
12. ibe.		n/c	0.048	0.036	0.040	0.047	0.045	0.063	0.060	0.063	0.057	0.029	0.027		(0.010)	(0.011)	(0.014)	(0.014)
13. sub.		0.004 (0.002)	0.057	0.055	0.053	0.066	0.064	0.068	0.069	0.067	0.070	0.065	0.066	0.060		(0.009)	(0.014)	(0.014)
14. cer.		0.002 (0.002)	0.062	0.058	0.061	0.073	0.071	0.066	0.067	0.066	0.068	0.070	0.071	0.062	0.041		(0.014)	(0.014)
15. loe.		n/c	0.099	0.102	0.108	0.112	0.115	0.121	0.116	0.121	0.118	0.112	0.116	0.117	0.119	0.101		(0.013)
16. his.		n/c	0.094	0.094	0.097	0.106	0.108	0.114	0.115	0.114	0.113	0.103	0.108	0.117	0.122	0.113	0.108	

d1, divergence over all sequence pairs; d2, divergence over sequence pairs within groups; P, p-distance over sequence pairs between groups; SE, standard error.

Table S8 c. Average *Carbamoyl-Phosphate Synthetase* 2 (*CAD* gene) divergence based on pairwise analysis (p-distance method) among all *Rhamphus* haplotypes grouped according to their species affiliation: oxy - *Rhamphus oxyacanthae*; bav - *R. bavierai*; ham - *R. hampsicora*; cyp - *R. cypricus* sp. n.: mac - *R. macedonicus* sp. n.; pul - *R. pulicarius*; pullus - *R. pullus*; bet - *R. betulae* sp. n.; cry - *R. crypticus* sp. n.; mon - *R. monzinii*; dio - *R. diottii* sp. n.; ibe - *R. ibericus* sp. n.: sub - *R. subaeneus*; cer - *R. cerdanicus*; loe - *R. loebli*; his - *hisamatsui*.

			P (SE)															
Species	d_1 (SE)	d_2 (SE)	1	2	3	4	5	6	7	8	9	10	11	12	13	14	15	16
1. oxy.	0.083 (0.007)	0.003 (0.002)		(0.003	(0.004	(0.009	(0.009	(0.010)	(0.011)	(0.010)	(0.009)	(0.010)	(0.009)	(0.009)	(0.013	(0.012)	(0.015)	(0.017)
2. bav.		0.002 (0.002)	0.008		(0.004	(0.009	(0.009	(0.010)	(0.011)	(0.011)	(0.009)	(0.010)	(0.010)	(0.010)	(0.012	(0.012)	(0.015)	(0.017)
3. ham.		0.002 (0.002)	0.010	0.009		(0.010	(0.010	(0.010)	(0.011)	(0.011)	(0.010)	(0.010)	(0.010)	(0.010)	(0.013	(0.013)	(0.015)	(0.017)
4. cyp.		0.004 (0.002)	0.050	0.055	0.057		(0.003	(0.010)	(0.011)	(0.011)	(0.009)	(0.009)	(0.009)	(0.009)	(0.012	(0.012)	(0.014)	(0.017)
5. mac.		n/c	0.051	0.056	0.058	0.005		(0.011)	(0.012)	(0.011)	(0.009)	(0.009)	(0.009)	(0.009)	(0.012	(0.012)	(0.015)	(0.017)
6. pul.		0.005 (0.002)	0.055	0.058	0.061	0.056	0.057		(0.005)	(0.006)	(0.008)	(0.011)	(0.011)	(0.010)	(0.013	(0.012)	(0.015)	(0.017)
7. pullus		0.002 (0.002)	0.064	0.068	0.070	0.063	0.064	0.017		(0.007)	(0.009)	(0.012)	(0.012)	(0.011)	(0.014	(0.013)	(0.015)	(0.017)
8. bet.		0.003 (0.002)	0.057	0.062	0.064	0.054	0.054	0.020	0.025		(0.008)	(0.012)	(0.012)	(0.010)	(0.014	(0.013)	(0.015)	(0.017)
9. cry.		n/c	0.046	0.049	0.051	0.044	0.041	0.033	0.040	0.031		(0.010)	(0.009)	(0.008)	(0.013	(0.012)	(0.015)	(0.017)
10. mon.		0.008 (0.003)	0.059	0.063	0.059	0.048	0.043	0.067	0.079	0.074	0.056		(0.007)	(0.009)	(0.012	(0.011)	(0.015)	(0.016)
11. dio.		0.002 (0.002)	0.055	0.059	0.061	0.047	0.042	0.062	0.070	0.069	0.051	0.027		(0.009)	(0.011	(0.011)	(0.015)	(0.016)
12. ibe.		0.006 (0.003)	0.054	0.061	0.063	0.044	0.042	0.057	0.065	0.055	0.038	0.051	0.048		(0.012	(0.011)	(0.015)	(0.017)
13. sub.		0.003 (0.002)	0.084	0.083	0.090	0.071	0.069	0.088	0.094	0.094	0.078	0.071	0.068	0.078		(0.009)	(0.015)	(0.016)
14. cer.		0.007 (0.003)	0.081	0.079	0.088	0.072	0.072	0.082	0.088	0.089	0.073	0.078	0.074	0.074	0.049		(0.015)	(0.017)
15. loe.		0.002 (0.002)	0.146	0.147	0.150	0.132	0.132	0.142	0.144	0.147	0.147	0.139	0.142	0.138	0.144	0.135		(0.017)
16. his.		0.009 (0.003)	0.169	0.175	0.171	0.182	0.172	0.173	0.173	0.179	0.179	0.169	0.171	0.177	0.180	0.176	0.198	

d1, divergence over all sequence pairs; d2, divergence over sequence pairs within groups; P, p-distance over sequence pairs between groups; SE, standard error.
